# Impact of a multicomponent strategy including decentralized molecular testing for tuberculosis on mortality: planned analysis of a cluster-randomized trial in Uganda

**DOI:** 10.1016/j.eclinm.2024.102953

**Published:** 2024-11-26

**Authors:** Achilles Katamba, Tessa Mochizuki, Talemwa Nalugwa, Mariam Nantale, Denis Oyuku, Sarah Nabwire, Diana Babirye, Johnson Musinguzi, Annet Nakawesa, Irene Nekesa, Stavia Turyahabwe, Moses Joloba, David W. Dowdy, David A.J. Moore, J. Lucian Davis, Priya Shete, Katherine Adams, Tania Reza, Katherine Fielding, Adithya Cattamanchi

**Affiliations:** a Uganda Tuberculosis Implementation Research Consortium, Walimu, Kampala, Uganda; b Clinical Epidemiology and Biostatistics Unit, Department of Medicine, Makerere University College of Health Sciences, Kampala, Uganda; c Division of Pulmonary and Critical Care Medicine, San Francisco General Hospital, University of California San Francisco, San Francisco, USA; d Center for Tuberculosis, University of California San Francisco, San Francisco, USA; e Uganda Ministry of Health, National Tuberculosis and Leprosy Program, Kampala, Uganda; f School of Biomedical Sciences, Makerere University College of Health Sciences, Kampala, Uganda; g Department of Epidemiology, Johns Hopkins Bloomberg School of Public Health, Baltimore, MD, USA; h London School of Hygiene & Tropical Medicine, London, UK; i Pulmonary, Critical Care, and Sleep Medicine Section, Yale University, New Haven, CT, USA; j Division of Pulmonary Diseases and Critical Care Medicine, University of California Irvine, Irvine, CA, USA

**Keywords:** Xpert, Tuberculosis, Diagnostic trials

## Abstract

**Background:**

Rapid diagnosis of tuberculosis (TB) is important for improving outcomes and reducing transmission. Previous studies assessing the impact of Xpert MTB/RIF (Xpert), a molecular assay that provides results within 2 h, on mortality have been inconclusive. In this planned analysis of a pragmatic cluster-randomized trial in Uganda, we assessed whether a multicomponent strategy, including decentralized Xpert testing, decreased mortality among adults evaluated for TB.

**Methods:**

Ten community health centers were randomized, using a computer-generated randomization sequence, to the XPEL-TB intervention (on-site Xpert testing plus implementation supports) and ten to routine TB care without any modifications (on-site smear microscopy and referral-based Xpert testing for selected patients). The trial included all adults (≥18 years of age) undergoing evaluation for presumptive TB at each trial health center. All-cause mortality was a secondary outcome of the trial. For this analysis, the primary outcome was the mortality rate (censored at 18 months), and the secondary outcome was the six-month mortality risk. We compared the outcomes between trial arms using cluster-level analyses to account for stratified randomization and patient-level covariates. The trial was registered with the US National Institutes of Health (identifier: NCT03044158) and the Pan African Clinical Trials Registry (identifier: PACTR201610001763265).

**Findings:**

Vital status was ascertained for 8413 of 9563 (88%) XPEL-TB trial participants who presented at the health centers from October 22, 2018 through February 29, 2020. The adjusted rate ratio (aRR) was 0.77 (95% CI: 0.47–1.28), comparing the intervention (145 deaths/3655 person-years) to routine care (154 deaths/3015 person-years). In sub-group analyses, point estimates for mortality were lower in the intervention arm among people without HIV (aRR = 0.50, 95% CI: 0.26–0.96) and among females (aRR = 0.64, 95% CI: 0.33–1.23). The mortality risk analysis yielded similar results.

**Interpretation:**

Consistent point estimates favoring the intervention in our trial and previous ones suggest that Xpert testing may have an impact on mortality at community health centers. However, the magnitude of effect is small, and statistically significant results are unlikely to be attained within a single trial. Future trials of novel TB diagnostics at community health centers should focus on more proximal outcomes including TB detection and treatment initiation.

**Funding:**

This work was supported by the 10.13039/100000050National Heart, Lung, and Blood Institute of the 10.13039/100000002US National Institutes of Health under award number R01HL130192.

**Research in context**

**Evidence before this study**

We searched Pubmed for papers evaluating the impact of Xpert on mortality, using the terms (“tuberculosis” OR “TB”) AND “mortality” AND (“GeneXpert” OR “Xpert”), with no language or date restrictions. The search yielded 338 results, which included nine studies evaluating the impact of Xpert MTB/RIF (Xpert) on mortality, in comparison to tuberculosis (TB) standard of care sputum-smear microscopy, and systematic reviews. Prior studies and meta-analyses that assessed the impact of Xpert on mortality, compared to sputum-smear microscopy, were inconclusive (pooled effect size 0.89, 95% CI 0.75–1.05). Some studies have shown an impact among people living with HIV, notably those with advanced disease. Modelling studies have suggested a decrease in mortality could be observed with rapid molecular diagnostics, but this has not been demonstrated conclusively in the published literature among ambulatory populations.

**Added value of this study**

This is the largest study to assess the impact of an Xpert-based intervention strategy on mortality at the community level. A low number of deaths was observed among people evaluated for TB at community health centers. While the mortality rate was reduced in the intervention arm, similar to previous trials and meta-analyses, the result was not statistically significant. Study findings suggest a possible impact among individuals without HIV, which may have implications for a broader population who is not yet engaged in care or may not have other comorbidities that increase their risk of death.

**Implications of all the available evidence**

Although our study and published meta-analyses on their own do not provide conclusive evidence of an effect on mortality, the consistent point estimates favoring a mortality reduction across studies suggest a likely population-level impact of Xpert testing. While mortality remains important to measure, any single study assessing the impact of novel TB diagnostics among ambulatory populations is likely to be underpowered. More proximal outcomes such as diagnostic yield and pre-treatment loss to follow-up, in conjunction with person-centered outcomes such as time to diagnosis, lost productivity while undergoing evaluation, and satisfaction with care, should drive policymaking and scale-up considerations.

## Introduction

Tuberculosis (TB) remains a leading cause of infectious disease death worldwide, with an estimated 1.3 million deaths due to TB in 2022.^1^ Rapid diagnosis followed by prompt treatment initiation is important for reducing morbidity and community transmission.^2^ To achieve these goals, the World Health Organization (WHO) has recommended rapid molecular tests such as Xpert MTB/RIF (Xpert) (Cepheid, Inc. Sunnyvale, California) as the first-line test for all people exhibiting signs and symptoms of TB since 2013 and has called for universal access to molecular testing.^3^, ^4^, ^5^ Molecular tests can provide results within 2 h and can detect both the presence of *Mycobacterium tuberculosis* and resistance to rifampicin, a key first-line anti-TB drug, helping to ensure patients initiate appropriate treatment. However, the impact of rapid molecular testing on mortality of people undergoing evaluation for TB remains unclear.

Nine previous studies have found inconclusive mortality reductions with Xpert testing in comparison to sputum smear microscopy.^6^, ^7^, ^8^, ^9^, ^10^, ^11^, ^12^, ^13^, ^14^, ^15^ Meta-analyses of previous trials have also not conclusively demonstrated that rapid molecular testing reduces mortality.^16^^,^^17^ However, previous trials had key limitations including insufficient sample size and study design features that could bias results toward the null. Previous trials included procedures such as chest x-ray, sputum culture, and additional contact with patients that would not have otherwise occurred as part of routine care, potentially leading to more patients being tested and treated for TB.^6^

To address many of the limitations described above, we conducted a highly pragmatic cluster-randomized trial in Uganda of a multicomponent strategy including decentralized Xpert testing in comparison to routine care (on-site smear microscopy plus referral-based Xpert testing for selected patients). Key pragmatic features of the trial included unbiased recruitment of persons with possible TB and reliance on routine clinicians to conduct all testing and treatment.^18^ Additional procedures that might increase empiric treatment, such as chest x-ray, were not incorporated, and there were no attempts to reduce loss to follow-up by adding culture testing or scheduled contact with study participants. The intervention strategy included setting up workflows to enable same-day treatment to mitigate pre-treatment loss to follow-up and performance feedback to improve the quality of care.

In the XPEL-TB trial, we previously reported that the multicomponent intervention strategy increased TB diagnosis and treatment initiation among adults presenting to health facilities in Uganda and being evaluated for TB.^19^ Here, we report on whether the XPEL-TB strategy, which included on-site Xpert testing as the first-line test for TB, decreased all-cause mortality among adults undergoing TB evaluation at community health centers.

## Methods

### Study design

The XPEL-TB trial was conducted at 20 community health centers in Uganda from October 22, 2018 through February 29, 2020. The study protocol has been described previously.^20^ In brief, ten health centers were randomized, using a computer-generated randomization sequence, to a multicomponent intervention, which included on-site Xpert testing with GeneXpert Edge machines and Xpert MTB/RIF Ultra cartridges, guided restructuring of clinic workflows to facilitate same-day TB diagnosis and treatment, and performance feedback to facilitate continuous process improvement. The other ten health centers continued routine TB care, which included on-site sputum smear microscopy and referral of sputum samples to centralized Xpert testing facilities for selected patients. Outcome collection was done through routine TB registers under a waiver of informed consent.^20^ The multicomponent intervention led to greater numbers of patients being diagnosed with and treated for confirmed TB within 14 days after presentation to the health center (adjusted rate ratio [aRR], 1.56; 95% confidence interval [CI], 1.21–2.01).^19^ Here, we report a planned analysis to assess the impact of the intervention strategy on a secondary outcome of all-cause mortality.

### Ethics

The XPEL-TB trial was approved by the institutional review boards at the University of California San Francisco (approval number: 17–21505), Makerere University College of Health Sciences (HDREC 595), and the Uganda National Council for Science and Technology (HS 2437). It is registered with the US National Institutes of Health (identifier: NCT03044158) and the Pan African Clinical Trials Registry (identifier: PACTR201610001763265) as a phase 4 clinical trial.

### Eligibility

The XPEL-TB trial included all adults (≥ 18 years of age) undergoing evaluation for presumptive TB at each trial health center, defined as having been entered into the National TB and Leprosy Program presumptive TB, TB laboratory, or TB treatment registers. These three registers include data on patients who screen positive for TB symptoms (presumptive), are tested for TB (laboratory), and are treated for TB (treatment). This analysis excluded trial participants for whom vital status could not be ascertained, or those known to be alive but with no valid date associated with the outcome, or HIV status was unknown.

### Procedures

Vital status assessment was attempted for all trial participants through review of TB treatment registers, phone calls, and home visits and planned for six months following initial TB evaluation. Follow-up continued through July 31, 2022 to maximize ascertainment of vital status. For people who initiated TB treatment, treatment registers were first reviewed to identify the date of treatment completion. For those who did not initiate or complete treatment, up to six phone calls were made by trained study staff using phone numbers recorded in health center TB registers. Calls were made at different times of the day to maximize the chance of reaching the intended person or next of kin. If the intended person or next of kin could not be reached by phone, staff enlisted community health workers at each of the centers to conduct home visits. For both phone calls and home visits, a standardized form was used to confirm identity, vital status, and date of death from next of kin if deceased. Vital status outcomes were documented using a secure, web-based REDCap electronic data capture tool hosted at the University of California, San Francisco.^21^^,^^22^

### Outcomes

The date of TB diagnostic evaluation was defined as the earliest date of TB screening, TB testing or treatment initiation as documented in the TB registers at the health centers. While the initial intent was to assess vital status at six months after TB diagnostic evaluation, interruptions caused by the COVID-19 pandemic delayed initiation of follow-up. Study staff continued to attempt ascertainment of vital status beyond the six-month time frame. We therefore analyzed mortality as both a rate and risk, with the latter measured at six months after the date of diagnostic evaluation.

For individuals with multiple sources of vital status information and discrepant dates of death or outcomes documented, home visit data were prioritized if available, followed by phone call data, and then TB register data. For individuals known to have died but with an unknown date of death, the date was assigned to be the midpoint between the date the individual was last known to be alive and the date the individual was first known to have died. More information on how outcomes were defined can be found in the Supplementary Methods. No imputation was done for those with missing vital status information due to the small number of covariates in this pragmatic study.

To estimate the mortality rate, follow-up was censored at 18 months (548 days) after TB diagnostic evaluation. If the trial participant had a date of death that was ≤548 days from TB evaluation, the death was included in this analysis. If the trial participant had a date of death that was >548 days after TB evaluation or the trial participant was confirmed to be alive, the individual contributed person-days at risk from the date of TB evaluation to the date last confirmed to be alive or 548 days from the TB evaluation date.

To estimate six-month mortality risk, we limited follow-up to 5–8 months (152–243 days) after TB evaluation. A trial participant was defined to have died if the date of death was ≤243 days after TB evaluation. If the trial participant had a date of death that was >243 days after the date of TB evaluation or there was no evidence the patient had died and the trial participant was last confirmed to be alive ≥152 days after TB evaluation, the trial participant was assumed to be alive at 152 days. If the date a trial participant was last confirmed to be alive was <152 days after TB evaluation, the trial participant had an unknown status for this endpoint.

### Statistics

We performed cluster-level analyses, taking into account the stratified randomization (fixed effect, two levels), to assess the effect of the intervention strategy on mortality.^23^ We calculated rate ratios and rate differences with adjustment for individual-level covariates available in the health center TB registers using a two-stage approach: at the individual-level Poisson regression was used to adjust for age, sex, and HIV status and a cluster-level covariate (number of individuals treated for confirmed TB during the 12-month pre-trial period); followed by analysis of observed and expected number of deaths, the latter obtained from the individual-level regression model, at the cluster-level. Adjustments were consistent with the primary trial analysis. We conducted pre-specified subgroup analyses by sex and HIV status. For clusters with zero outcomes in the subgroup analyses, we added +0.5 to all numerators. For the analysis of the six-month mortality risk, the adjusted analysis used a similar approach, using logistic regression. For all analyses, we reported point estimates along with their 95% confidence intervals.

Individuals without vital status outcomes, based on the specified definitions, and those with unknown HIV status were excluded from all primary analyses. Chi-squared analysis, with adjustment for clustering by health center, was used to compare those with and without vital status outcomes. Sensitivity analyses were conducted to assess the impact of the vital status definitions for each mortality endpoint (these included removing trial participants with a missing or invalid date of death and applying alternate outcomes or dates for a small proportion of individuals with multiple outcomes or dates available) and to assess the impact of the exclusion of trial participants without known HIV status.

Analyses were performed using Stata Version 17 (StataCorp, College Station, TX, USA).

### Role of funding source

This work was supported by the National Heart Lung and Blood Institute of the US National Institutes of Health under award number R01HL130192. The funders had no role in study design, data collection, data analysis, data interpretation, decision to publish, or preparation of the manuscript.

## Results

**Fig. 1:**
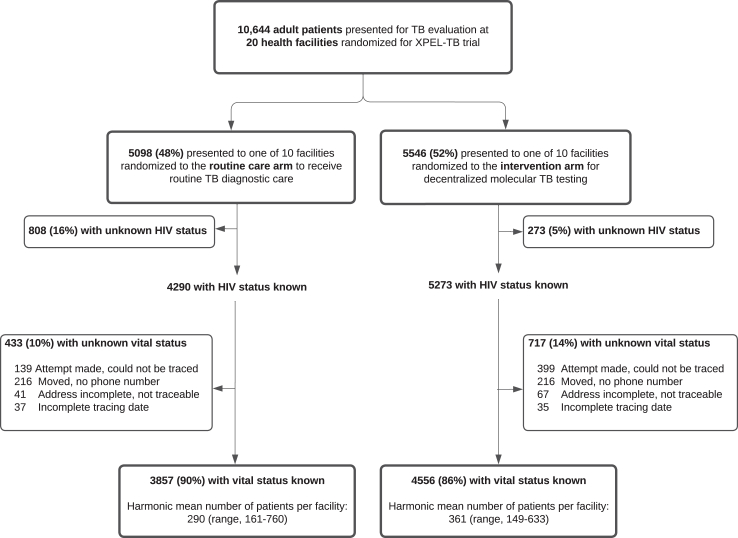
**Individuals, randomization, and vital status ascertainment for mortality rate outcome**. Abbreviation: TB, tuberculosis.

From October 22, 2018 through February 29, 2020, 10,644 eligible adults presented for TB evaluation across the 20 study sites and were included in the XPEL-TB trial (Fig. 1). Of those, 1081 (10%) had an unknown HIV status and were excluded from this analysis.

Overall, ascertainment of vital status was high for the remaining 9563 trial participants. For the mortality rate outcome that considered follow-up over 18 months, 88% of individuals had a known vital status (8413/9563), and this was comparable between the intervention and routine care arms (86% vs. 90%, p = 0.26) (Table 1). The primary reasons for unknown vital status were similar by trial arm: a) an attempt was made but the patient or next of kin could not be reached (n = 538) or b) the patient moved and no phone number was available (n = 432) (Fig. 1). Small differences in vital status ascertainment were observed by age at time of diagnostic evaluation; young adults (18–29 years) and older adults (50+ years) had a lower percentage of participants with vital status ascertained (p = 0.02). Vital status ascertainment varied by health center (range 71%–99%; Table S1), and most vital status outcomes were obtained through home visit (60%, Table S2). For the mortality risk outcome, a total of 8116 (85%) individuals had vital status known at six-months post-TB diagnostic evaluation (Table S4).

**Table 1:** Patient and cluster-level demographic and clinical characteristics.

	Overall N = 9563	Known vital status for mortality rate outcome N = 8413	Unknown vital status for mortality rate outcome N = 1150	p-value^a^
	N	N (%)	N (%)	
Trial arm				0.26
Intervention	5273	4556 (86.4)	717 (13.6)	
Routine care	4290	3857 (89.9)	433 (10.1)	
Sex				0.093
Female	5709	4981 (87.3)	728 (12.8)	
Male	3854	3432 (89.0)	422 (11.0)	
Age				0.016
18–29	2470	2133 (86.4)	337 (13.6)	
30–39	2188	1966 (89.9)	222 (10.2)	
40–49	2038	1808 (88.7)	230 (11.3)	
50+	2867	2506 (87.4)	361 (12.6)	
HIV status				0.12
Positive	4190	3746 (89.4)	444 (10.6)	
Negative	5373	4667 (86.9)	706 (13.1)	

a Adjusted for clustering by health center.

### Mortality rate outcome

**Fig. 2:**
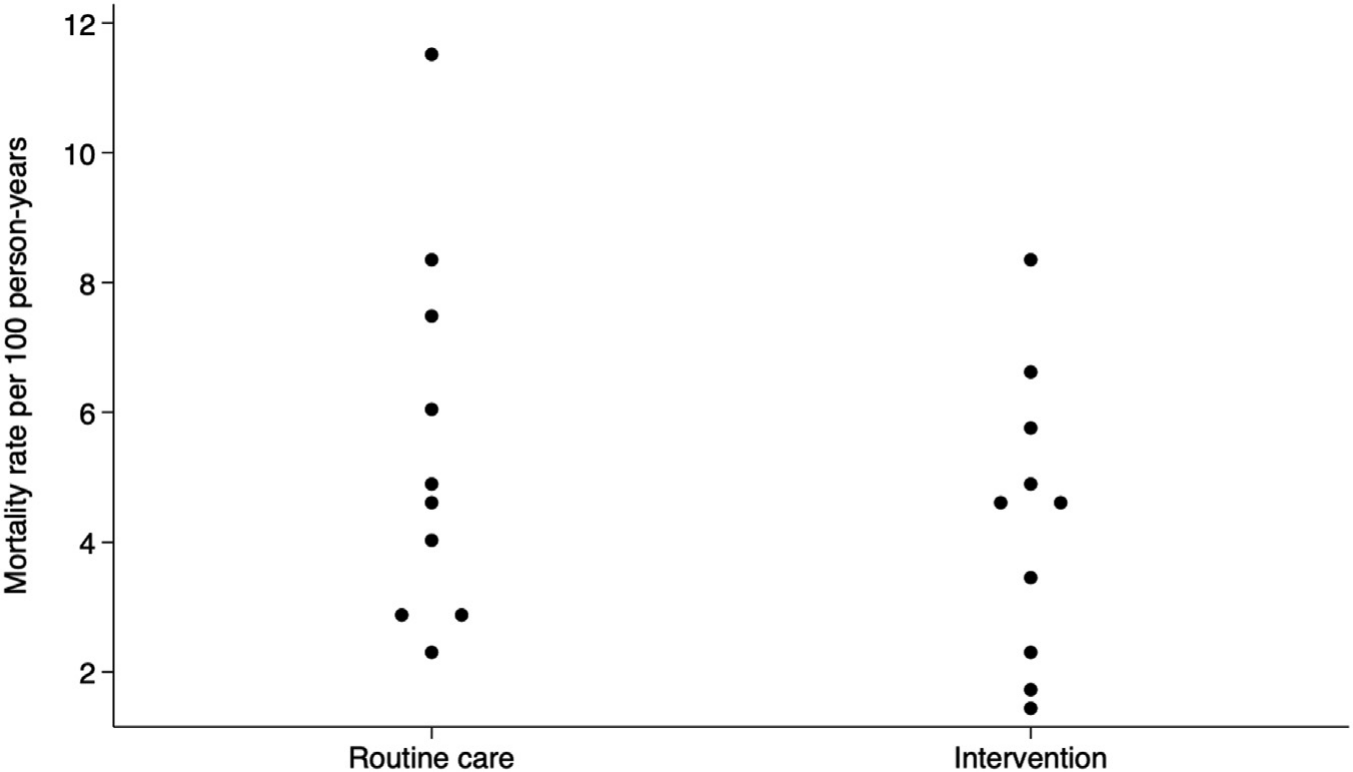
**Cluster-level mortality rate by trial arm (N = 8413)**. Footnote: Each point represents a cluster (health center). Health centers within trial arm that have the same mortality rate, or mortality rates similar enough to overlap on the graph, are displayed in a horizontal line.

The overall mortality rate in the intervention arm was 3.8 per 100 person-years, compared to 4.9 in the routine care arm (unadjusted rate; Table 2). Individuals in the intervention arm contributed 3655 person-years, compared to 3015 person-years in the routine care arm. Higher mortality rates were observed among people living with HIV (PLHIV) than people living without HIV (intervention: 6.7 vs. 2.2 per 100 person-years, routine care: 6.1 vs. 3.7 per 100 person-years) and among males compared to females (intervention: 5.6 vs. 2.4 per 100 person-years, routine care: 5.4 vs. 3.7 per 100 person-years). Unadjusted mortality rates varied by health center (Fig. 2).

**Table 2:** Unadjusted and adjusted mortality ratios for rate outcome, overall and by subgroup.

Mortality rate over 18 months (n = 8413)						
	Intervention		Routine care		Adjusted rate ratio (95% CI)^b^	p-value for interaction
	Deaths Observed/Person-years	Rate^a^	Deaths Observed/Person-years	Rate^a^		
Overall	145/3654.8	3.83	154/3015.3	4.88	0.77 (0.47–1.28)	
HIV status						0.042
Positive	99/1570.6	6.72	76/1348.6	6.10	1.10 (0.65–1.86)	
Negative	46/2084.2	2.16	78/1666.7	3.71	0.50 (0.26–0.96)	
Sex						0.19
Female	63/2178.0	2.36	81/1850.7	3.70	0.64 (0.33–1.23)	
Male	82/1476.8	5.56	73/1164.6	5.43	0.99 (0.53–1.82)	

a Unadjusted rates in the intervention and routine care arms are the geometric mean of the point estimates across the ten clusters in each group. Rates are reported per 100 person-years. The unadjusted rate ratio is 0.79 (0.46–1.33).

b Adjusted rate ratios compare the intervention vs. routine care arm and analysis was done at the cluster-level with adjustment for randomization strata (fixed effect, two levels), number of individuals treated for TB in the pre-randomization period, and patient-level covariates (age, sex, and HIV status for the overall rate ratio). Individuals with unknown HIV status were excluded (855 with unknown HIV status for the mortality rate outcome [199 in the intervention arm and 656 in the routine care arm], 7 deaths/164.0 person-years in the intervention arm and 19 deaths/539.2 person-years in the routine care arm). The p-value for the overall adjusted rate ratio = 0.29.

When adjusting for age, sex, HIV status, and cluster-level covariates (randomization strata and number of individuals treated for TB in the pre-trial period), the mortality rate ratio was 0.77 (95% CI: 0.47–1.28, p = 0.29), favoring the intervention. The mortality rate ratio was similar when including those with unknown HIV status and adjusting for only age and sex at the patient level (aRR 0.80, 95% CI: 0.48–1.34, p = 0.38, Table S8).

Subgroup analysis by HIV status showed some evidence for interaction by trial arm (p = 0.04). Among people living without HIV, the mortality rate was lower in the intervention arm than in the routine care arm (aRR 0.50, 95% CI: 0.26–0.96). Conversely, the mortality rate was slightly higher in the intervention arm than the routine care arm for the subgroup of PLHIV, although confidence intervals were wide (aRR 1.10, 95% CI: 0.65–1.86). The mortality rate ratio for females favored the intervention, but the confidence intervals were also wide (aRR 0.64, 95% CI: 0.33–1.23; p-value for interaction = 0.19) (Table 2).

Results of the rate difference analyses and additional sensitivity analyses are available in the Supplement and yielded similar results (Table S7; Table S8).

### Six-month mortality risk outcome

The overall unadjusted six-month mortality risk in the intervention arm was 2.5% (116 deaths observed among 4421 individuals), compared to 3.1% (123 deaths observed among 3695 individuals) in the routine care arm (Table S5). Similar to the findings for the mortality rate outcome, the unadjusted mortality risk was higher among PLHIV than people living without HIV (intervention: 3.8% vs. 1.5%, routine care: 3.9% vs. 2.7%) and among males vs. females (intervention: 3.5% vs. 1.5%, routine care: 3.6% vs. 2.8%).

When adjusting for age, sex, HIV status, and cluster-level covariates, the mortality risk ratio was 0.77 (95% CI: 0.44–1.35, p = 0.34), favoring the intervention. The mortality risk ratios for subgroup analyses were similar to the rate outcome, with lower mortality for people living without HIV and females in the intervention vs. routine care arm, though no evidence for interaction was observed (Table S5).

## Discussion

Overall, we observed low mortality in this cohort of adults being evaluated for TB at 20 community health centers in Uganda and did not detect a significant difference in mortality between the intervention and routine care arms. However, the point estimate (1% absolute and 23% relative reduction in adjusted all-cause mortality) would represent an important reduction in mortality at the population level, if confirmed in other settings or when analyzed in combination with other studies. The intervention effect was most pronounced among the subgroup of people living without HIV, where we saw a 50% reduction in mortality. The small absolute effect size observed in this very large trial suggests that any single study assessing the impact of novel TB diagnostics among ambulatory populations is likely to be underpowered to detect a mortality difference. While mortality remains important to measure, more proximal outcomes with public health impact such as diagnostic yield and pre-treatment loss to follow-up should drive policymaking and scale-up considerations.

The results from this study, a secondary outcome of the XPEL-TB trial, are consistent with pooled analyses of previous trials comparing centralized or decentralized molecular testing for TB to sputum smear microscopy that assessed mortality as an outcome. A large individual patient data meta-analysis that included 8142 individuals in South Africa, Zimbabwe, Zambia, and Tanzania showed a similar six-month mortality risk reduction (pooled effect size 0.88 [95% CI: 0.68–1.14]) with Xpert testing in comparison to sputum smear microscopy alone.^16^ A more recent meta-analysis that reviewed additional studies also showed a similar overall risk reduction (pooled effect size 0.89 [95% CI 0.75–1.05]).^17^ Although our study and these meta-analyses on their own do not provide conclusive evidence of an effect on mortality, the consistent point estimates favoring a mortality reduction across studies suggest a likely population-level impact of molecular testing.

Interestingly, we found that the XPEL-TB intervention appeared to have an effect on mortality among people living without HIV. In contrast to PLHIV, people living without HIV may not have been engaged in care prior to their episode of TB, may have taken longer to diagnose, and may not have had other co-morbidities or risk factors for death.^24^ Thus, TB may have been an important contributor to death among those living without HIV, and therefore early detection of TB may have had a greater impact. It is also possible that we saw improved care overall at the intervention clinics due to the quality improvement measures in place.

While we ultimately aim to prevent mortality through improved TB diagnosis, the results of our trial—the largest study to assess the impact of a rapid molecular diagnostic for TB—along with the results of previous trials suggest that diagnostic trials among non-hospitalized populations are unlikely to be able to demonstrate mortality reductions. Overall mortality is low among adults attending outpatient health facilities and the sample size required for reasonable power to assess even moderately large effect sizes is difficult to achieve within funding constraints. Notably, there are other benefits of rapid diagnosis and treatment initiation including reductions in morbidity and secondary infections and improvements in quality of life. Trials and guideline development should focus on person-centered outcomes to establish the benefit of one diagnostic approach over another including cost and time to attain a diagnosis and be linked to treatment, loss of productivity, and satisfaction with care, as well as cost-effectiveness.^25^

Our study had limitations that should be considered. While the study team achieved high follow-up rates, vital status was unknown for 12% of the patient population and ascertainment of vital status differed slightly by trial arm and by health center. In addition, we could only adjust for a small number of covariates due to limited data available in TB registers. It is possible there were differences in unmeasured covariates by trial arm, including comorbidities and disease severity. HIV status was defined based on the TB registers and missing for some. Misclassification of those with a negative status is possible but unlikely since HIV testing was part of routine care and incorporated into the guidelines for presumptive TB patients in Uganda.^26^ Although our exclusion of those with unknown HIV status could introduce bias if the data were not missing at random, sensitivity analyses that included patients with unknown HIV status yielded similar results for both adjusted rate and risk difference analyses.

In summary, while the multicomponent intervention strategy, including decentralized Xpert testing, increased the number of individuals being diagnosed with and treated for TB, we did not observe a significant reduction in mortality in this population with low mortality. Consistent with previous studies, the direction of the effect indicated by the point estimates favored the intervention strategy, suggesting a small absolute risk reduction in mortality but potentially moderate to large relative risk reduction. Future trials of novel diagnostics should primarily focus on improvements in TB case detection and treatment initiation, as well as other person-centered outcomes.

## Contributors

AK, AC and KF conceptualized and designed the study and acquired funding with input from ST, MJ, DWD, DAJM, JLD, PS.

TN, MN, DO, SN, DB, JM, AN, IN, KA, TR conducted and coordinated the research activity.

AC, KF, DWD, DAJM, JLD and PS provided scientific support.

TM and KF did the formal data analysis.

AK, TM and AC wrote the manuscript draft.

KF, DWD, DAJM had substantial input on the manuscript.

All authors had full access to all data in the study and contributed to the interpretation of data, reviewed and edited the manuscript, approved the final version of the manuscript, and had final responsibility for the decision to submit for publication.

TM and KF had access to and verified all the data.

## Data sharing statement

Complete de-identified participant data will be made available upon request. Statistical analysis plan will be available with publication and can be obtained by e-mailing the corresponding author.

## Declaration of interests

KF declares salary funding paid to her institution from the National Institutes of Health (NIH). AC declares a grant to his institution from the NIH. All other authors declare no competing interests.

## Appendix A Supplementary data

Nejmoa2105470_protocol
