# Supplementary material for: Impact of a multicomponent strategy including decentralized molecular testing for tuberculosis on mortality: planned analysis of a cluster-randomized trial in Uganda
Source: eClinicalMedicine. 2024 Nov 26;78:102953. doi: 10.1016/j.eclinm.2024.102953 (PMC11629260; doi:10.1016/j.eclinm.2024.102953)
Supplement: Nejmoa2105470_protocol [file mmc1.pdf]

Protocol

Protocol for: Cattamanchi A, Reza TF, Nalugwa T, et al. Multicomponent strategy with decentralized molecular testing for tuberculosis. N Engl J Med 2021;385:2441-50. DOI: 10.1056/NEJMoa2105470

This trial protocol has been provided by the authors to give readers additional information about the work.

This supplement contains the following items:

1. Original protocol, final protocol, summary of changes.
2. Original statistical analysis plan, final statistical analysis plan, summary of changes.

GeneXpert Performance Evaluation for Linkage to Tuberculosis Care:
The XPEL TB Trial

ClinicalTrials.gov Identifier:
NCT03044158

Pan African Clinical Trials Registry:
PACTR201610001763265

Sponsored by:
NIH/NHLBI

Unique Protocol ID:
R01HL130192

Principal Investigator:
Adithya Cattamanchi MD, MAS

Draft or Version Number:
Version 1.0

Day Month Year
May 1, 2018

Statement of Compliance

The study will be carried out in accordance with Good Clinical Practice (GCP) as required by the following:

- *U.S. Code of Federal Regulations applicable to clinical studies (45 CFR 46)*
- *ICH GCP E6*
- *Completion of Human Subjects Protection Training*
- *NIH Clinical Terms of Award*

Refer to: http://www.hhs.gov/ohrp/humansubjects/guidance/45cfr46.htm#46
http://www.fda.gov/cder/guidance/959fnl.pdf
http://grants.nih.gov/grants/guide/notice-files/NOT-OD-01-061.html
http://cme.cancer.gov/c01/

Signature Page

The signature below constitutes the approval of this protocol and the attachments, and provides the necessary assurances that this trial will be conducted according to all stipulations of the protocol, including all statements regarding confidentiality, and according to local legal and regulatory requirements and applicable US federal regulations and ICH guidelines.

Uganda Study Site Principal Investigator:*

Signed:_________________________________ Date:______________________
Achilles Katamba, MBChB, PhD

Overall Study Principal Investigator:*

Signed:_________________________________ Date:______________________
Adithya Cattamanchi, MD, MAS

Table of Contents

Overview 6
Key Roles 7
Background 8
Background information 8
Theoretical basis for intervention strategy 9
Table 2. Key clinic-level barriers targeted by intervention strategy. 11
Objectives 12
Methods/Design 13
Overview 13
Target Setting and Study Population 13
Eligibility criteria for the cluster-randomized trial 13
Eligibility criteria for mixed methods and health economic sub-studies 14
Recruitment 14
Health centers 14
Participants for cluster-randomized trial 15
Participants for mixed methods and health economic sub-studies 15
Intervention and Control (Wait-list) Arms 15
Figure 2. Comparison of TB diagnostic evaluation in the intervention and control arms 16
On-Site Molecular Testing 16
Process Redesign for Same-Day TB Diagnosis and Treatment 17
Performance Feedback 17
Randomization 18
Procedures 18
Health center training 18
Patient-level data collection 19
Patient surveys 19
Provider surveys 20
Health system costing 20
Focus group discussions 20
Patient vital status assessment 20
Provider interviews 21
Figure 3. Site visit activities 21
Outcomes 22
Definitions 22
I. Primary Outcome (Effectiveness) 23
II. Secondary Outcomes (Effectiveness) 23
III. Implementation Outcomes 24
IV. Cost-effectiveness/Modeling Outcomes 25
Data Management 26
Statistical Analysis 26
Aim 1 26
Aim 2 27
Aim 3 27
Sample Size Considerations 28
Aim 1 28
Aim 2 29
Aim 3 29
Ethical Considerations 30

Potential risks to participants 30
Protection against risk 30
Data protection 31
Potential benefits of the study to participants and society 31
Dissemination 31
Trial governance 31
Abbreviations 32
Competing interests 33
Trial Steering Committee 33
References 34

Overview

**Title**	GeneXpert Performance Evaluation for Linkage to Tuberculosis Care: The XPEL TB Trial
**Target Population**	Adults undergoing evaluation for pulmonary TB (N=6500-7500)
**Sites**	20 TB microscopy centers (10 intervention, 10 control) in Uganda
**Study Design**	Clustered-randomized trial with nested mixed methods and economic/transmission modeling studies
**Study Duration**	2 years
**Objectives**	**Aim 1: To compare patient outcomes at health centers randomized to intervention vs. standard-of-care TB diagnostic evaluation strategies.**<br>• *Intervention:* onsite molecular testing for TB + process redesign to facilitate same-day TB diagnosis and treatment + performance feedback<br>• *Standard-of-care*: onsite ZN or LED fluorescence microscopy + hub-based GeneXpert testing per existing protocols<br><br>**Aim 2: To identify processes and contextual factors that influence the effectiveness and fidelity of the intervention TB diagnostic evaluation strategy.**<br><br>**Aim 3: To compare the costs and epidemiological impact of intervention vs. standard-of-care TB diagnostic evaluation strategies.**

Key Roles

Principal Investigator

Adithya Cattamanchi, MD, MAS
Associate Professor of Medicine, University of California San Francisco
San Francisco General Hospital
1001 Potrero Ave, Room 5K1
San Francisco, CA 94110
Phone: +1-415-206-5489
Fax: +1-415-695-1551
adithya.cattamanchi@ucsf.edu

Uganda Principal Investigator

Achilles Katamba, MBChB, MSc, PhD
Senior Lecturer, Makerere University College of Health Sciences
Upper Mulago Hill Road
Kampala, Uganda
Phone: +256-(0)414-530-692
Fax: +256-(0)414-540-524
akatamba@yahoo.com axk95@case.edu

NHLBI Program Officer

Elisabet Caler, PhD
AIDS Program Lung Team Leader
Lis.caler@nih.gov

Background

Background information

Prompt diagnosis and treatment of TB patients is essential to making progress towards TB elimination. However, at least 4.3 million of the estimated 10.4 million new cases in 2015 were not detected and reported to the World Health Organization (**WHO**).[1] There are three overarching reasons for this large gap: TB patients are not being notified to public health authorities, not seeking care, or not being diagnosed and treated even after seeking care. The last reason represents a clear health system failure that is pervasive in high burden countries – a recent systematic review of published studies found that up to 38% of sputum smear-positive patients in Africa and 28% in Asia are lost to follow-up prior to treatment initiation.[2] Patients with smear-negative TB are even less likely to complete the diagnostic cascade of care and be linked to treatment.

A principal reason for these failures in linkage to care is the inadequacy of the current approach to TB diagnosis at community health centers. It is well known that sputum smear microscopy, the most common test for TB at community health centers worldwide, has important limitations that contribute to delays in TB diagnosis and treatment. First, smear microcopy has sub-optimal sensitivity, identifying only about 50% of patients who actually have TB.[3] Second, the typical process of sputum collection and smear examination is burdensome for patients and staff. Guidelines usually require staff to ask patients to submit a sputum specimen on the day of presentation, return the following morning to submit a second specimen, and return a third time for treatment initiation (if smear-positive) or to consider further workup (if smear-negative). In high burden countries, clinicians fail to refer up to half of patients reporting TB symptoms for sputum smear examination.[4, 5] In addition, the direct and indirect costs of this standard multi-day diagnostic evaluation process consume up to 3 months of household income for already poor patients.[6-10] It is therefore not surprising that a substantial proportion do not return after their initial health center visit to submit additional sputum specimens, collect results or initiate treatment if smear-positive.[5, 11-13]

To address these limitations, there has been substantial donor investment in scale-up of Xpert MTB/RIF (**Xpert**)[14], a semi-automated molecular assay endorsed by the WHO in 2010 and by the US FDA in 2013. Xpert identifies 90% of TB cases, has a 2-hour turn-around time, and can be performed with minimal training and human resource requirements.[15] Although this represents an important advance, modeling analyses have found that Xpert is unlikely to bend the TB incidence curve significantly, primarily because it cannot be deployed at community health centers in high burden countries.[16] Indeed, because of high device costs and infrastructure requirements, the vast majority of Xpert devices are being placed at district or higher-level facilities[14], which are accessed by <15% of the population.[17] Many countries have therefore adopted a hub-and-spoke model in which several community health centers (spokes) are linked to an Xpert testing site (hub). However, a cluster-randomized trial has found that Xpert implementation using a hub-and-spoke model did not impact mortality; the major reason was failure to link patients with confirmed TB to treatment.[18]

The next generation of molecular diagnostics has strong potential to close gaps in the TB diagnostic cascade of care. In particular, GeneXpert Omni is a portable, single-cartridge version of the GeneXpert platform that is already WHO- and FDA-approved, and widely used in Uganda and other high TB burden countries. Because it only has a single test module (instead of 4 or more), it is lower cost (subsidized price $5000 vs. $10000 or more for previous GeneXpert platforms) and has lower power requirements enabling it to be battery-operated.[19] These features increase the likelihood that onsite Xpert testing can eventually be scaled up as a replacement for sputum smear at community health centers.

Our *overall objective* is to assess the effectiveness, implementation and costs of a streamlined TB diagnostic evaluation strategy based around rapid, onsite molecular testing. The intervention strategy was developed based on theory-informed assessment of barriers to TB diagnostic evaluation at community health centers in Uganda and a process of engagement with local stakeholders. It includes: 1) Point-of-care molecular testing using GeneXpert as a replacement for sputum smear microscopy; 2) Re-structuring of clinic-level procedures to facilitate same-day TB diagnosis and treatment; and 3) Quarterly feedback of TB evaluation metrics to health center staff. Our central hypothesis is that the intervention strategy will have high uptake and increase the number of patients diagnosed with and treated for active pulmonary TB. To test this hypothesis, we will conduct a *pragmatic cluster-randomized trial* at community health centers that provide TB microscopy services in Uganda in partnership with the National TB Program (**NTP**). We utilize an *effectiveness-implementation hybrid design* in which, concurrent with the clinical trial, we will conduct nested mixed methods, health economic and modeling studies to assess 1) whether the intervention strategy modifies targeted barriers to TB diagnostic evaluation; 2) fidelity of implementation of the intervention components (*i.e,* the degree to which intervention components were implemented as intended vs. adapted across sites); and 3) cost-effectiveness and public health impact. We will use the RE-AIM framework to evaluate how the intervention strategy affects processes and outcomes important to patients and TB programs, and to determine its scale-up potential. RE-AIM encompasses 5 dimensions common to successful multi-level interventions: depth of reach into a target population; effectiveness; factors that promote adoption; resources needed for implementation; and factors that ensure maintenance over time.[20] Thus, our focus is on evaluating the impact of the proposed multi-level intervention, rather than evaluating the impact of its individual components. The process of guideline implementation and technology uptake is complex, and it is unlikely that desired outcomes can be achieved with a simple intervention. However, if the desired outcomes are achieved, the proposed mixed methods studies will identify which barriers were modified, providing some insight into which intervention components are critical. In addition, future studies can focus on testing simplified versions of the multi-level intervention that remove one or more components.

Theoretical basis for intervention strategy

The intervention strategy was designed based on a mixed methods study conducted at six health centers in Uganda to better understand reasons for gaps along the TB diagnosis cascade of care and to inform intervention design. The process was guided by the Theory of Planned

Figure 1. Theory-informed barrier assessment and intervention design

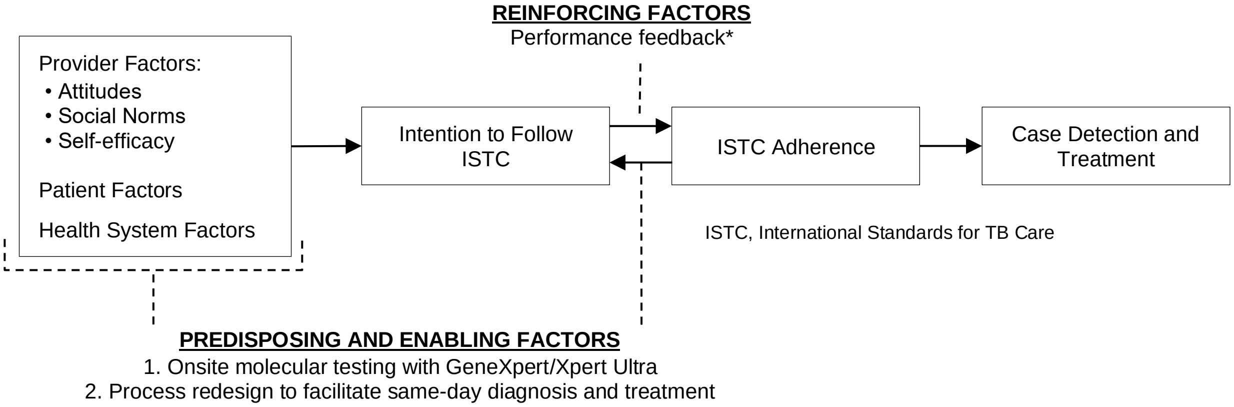

Behavior as the conceptual framework and the PRECEDE model to select intervention components (**Figure 1**). In a systematic review of guideline implementation studies, the Theory of Planned Behavior was the most likely theory to predict guideline adherence.[21] This theory asserts that intention is the best predictor of behavior and that three factors mediate the strength of intention: 1) *attitudes* – expected value of behavioral performance; 2) *subjective norms* – what others think about the behavior; and 3) *self-efficacy* – perception of ability to overcome barriers to behavioral performance.[22] The PRECEDE model was chosen based on its strong empirical base and applicability to guideline adherence. The model is based on three factors relevant to health behavior change: 1) *predisposing* factors – prior motives that either support or inhibit behavior; 2) *reinforcing* factors – rewards or punishments following a behavior or anticipated as a consequence of it; and 3) *enabling* factors – objective characteristics of an individual or environment that facilitate behavior.[23] A meta-analysis of 50 randomized controlled trials of continuing medical education demonstrated that the studies employing a combination of interventions representing PRECEDE categories were the most likely to influence patient outcomes.[24]

The intervention strategy was designed in consultation with the Uganda NTP to target modifiable clinic-level barriers identified in the formative assessment as well as key theoretical constructs. Key barriers to TB evaluation identified through semi-structured interviews with 22 staff at the six health centers and organized within PRECEDE model categories are shown in **Table 2**.[25] The intervention strategy includes: 1) onsite molecular testing to reduce laboratory workload and increase the sensitivity of TB diagnostic testing; 2) re-structuring clinic-level procedures to facilitate same-day TB diagnosis and treatment, thereby increasing provider self-efficacy/motivation and enabling linkage of confirmed TB patients to treatment; and 3) performance feedback to increase communication between staff and with NTP supervisors, thereby reinforcing uptake of onsite molecular testing and adherence to TB evaluation guidelines. Re-assessing these barriers as part of Aim 2 will help understand whether the XPEL TB intervention worked as intended and help explain why it was or was not effective in improving TB diagnostic evaluation outcomes.

Table 2. Key clinic-level barriers targeted by intervention strategy.

**PRECEDE framework**	**Recurring themes highlighting barriers targeted by the intervention strategy**
**Predisposing factors**<br>(Knowledge, attitudes, beliefs, intention)	• Time and resource constraints → low self-efficacy<br>• Low motivation of staff<br>• Low sensitivity of sputum smear microscopy<br>• Poor patient perception of care at government health centers
**Enabling Factors**<br>(Factors that if addressed make it easier to initiate the desired behavior)	• Failure of patients to return after initial visit (due to time and costs)<br>• Inability to track and follow-up patients → low-self-efficacy<br>*"When they have a cough for more than 2 weeks they are sent to the lab. But the problem is they get the first sample and sometimes, actually most times they don't bring the second sample."*
**Reinforcing Factors**<br>(Factors that if addressed make it easier to continue the desired behavior)	• Lack of communication and coordination among staff<br>• Insufficient oversight from NTP<br>*"…Actually at times we have met but we don't meet [regularly], only when we realize there is a problem that's when we communicate and say why is this happening, then we try to rectify."*

Objectives

Aim 1: To compare patient outcomes at health centers randomized to intervention vs. standard-of-care TB diagnostic evaluation strategies. We will randomize 20 community health centers to continue standard TB evaluation (routine microscopy plus referral of patients for Xpert testing per existing processes of care) or to implement the intervention strategy (1. Onsite molecular testing; 2. Re-structuring clinic-level procedures to facilitate same-day TB diagnosis and treatment; and 3. Performance feedback). We will compare reach and effectiveness based on the numbers and proportions of patients (N=6500) who complete TB testing, are found to have TB, and have treatment initiated within one week of specimen provision.

Aim 2: To identify processes and contextual factors that influence the effectiveness and fidelity of the intervention TB diagnostic evaluation strategy. We will use quantitative process metrics to assess the adoption and maintenance over time of the core components of the intervention strategy. We will also collect quantitative and qualitative data to describe the fidelity of implementation of each component and faithfulness to our conceptual model.

Aim 3: To compare the costs and epidemiological impact of intervention vs. standard-of-care TB diagnostic evaluation strategies We will model the incremental costs and cost-effectiveness of intervention relative to standard-of-care TB diagnostic evaluation from the health system and patient perspective. We will then construct an epidemic model of the population-level impact of the intervention strategy on TB incidence and mortality.

Methods/Design

Overview

The study proposes to conduct a pragmatic, parallel cluster-randomized trial with nested mixed methods, health economic and modeling studies to evaluate the effectiveness, implementation and costs of the intervention strategy relative to standard TB diagnostic evaluation at community health centers that are part of Xpert referral networks in Uganda. The effectiveness of the intervention strategy will be assessed using routine data collected as part of mandatory reporting to the Uganda NTLP on consecutive patients who present to participating health centers during the 2-year trial period and meet eligibility criteria. Selected patients and providers who provide informed consent will also be surveyed, interviewed, and/or participate in focus groups to identify reasons for its success or failure across study sites (Aim 2), and to collect relevant cost data for cost-effectiveness analyses (Aim 3).

Target Setting and Study Population

The target setting is community health centers with TB microscopy units (*i.e.,* the lowest level of the health system where TB diagnostic services are provided by the Uganda NTLP). The unit of randomization will be community health centers (N=20). The unit of analysis will be patients undergoing TB diagnostic evaluation during the 2-year trial period (estimated total 6500 over 2-year trial period).

Eligibility criteria for the cluster-randomized trial

A. *Site-level Inclusion Criteria*
    1. Use standard (multi-day) sputum smear microscopy as the primary method of TB diagnosis
    2. Participate in NTP-sponsored external quality assurance (**EQA**) for sputum smear microscopy
    3. Send samples to a district or regional hospital/health center for Xpert testing
B. *Site-level Exclusion Criteria*
    1. Do not agree to be randomized to standard-of-care vs. intervention arms
    2. Perform sputum smear examination on <150 patients per year (based on 2015 data)
    3. Diagnose <15 smear-positive TB cases per year (based on 2015 data)
C. *Patient-level Inclusion Criteria*
    1. Initiate evaluation for active pulmonary TB at a study health center
D. *Patient-level Exclusion Criteria*
    1. Have sputum collected for monitoring of response to anti-TB therapy
    2. Have sputum collected as part of active, community-based case finding (e.g., contact tracing, community outreach campaign)
    3. Referred to a study health center for TB treatment after a diagnosis is established elsewhere
    4. Started on TB treatment for extra-pulmonary TB only

In addition, patients age <18 years and those with a documented prior history of TB treatment (e.g., reason for Xpert testing or TB treatment marked as treatment failure, relapse, treatment after loss to follow-up, etc.) will be excluded from the primary analysis of all outcomes. Patients identified as RIF resistant by Xpert testing will be excluded from the primary analysis of all outcomes except for the comparison of the number and proportion of patients with RIF resistance between arms.

Eligibility criteria for mixed methods and health economic sub-studies

Patient Surveys: Same eligibility criteria as for the CRT.

Provider Survey: Providers at each study site who are (a) aged ≥18 years; (b) employed by the peripheral health center; and (c) involved in the conduct or supervision of health center work related to diagnosis and management of TB will be included.

Focus Group Discussion and Interviews: Same inclusion criteria as for the provider survey.

Recruitment

Health centers

Potential trial sites were identified from a list of Uganda NTLP-affiliated TB microscopy centers that refer samples to Xpert testing hubs. Study staff reviewed 2014 TB testing and treatment data reported to the Uganda NTLP to identify health centers that meet eligibility criteria (based on numbers of patients tested for TB and found to be smear-positive), focusing on those within 150 km of Kampala for study feasibility purposes. Study staff then obtained permission from District Health Officials and Health Center Directors for visiting health centers to confirm eligibility and assess interest in study participation. At the site assessment visit, study staff counted from the 2015 TB laboratory register the number of patients tested by microscopy and the number of smear-positive patients (excluding patients tested for treatment monitoring or as part of community-based active case finding activities). Health centers confirmed to be eligible and that expressed interest in participating in the study were reviewed with Uganda NTLP and NTRL Directors. Following their approval, research staff met with the District Health Officer (**DHO**) to inform him or her about the project and requested the participation of potential trial sites in the District. The DHO was asked to sign a Memorandum of Understanding (**MoU**) that describes key study procedures and expectations of participating sites. If the DHO signed the MoU, study staff scheduled an enrollment visit with potential trial sites in the District by calling the facility 3-4 days prior to the anticipated visit date. Project staff then contacted the Program Manager of the Uganda NTLP to arrange for an NTLP representative to participate in the enrollment site visit. During the site visit, project staff followed a standard script to present the project to health center staff, outlined the project aims, and described project procedures. Project staff presented a copy of the MoU signed by the DHO and endorsed by the Chief Administrative Officer and NTLP Program Manager to the health center in-charge. Of note, the final list of 20 sites to be randomized was determined in consultation with the trial statistician (to minimize potential for site-level variation that could impact power).

Participants for cluster-randomized trial

This is a pragmatic trial that will study outcomes of routine care. Thus, rather than recruiting participants, the intervention and control arms will be compared using data available in routine TB laboratory and treatment registers for consecutive patients who present to participating health centers during the two-year trial period and meet eligibility criteria (estimated N=6500 patients).

Participants for mixed methods and health economic sub-studies

Patient Cost and Satisfaction Surveys: The patient cost and satisfaction surveys will be administered during the baseline (pre-randomization) period (N=20/site; 400 total) and in the second year after randomization (N=40/site, 800 total). Health center staff will assist with identification and recruitment of patients who are interested in participating in the surveys (from among patients providing sputum samples or receiving results). Participation will be voluntary. Research staff will then contact interested patients by phone at the end of their health center visit to review the verbal consent script, answer questions the patient may have, and administer the survey to consenting patients.

Provider Survey: The provider survey will be administered during the baseline (pre-randomization) period and in the second year after randomization. All eligible providers at each study site (N=5-10/site; 100-200 total) will be invited to participate in the survey. Participation will be voluntary and written informed consent will be obtained.

Provider Focus Group Discussions and Interviews: Focus group discussions will occur during the second year after randomization and semi-structured interviews after the trial is completed. All eligible providers at intervention sites (N=5-10/site; 50-100 total) will be invited to participate in focus group discussions. Providers (N=20-30) at low- and high-performing sites will be invited to participate in semi-structured interviews. Participation in focus group discussions and interviews will be voluntary and written informed consent will be obtained.

Intervention and Control (Wait-list) Arms

With the current standard-of-care (*i.e.*, control arm), patients make up to 4 health center visits to complete TB diagnostic evaluation (**Figure 2**). At the first visit, sputum is collected for smear microscopy. All patients are required to return for a second visit; patients are initiated on TB treatment if the first sputum is smear-positive or are asked to provide a second sputum specimen and return for a third visit to collect results if the first sputum is smear-negative. Patients who return for a third visit are initiated on TB treatment if the second sputum is smear-positive or are asked to provide a third sputum specimen that is sent out for Xpert testing. Patients are asked to return for a fourth visit to collect Xpert results. Patients who return for a fourth visit are initiated on TB treatment if Xpert results are positive (or referred to an MDR TB treatment center if Xpert testing indicates rifampin resistance). Of note, HIV-infected patients have sputum collected for Xpert testing at their first visit and all patients could be started on TB treatment empirically at any visit.

The intervention strategy seeks to facilitate diagnosis and treatment of TB at the initial health center visit (or referral of patients to MDR TB treatment center if rifampin resistance is detected

by Xpert). As further described below, it includes the following components: 1) onsite molecular testing as a replacement for microscopy; 2) process re-design; and 3) feedback of TB diagnostic evaluation quality indicators to health center staff.

Figure 2. Comparison of TB diagnostic evaluation in the intervention and control arms

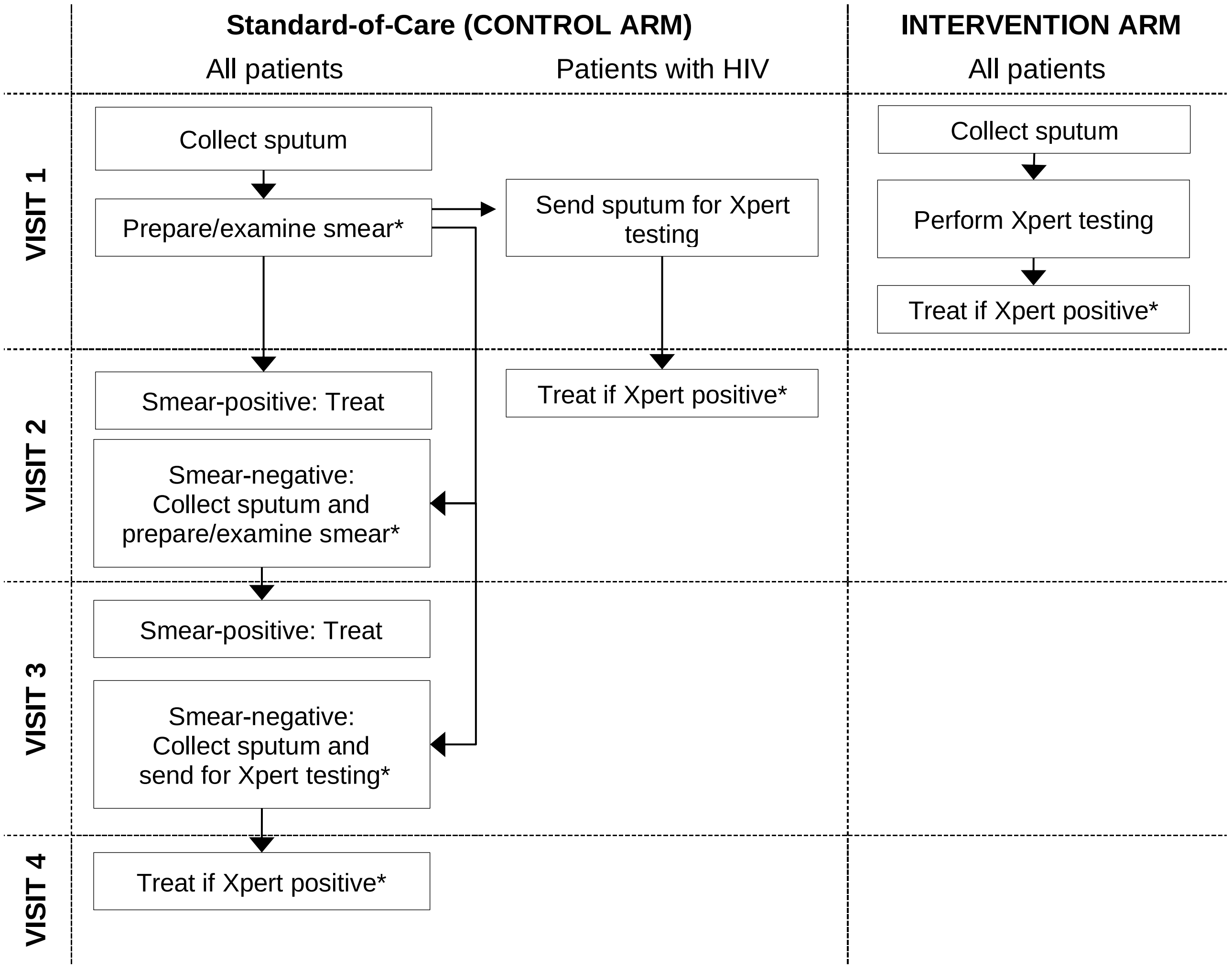

*Consider empiric treatment or additional testing if test results are negative or invalid

On-Site Molecular Testing

GeneXpert Omni (Cepheid, Sunnyvale, CA) is a point-of-care molecular diagnostic system designed for lower level health facilities. It is small (1.0 kg weight), portable, and consumes less power than previous GeneXpert systems. The standard configuration includes a single test cartridge module, a mobile device, a country-specific AC/DC power cord, a supplemental battery, and a user guide. The rechargeable battery allows for up to 4 hours of operation with voltage surge protection for unexpected power supply interruptions. Additionally, a supplemental battery can be charged for up to 12 hours of use in the event of sustained power loss. Data is stored in real-time via cloud-based connectivity to Cepheid Cloud Control using Wi-Fi or cellular networks. Training materials will be provided by Cepheid, with an anticipated training time of

one half-day. Additional device-integrated videos and self-instructions are available for training reinforcement.[19]

Due to delays in release of GeneXpert Omni, the trial will begin with intervention sites using a modified conventional GeneXpert instrument (GeneXpert I). GeneXpert I is a one-module device that includes a 4-hour battery back and a specially-fitted dust cover. The device can be operated by a touchscreen tablet instead of a laptop computer. Sites randomized to the intervention arm will have a solar panel installed temporarily to power the GeneXpert I device. Once GeneXpert Omni is available (expected in early 2019), it will replace the GeneXpert I instrument, and the solar power will be removed. After the trial is completed, a GeneXpert Omni or GeneXpert I device will be provided to control health centers and health center staff will be trained on the use and maintenance of the device.

Of note, Xpert testing in both the control and intervention arms will occur using the same cartridge, Xpert MTB/RIF or Xpert MTB/RIF Ultra, depending on which ever is in use in Uganda at the time of the trial.

Process Redesign for Same-Day TB Diagnosis and Treatment

Research and Uganda NTLP staff will engage health center staff in a discussion of how to re-organize clinical, laboratory and pharmacy services to enable same-day TB diagnosis and treatment. Health center staff will be asked to identify site-specific solutions to:

1. Rapid screening for TB at registration desk and/or waiting area
2. Immediate referral of patients identified during screening as needing further testing to the lab for sputum collection
3. On-demand testing of sputum specimens and immediate reporting of results to clinicians and patients
4. Availability of TB pharmacy services throughout the clinic day

Agreed upon changes to existing procedures and staff responsibilities will be recorded and reviewed with the health center in-charge, lab director and TB pharmacist.

Performance Feedback

Performance feedback is a strategy employing regular monitoring and feedback to allow health care workers to critically analyze performance and identify areas for improvement. A systematic review of audit and feedback interventions identified 5 factors associated with greater impact: low baseline performance, feedback coming from a supervisor or colleague, feedback provided multiple times, feedback delivered in both verbal and written form, and feedback including explicit targets and an action plan.26 Thus, performance feedback will involve delivery of a monthly Report Card which displays 1) TB diagnostic evaluation quality indicators for the current month and for the previous 6 months; 2) performance data averaged across all intervention health centers; and 3) performance data for the top- performing intervention health center. The Report Card will include a section that asks staff to write down their interpretation of the performance data, identify barriers to performance improvement, and specify plans to improve performance. The Report Card will be introduced to health center staff at the post-randomization site visit and will then be sent electronically every month to the health center in- charge or TB

focal person. Health workers will be asked to review the Report Card at monthly staff meetings to devise a performance improvement plan. This process will continue monthly, with each new Report Card being used to evaluate the success of plans developed in the previous month and determine the need for new actions. Report Cards will be collected by study staff at quarterly site visits to assess use and completion.

Randomization

Health centers will be grouped into two strata using baseline data for the primary outcome (see Outcomes below), and randomized within strata to help reduce between-cluster variation and improve balance between arms at baseline.[26] Restriction, a common approach in cluster-randomized trials with a small number of clusters, may be used to help achieve baseline balance of important site- and patient-level covariates.[27] We will consider restricting on factors likely to be associated with the primary outcome including health center region (four quadrants of Uganda), health center size (based on volume of patients tested for TB), HIV prevalence among TB patients, and patient cost and satisfaction with care. The restriction factor will be calculated and the validity of the restriction assessed using the validity matrix.[28] The Trial Statistician (KF) will be responsible for the randomization. Randomization will be unveiled to health center directors (or their designated representative) at a meeting chaired by the Uganda NTLP Director.

Procedures

The following procedures will occur at site visits to each participating health center. Site visits will occur approximately every 2-4 months throughout the study.

Health center training

Training will occur at the first site visit after health center enrollment and after health center randomization.

Following enrollment, we will conduct TB guideline and registry training at the first pre-randomization site visit. Project staff, an NTLP/NTRL representative and the District TB Officer will conduct the training jointly using a standardized slide set that emphasizes Uganda NTLP guidelines/algorithms for TB diagnosis and treatment. In addition, project staff will assess the completeness of data recording in TB registers, and provide training as needed to improve completeness. Last, project staff will visit all GeneXpert testing hubs that receive samples from project sites, and provide refresher training on GeneXpert device maintenance and operation.

Following randomization, we will conduct a 2-day site visit to each intervention and control health center.

At intervention health centers, we will engage staff in a discussion of the site-specific laboratory, clinical, and pharmacy workflow reorientation required as part of the intervention TB diagnostic evaluation strategy, with an emphasis on prompt identification of patients suspected of TB, immediate referral to the laboratory for sputum collection, on-demand molecular testing of sputum, communication of results to clinicians, and same-day treatment initiation. A GeneXpert I device and solar panel will be installed (with both replaced by GeneXpert Omni once it

becomes available in 2019), and laboratory staff will be trained on sputum collection, sputum testing using GeneXpert, recording of results, and device maintenance. Laboratory and clinical staff will be trained on interpretation of results. In addition, all staff will be introduced to performance feedback Report Cards and expectations for reviewing and acting on the Report Cards. A staff member will be identified to record any change made in the "Comments" section of the Report Card. Lab and drug inventories will be reviewed to ensure there is an adequate supply of sputum collection cups, glass slides, staining reagents and TB drugs. Completeness of TB laboratory and treatment registers will be assessed, and re-training provided as needed.

At control health centers, we will re-emphasize messages related to TB guidelines presented during the initial pre-trial site visit, including guidelines and procedures related to referral of sputum samples for Xpert testing. Laboratory staff will receive refresher training on sputum collection and sputum smear microscopy, including proficiency testing using panel slides. Lab and drug inventories will be reviewed to ensure there is an adequate supply of sputum collection cups, glass slides, staining reagents and TB drugs. Completeness of TB laboratory and treatment registers will be assessed, and re-training provided as needed.

Patient-level data collection

Patient demographic and clinical (HIV and TB testing and treatment) data will be collected at all sites. At the first pre-trial site visit, project staff will photograph each page of the following data sources from January 2016 until the visit date: 1) NTLP Presumptive TB, Laboratory and Treatment registers and 2) Xpert laboratory requisition forms. Project staff will also train two health center staff (one primary, one backup) identified by the health center in-charge to take photos of these data sources every two weeks for the duration of the project using a camera-enabled smartphone, and to upload the photos to a central secure server through REDCap mobile. Health center staff will be trained to delete photos from the phone after upload confirmation. In addition to these data sources, study staff will 1) Review pre-ART and ART registers during scheduled site visits to verify ART status for any HIV-positive patient missing ART information; 2) review additional data sources (*e.g.,* clinic registration logbook) as needed at scheduled site visits to attempt to track down key information missing from primary data sources; and 3) call MDR TB treatment centers to determine referral, additional testing and treatment status and outcomes for patients with RIF resistance identified through Xpert testing.

Patient surveys

We will administer cost and satisfaction with care surveys to 20 patients at each health center (N=400 total) in the pre-randomization period and to 40 patients at each health center (N=800) at least 6 months post-randomization. The two surveys will involve the same patients when feasible, but administered to different patients if a patient does not wish to complete both.

The cost survey will collect data on direct and indirect costs (time to complete visit, lost wages, etc.) of TB diagnostic evaluation. Cost data collection will be based on the Tool to Estimate Patient Costs developed by the TB Coalition for Technical Assistance, which we have already adapted and used in Uganda.

The satisfaction with care survey will assess 18 items taken from the previously validated Patient Satisfaction Questionnaire (**PSQ**)[29] and adapted to the Ugandan context.[30] The items

are constructed with a five-point Likert scale with categories ranging from “strongly disagree” to “strongly agree”, and include both positively and negatively worded questions to minimize the potential bias that occurs from clustering of responses to one side of the scale. The questionnaire measures general satisfaction as well as four dimensions of care known to impact satisfaction: 1) Accessibility, availability, and convenience of health services (3 items); 2) Provider interpersonal skills (5 items); 3) Provider technical competence with respect to patient education, examination and counseling (4 items); and 4) Health facility environment, specifically with respect to the cleanliness and space in the waiting area (2 items).

Provider surveys

We will administer a survey to all health workers involved in providing TB diagnostic and/or treatment services at each study site in the pre-randomization period and again at least 12 months after randomization. The survey will collect data on four key constructs of the Theory of Planned Behavior (**TPB**) as related to adherence to TB evaluation guidelines (**Figure 1**): intention, beliefs/attitude, normative beliefs/subjective norms and self-efficacy/perceived behavioral control. Based on prior experience, we anticipate 5-10 health workers will be involved in TB diagnostic/treatment services at each health center (N=100-200 health workers total pre- and post-randomization).

Health system costing

Health system cost data will be collected from all 20 sites at least 12 months after randomization. Cost data will be collected through detailed budgetary analysis, interviews of key staff members, review of logbooks and/or timesheets to record proportions of staff time devoted to various activities, and direct observation (*e.g*., time-motion studies of at least 10 patients and 3 staff members per clinic).

Focus group discussions

Study staff will conduct focus group discussions (one at each intervention health center) with all eligible providers at least 12 months after randomization. The focus group discussions will assess fidelity and barriers to uptake of each intervention component. Focus group discussions will be conducted by trained and experienced staff, audio-recorded, and professionally transcribed.

Patient vital status assessment

Study staff will call all participants included in the study 6 months after their initial health center visit to assess vital status. Staff will call phone numbers recorded in the lab register, Xpert referral form and/or TB treatment register. For participants who cannot be reached, staff will make up to two additional phone calls on successive days. If the participant or a family member cannot be reached by telephone, study staff will work with a community health worker from the health center at which the participant underwent TB testing to conduct a home visit (using the address recorded in the lab register, Xpert referral form and/or TB treatment register). In addition, staff will review TB treatment register data to ascertain vital status for participants who cannot be reached by phone but are known to have initiated TB treatment.

Provider interviews

Study staff will conduct in depth semi-structured interviews with 20-30 (2-3 per site) eligible providers to understand reasons for variability in uptake and effectiveness of the intervention strategy after completion of the trial. Health workers will be sampled purposively (*i.e.,* from low- and high-performing sites). Interviews will be conducted by trained and experienced staff, audio-recorded, and professionally transcribed.

Figure 3. Site visit activities

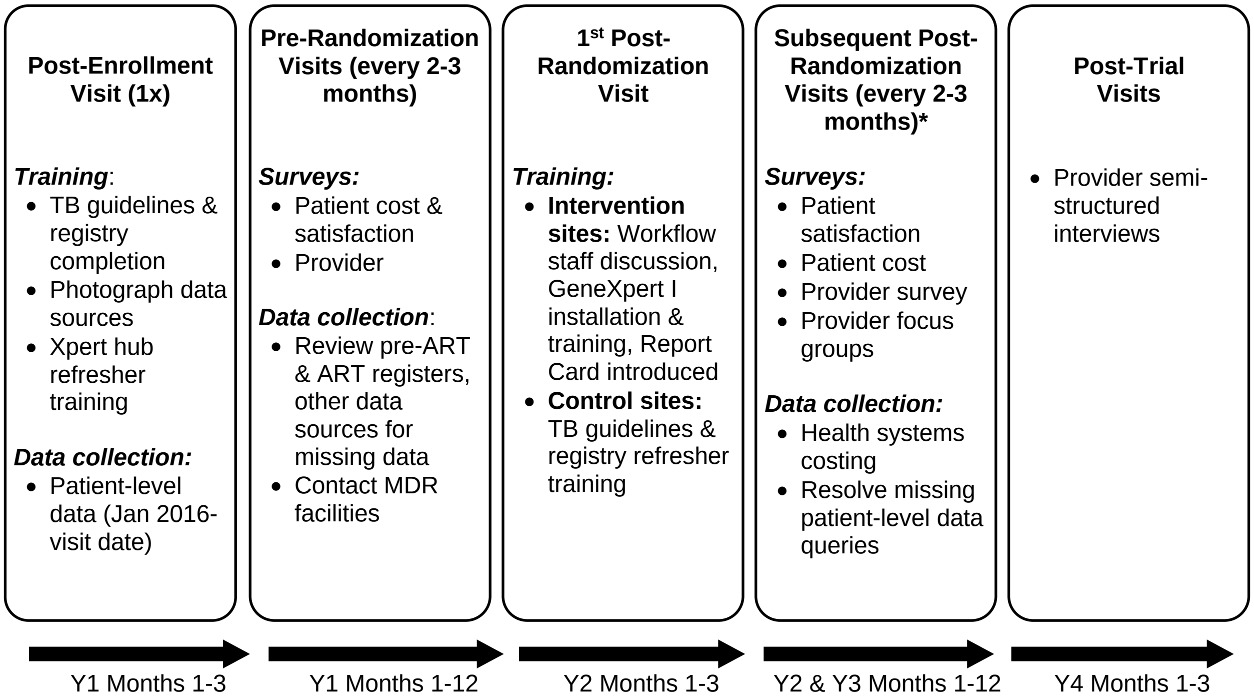

* At intervention heatlh centers, GeneXpert I will be replaced with GeneXpert Omni once Omni is available at a regularly scheduled post-randomization visit. Lab staff will receive training on use of GeneXpert Omni and the solar panel used to power the GeneXpert I instrument will be removed.

Outcomes

Definitions

- ***Number enrolled***: Number of eligible patients identified over a defined enrolment period through review of the NTLP Presumptive TB register, NTLP Laboratory register, GeneXpert laboratory requisition forms, GxAlert database and NTLP Treatment register at each study site (includes patients treated for TB without undergoing any sputum testing).

- ***Date enrolled***: Earliest date recorded in the NTLP Presumptive TB register, NTLP Laboratory register, GeneXpert laboratory requisition form, GxAlert database, or NTLP Treatment register. Records will be prospectively reviewed using name, sex, and age to identify patients presenting multiple times over the study period. Visits occurring within six months of the initial presentation will be considered as part of the same episode.

- ***Number referred for testing***: Number of eligible patients identified through review of the NTLP Presumptive TB register, NTLP Laboratory register, GeneXpert laboratory requisition forms, and GxAlert database at each study site.

- ***Number tested***: Number of eligible patients with any smear or Xpert result entered into the NTLP Presumptive TB register, NTLP Laboratory register, GeneXpert laboratory requisition forms, NTLP treatment register, or GxAlert database at each study site.

- **Number completing testing**: Number of eligible patients with one valid Xpert result plus number of eligible patients with a valid smear result entered into the NTLP Presumptive TB register, NTLP Laboratory register, GeneXpert laboratory requisition forms, GxAlert database, or NTLP treatment register. Definitions of valid results are as follows:

  - A positive Xpert result with a semi-quantitative result of high, medium, low, or very low for all patients; a positive Xpert Ultra result with a semi-quantitative result of trace for HIV-positive patients; or second positive Xpert Ultra result if the initial result is trace-positive for HIV-negative patients;
  - A negative Xpert or Xpert Ultra result for all patients;[31]
  - One positive or two negative smear results for HIV-negative/HIV status unknown patients.

- ***Number diagnosed***: Number of eligible patients with microbiologically-confirmed TB via a positive smear and/or Xpert test result entered into the NTLP Presumptive TB register, NTLP Laboratory register, GeneXpert laboratory requisition forms, NTLP treatment register, and/or GxAlert database within 6 months of *date enrolled*.

- ***Number with suspected RIF resistant TB***: Number of eligible patients with RIF resistance identified by Xpert testing entered into the GxAlert database, GeneXpert laboratory requisition forms, and/or NTLP Laboratory registers within 6 months of *date enrolled*.

- ***Number with confirmed RIF resistant TB***: Number of eligible patients with RIF resistance identified by Xpert testing entered into the GxAlert database, GeneXpert laboratory requisition forms, and/or NTLP Laboratory registers, and RIF resistance confirmed by culture-based DST or a second molecular assay as determined by review of Lab Register at MDR treatment center within 6 months of *date enrolled*.

- ***Number treated***: Number of eligible patients entered into the NTLP Treatment register as having started Category I or II regimen, or started on MDR treatment at the MDR treatment center as as determined by review of Treatment Register at MDR treatment center within 6 months of *date enrolled.*

- ***Number completing treatment***: *Number treated* and with a treatment outcome of cured or completed entered into the NTLP Treatment register or indicated by MDR treatment center staff through direct follow up.

- ***Number died***: *Number treated* and with a treatment outcome of died entered into the NTLP Treatment register within 6 months of *date enrolled*, or number eligible with treatment outcome entered as died on direct follow-up form.

- ***Time-to-diagnosis***: Number of days from *date enrolled* to earliest date of positive smear or Xpert result recorded in NTLP Laboratory register, GeneXpert laboratory requisition form, NTLP treatment register, or GxAlert database within 6 months of *date enrolled*.

- ***Time-to-treatment***: Number of days from *date enrolled* to treatment start date entered into NTLP Treatment register if treatment start date is within 6 months of *date enrolled.*

I. Primary Outcome (Effectiveness)

Outcome	Numerator	Denominator
Proportion treated for microbiologically-confirmed TB within two weeks of referral for sputum-based testing	Number diagnosed and time-to-treatment within 14 days	Number referred for TB testing

II. Secondary Outcomes (Effectiveness)

**A. Testing**		
**Outcome**	**Numerator**	**Denominator**
Number referred for TB testing	Number referred for testing	None
Proportion completing testing	Number completing testing	Number referred for testing
**B. Diagnosis**		
**Outcome**	**Numerator**	**Denominator**
Number diagnosed with microbiologically-confirmed TB*	Number diagnosed	None
Proportion diagnosed with microbiologically-confirmed TB*	Number diagnosed	Number referred for testing
Number suspected/diagnosed with RIF-resistant TB*	Number with suspected/confirmed RIF resistant TB	None
Proportion suspected/diagnosed with RIF-resistant TB*	Number with suspected/confirmed RIF resistant TB	Number referred for testing
Time to microbiologically-confirmed TB	Time-to-diagnosis if microbiologically-confirmed TB	None
**C. Treatment**		
**Outcome**	**Numerator**	**Denominator**
Number treated for TB*	Number treated	None

Proportion treated for TB*	Number treated	Number enrolled
Number treated for microbiologically-confirmed TB*	Number diagnosed AND treated	None
Proportion treated for microbiologically-confirmed TB*	Number diagnosed AND treated	Number referred for testing
Proportion with microbiologically-confirmed TB treated*	Number diagnosed AND treated	Number diagnosed
Time-to-treatment of microbiologically-confirmed TB	Time-to-treatment if microbiologically-confirmed TB and treated	None
**D. Follow-up**		
**Outcome**	**Numerator**	**Denominator**
Number with microbiologically-confirmed TB completing treatment	Number diagnosed AND completing treatment	None
Proportion with microbiologically-confirmed TB completing treatment	Number diagnosed AND completing treatment	Number diagnosed AND treated
Number who died within 6 months	Number died	None
Proportion who died within 6 months**	Number died	Number enrolled

** Outcome will be assessed within 1 day and within 14 days of initial sputum submission. One day was chosen because the intervention focuses on same-day diagnosis and treatment. 14 days was chosen because the diagnostic process could take 7-10 days in the control arm depending on the frequency of sample transport to Xpert testing sites.*
***Treatment outcomes will be assessed at 7, 9, 12, and 24-month post-treatment initiation intervals determined by drug regimen.*

III. Implementation Outcomes

**Comparison across arms**	
**Outcome**	**Definition**
Patient costs<br>a) Total costs<br>b) Total direct costs<br>c) Total indirect costs	 a) Sum of all patient-reported costs on cost questionnaire<br>b) Total costs paid by the patient (i.e., excluding lost wages or other lost opportunities to earn income)<br>c) Patient-reported lost wages or other lost opportunities to earn income
Patient satisfaction with care survey:<br>a) General satisfaction with care<br>b) Convenience of services<br>c) Health facility environment<br>d) Provider interpersonal skills<br>e) Provider technical competence	 a) Sum of score on 3 question items (Q16, 17 and 18)<br>b) Sum of score on 3 question items (Q1, 2 and 3)<br>c) Sum of score on 2 question items (Q4 and 5)<br>d) Sum of score on 5 question items (Q7-11)<br>e) Sum of score on 4 question items (Q12-15)
Provider survey:<br>a) Intention	 a) Score on 1 question item (Q4)

b) Attitudes/beliefs	b) Sum of score on 3 question items (Q5a, 6a, and 6b)
c) Social norms/expectations	c) Sum of score on 2 question items (Q5b, 6c and 6d)
d) Self-efficacy/behavioral control	d) Sum of score on 5 question items (Q5c, 6e and 6f)

Intervention arm only		
**Outcome**	**Numerator**	**Denominator**
Process metrics:		
a) GeneXpert device non-operation days	a) GeneXpert device non-operation days[1]	a) Total number of lab operation days[1]
b) Proportion tested by GeneXpert:<br>1. On same day as initial sputum submission<br>2. By next day after initial sputum submission	b) Number with GeneXpert result date<br>1. Same as initial sputum submission date[2]<br>2. By next day after initial sputum submission date[2]	b) Number tested by GeneXpert [2]
c) Proportion with indeterminate results	c) Number with coded error, invalid or no test result[2]	c) Number of initial GeneXpert tests done[2]
d) Proportion with coded error result	d) Number with coded error result[2]	d) Number of initial GeneXpert tests done[2]
e) Proportion with invalid result	e) Number with invalid result[2]	e) Number of initial GeneXpert tests done[2]
f) Proportion with “no test” result	f) Number with “no test” result[2]	f) Number of initial GeneXpert tests done[2]
g) Proportion with GeneXpert test repeated if initial result indeterminate	g) Number with second GeneXpert result[2]	g) Number with initial indeterminate GeneXpert result[2]
h) Proportion treated on same-day if Xpert-positive	h) # treated on same day as initial health center visit[3]	h) # Xpert-positive[2]
i) Proportion of report cards reviewed at staff meetings	i) Number of Report Cards with Comments section filled[4]	i) Total number of report cards issues to health centers[3]
Variation in and barriers to intervention strategy uptake	Themes emerging from focus group discussions, semi-structured interviews with health center staff	

Source of data: [1] Health center laboratory log; [2] GeneXpert Software; [3] Treatment register; [4] Performance feedback report card

IV. Cost-effectiveness/Modeling Outcomes

Outcome	Definition	Source
Incremental cost per DALY averted	(Cost in intervention – cost in control)/(DALYs averted by intervention – DALYs averted in control) from societal perspective	Markov-based decision models
Incremental health system cost per DALY averted	As above, but from health system/provider perspective	
Incremental patient cost per	As above, but from patient	

DALY averted	perspective	
Projected reduction in TB incidence over 10 years	(Projected incidence in control model – projected incidence in intervention model)/(Projected incidence in control model), cumulative over 10 years	Transmission models
Projected reduction in TB mortality over 10 years	(Projected mortality in control model – projected mortality in intervention model)/(Projected mortality in control model), cumulative over 10 years	

Data Management

Dr. Fielding (statistician) will oversee data management in conjunction with UCSF- and Uganda-based study coordinators using the NIH-recommended Research Electronic Data Capture (**REDCap**) software, password-protected and accessible only to research staff. All data collection forms will be entered into standardized REDCap forms, with validation of data using range and consistency checks. Quality control procedures will include review of all study data collection forms for completeness and accuracy prior to data capture. Study staff will begin data capture by compiling photos from each individual health center for each data collection period. Using patient name, age, and sex, the staff will create one unique REDCap database entry (*i.e.* record) per patient by tracking patients across data sources in the following order: 1) Presumptive TB Register; 2) Laboratory Register; 3) Xpert Referral form; and 4) Treatment Register. If the patient cannot be located in a subsequent data source, the staff will check the next, until all data sources are consulted. If a patient does not meet inclusion criteria for analysis due to examination type (outreach, transferred in, follow up, or other), the staff will not collect data beyond the patient's name, sex, age, and exam type from the Laboratory Register. Once patients have been matched across all data sources, the staff will run a series of reports in REDCap to verify the completeness and accuracy of key variables impacting patient eligibility and/or study outcomes: age, sex, HIV status, examination type, smear and Xpert test results, outcomes dates (sputum collection, test results, start and end of treatment, treatment outcome), and treatment status. Study staff will review the original data photographs to verify the information is missing, and they will compile a list of follow up items for each health center. Research staff will phone health center staff after each 2-week period of data is extracted to resolve missing information and clarify any discrepancies or uncertainties in matching. Once the health center reviews the missing and/or inaccurate data, the study staff will update REDCap. The UCSF study coordinator will visit Uganda 2-3 times a year and review a random sample of forms and primary data sources for quality assurance.

Statistical Analysis

Aim 1

A detailed Statistical Analysis Plan will be developed. Briefly, we will calculate and compare all effectiveness outcomes for the two trial arms using an intention-to-treat analysis. We will use

descriptive statistics and 95% confidence intervals to summarize the yield, efficiency, and speed outcomes for the two intervention arms by site. To assess the intervention effect on outcomes, we will use methods appropriate for randomization of a small number of clusters and accounting for the stratified design.[26] A cluster-level analysis, giving each cluster equal weight, will be conducted to calculate unadjusted ratio and difference effect measures, and their associated 95% confidence intervals. Adjusted effect estimates will also be calculated taking into account any imbalance of important factors at baseline by study arm. Pre-specified subgroup analyses (*e.g.,* gender and HIV status) will be conducted for the primary outcome. A detailed statistical analysis plan will document methods used for the analysis of primary and secondary outcomes, and document pre-specified sub-group analyses.

Aim 2

1) Descriptive analyses – We will report process metrics on a monthly basis to assess adoption and maintenance of each intervention component overall, within key patient sub-groups, and at individual sites. We will report median and change between pre- and post-randomization assessments in **A)** patient costs; **B)** patient satisfaction with care; and **C)** provider TPB construct scores at control and intervention sites.

2) Comparative analyses – To identify patient-, provider-, and/or clinic-level factors independently associated with adoption and maintenance of intervention components, we will develop linear or logistic regression models, taking into account the clustered design (for example, robust standard errors). To compare by study arm the change from baseline (pre-randomization) to post-intervention in **A)** patient cost; **B)** patient satisfaction with care; and **C)** TPB construct scores, we will use a cluster-level analysis, similar to methods described for the primary outcome under Aim 1. The analysis will take into account the stratified design and an adjusted analysis will also be conducted taking into account other baseline imbalances by study arm.

3) Qualitative analyses – De-identified focus group or interview transcripts will be uploaded to the qualitative data analysis software Dedoose (SocioCultural Research Consultants, USA). Thematic interpretation[32-34] will include collaborative development of a coding framework and detailed coding of transcripts using Dedoose. Coded transcripts will be sorted to identify thematic groupings. The thematic groupings will be reviewed to identify emergent themes within each domain of the coding framework and quotes that best represent each domain. Thematic interpretation will focus on individual, social and structural factors associated with successful or unsuccessful adoption and/or maintenance of the intervention components at different sites.

Aim 3

Cost-effectiveness -- Our primary outcome will be the incremental cost-effectiveness of the intervention strategy from a societal perspective, measured as the cost per disability-adjusted life year (DALY) averted relative to standard TB evaluation. Secondary outcomes will include 1) the incremental cost of introducing and maintaining the intervention strategy (a measure of affordability for health systems) and 2) the incremental patient cost per diagnostic evaluation and per treatment initiated (a measure of affordability and access for patients). For all outcomes, we will report stratified results for key populations including women and people living with HIV. To assess cost-effectiveness, we will use the primary effectiveness data from Aim 1

and relevant literature estimates (e.g., clinical outcomes among those initiating treatment) to construct a Markov model including states for undiagnosed TB but seeking care, undiagnosed TB not seeking care (e.g., because initial diagnostic evaluation was too expensive), treated TB, self-resolved TB, and death. States will be subdivided according to smear and Xpert status (smear-positive, smear-negative/Xpert-positive, and smear/Xpert-negative) and HIV status (positive and negative, on/off ART, with CD4 strata). Data to inform transition probabilities and health utilities will come from study data where feasible (including specific questions of patients to ascertain probabilities such as future care-seeking if diagnoses are missed), and the literature where unavailable. We will follow international conventions for all procedures including economic costing, discounting, and reporting. We will conduct one-way sensitivity analyses across all model parameters, multi-way sensitivity analyses for those parameters found to be most influential, and a probabilistic uncertainty analysis in which all parameters are varied simultaneously using Latin Hypercube Sampling.

Population-Level Epidemiological Impact – We will construct a compartmental epidemic model of TB in Uganda to evaluate the potential impact of scaling up the intervention strategy across a representative district in Uganda. Using our team's prior models of TB diagnostics in India[35] and sub-Saharan Africa as a starting point, we will construct a population model that includes structure both for TB natural history (e.g., latent, subclinical, pre-diagnostic, diagnosis-seeking, treated)[36] and steps in the diagnostic cascade (e.g., pursuing diagnosis, diagnosed but not treated, treated).[35, 37] We will include structure for both HIV and MDR TB, and will link this model's structure to that of the Markov model constructed for cost-effectiveness analysis above (for purposes of explicitly estimating the importance of transmission to considerations of cost-effectiveness). We will fit the model to epidemiological data from a selected representative district in Uganda according to its TB incidence, prevalence, and mortality, as well as additional factors including prevalence of HIV and of MDR-TB. This model will project TB incidence and mortality over a primary time-frame of 10 years under two alternative scenarios: 1) standard TB diagnostic evaluation and 2) streamlined TB diagnostic evaluation using the intervention strategy. In a secondary analysis, we will incorporate economic data as described above, comparing cost-effectiveness measured under a dynamic (epidemic-economic) framework to that of the Markov model. The dynamic transmission model has the advantage of incorporating transmission dynamics at the population level, but requires more assumptions (*e.g.*, homogeneous mixing in the source population). By comparing results from Markov and transmission modeling, we will be able to assess: 1) the relative contribution of population-level transmission to the overall effectiveness and cost-effectiveness of different TB diagnostic evaluation strategies over time and 2) the relative influence of given model parameters on cost-effectiveness under a cohort-based versus transmission-based evaluation model.

Sample Size Considerations

Aim 1

The study is based on the health clinic being the unit of randomization and aims to demonstrate the superiority of the intervention arm. The sample size calculation uses formulae appropriate

for cluster-randomized trials with a parallel design and stratified and/or restricted randomization, including the addition of one extra cluster per arm to allow for the loss of degrees of freedom due to stratification to obtain conservative estimates.[26] The outcome is the proportion of patients treated for microbiologically-confirmed TB within two weeks of referral for sputum-based testing. A type I error of 5% and power of 90% is assumed. Pre-randomization data collected from February to July 2017 from the 20 selected trial sites in Uganda suggests the average proportion of patients referred for TB evaluation who initiate treatment for active TB within two weeks is 8.6%, and the coefficient of variation (k) between clusters is 0.27, with a harmonic mean of 325 patients enrolled at each health center cluster during the 18-month enrolment period. Based on these assumptions and assuming a type I error of 5%, we will have 90% power to detect a 6% or greater absolute increase in the outcome proportion in the intervention arm with a trial duration of 18 months (see **Table**). Table 1 also shows detectable absolute effect sizes with varying power (80%, 85%, 90%) during various trial time intervals and respective average cluster size estimates.

Average cluster size	Effect size (10 clusters per arm, *k*=0.27)		
	80% Power	85% Power	90% Power
215 (*12 months*)	5.3%	5.8%	6.4%
270 (*15 months)*	5.1%	5.5%	6.2%
325 (*18 months*)	4.9%	5.4%	6.0%
375 (*21 months*)	4.8%	5.3%	5.8%
430 (*24 months*)	4.7%	5.2%	5.7%

Aim 2

The sample size for Aim 2 analyses is either fixed (process metrics; provider surveys and focus groups) by parameters of the clinical trial or based on feasibility considerations (patient surveys and provider in depth interviews). For quantitative analyses, the sample size is sufficiently large (data on 3400 patients for process metrics analyses, 800 patient surveys, and 100-200 provider surveys) to enable multivariable analysis to identify factors associated with intervention adoption and maintenance.

Aim 3

As Aim 3 is primarily a modeling aim, sample size calculations are not applicable. Sample size considerations for the cost data (i.e., number of direct observations through time and motion studies, number of patients completing the cost questionnaires) are based on feasibility considerations and desire to estimate model parameters to sufficient levels of precision, as described above for Aim 2.

Ethical Considerations

Potential risks to participants

There are minimal risks to participants in this study. The primary risk to patients undergoing TB diagnostic evaluation during the study period is the potential for loss of confidentiality and stigma should their personal health information, including HIV or TB status, be disclosed. Patients participating in surveys also have the potential of sensitive information regarding their income being disclosed. Finally, the primary research risks for health workers related to the surveys and qualitative studies are punitive actions by the employer in response to the information they provide for research.

Protection against risk

The trial will be submitted for approval to the Research Ethics Committees of the University of California San Francisco and Makerere University College of Health Sciences, and to the Uganda National Council for Science and Technology. It is registered with the U.S. National Institutes of Health's ClinicalTrials.gov and the Pan African Clinical Trials Registry (PACTR) as a Phase 4 clinical trial. All study staff will be required to have completed Good Clinical Practice (GCP) training.

To minimize the potential for loss of confidentiality, all patient-identifiable data will be stored in locked or password protected areas accessible only to study personnel. Patient names will be used to match patient records across NTLP Laboratory and Treatment registers, but will not be included in the password-protected, electronic study database. Primary data collection forms used when matching records across data sources will be destroyed once entry of data into the electronic study database is completed.

To minimize risks to autonomy for patients and providers who participate in surveys, research staff will be carefully trained in how to administer the consent form to the individuals in the different target populations, with attention given to the background and principles of research ethics.

The Directors of participating health centers will be asked to sign a Memorandum of Understanding agreeing that their health center participate in the trial and agreeing not to introduce any new TB evaluation interventions during the time period of the trial without informing trial staff. Individual patients evaluated for TB at participating health centers will not be consented because the trial meets the requirements to qualify for waiver of informed consent under U.S. Department of Health and Human Services (DHHS) Regulation 46.116 (d): 1) No data or samples will be collected specifically for research purposes; 2) Patients will receive the same or higher quality of care; 3) It is not practical for health workers to obtain informed consent during the process of delivering routine clinical care; and 4) All pertinent TB testing results will be communicated to patients via routine or enhanced processes of care. Verbal informed consent using a script will be obtained from patients who participate in surveys. Written informed consent will be obtained from providers who participate in surveys, direct observation (*i.e.,* time-motion) studies and/or focus group discussions/interviews. The consent forms, which

will be approved by institutional review boards (IRB) in the U.S. and Uganda, will be translated from English into the local languages, and back-translated into English as required by IRBs to be sure that no significant language or concepts are lost in translation.

Data protection

All patient-identifiable data will be stored in locked or password-protected cabinets or databases accessible only to study personnel. Patient names will be used to match patient records across NTLP Laboratory and Treatment registers, but will not be included in the password-protected, electronic study database. Primary data collection forms and images used when matching records across data sources will be destroyed once entry of data into the electronic study database is completed.

Potential benefits of the study to participants and society

Patients undergoing TB diagnostic evaluation at participating health centers may benefit from the study through potential enhanced diagnosis and treatment of TB, either because of training (control health centers) or training plus other interventions (intervention health centers). Earlier diagnosis and treatment of TB may lead to improved patient outcomes and reduced disease transmission in patients' communities.

Patients and providers who participate in surveys, in depth interviews or focus group discussions will receive sodas and/or lunch to compensate for their time. Otherwise, they will not directly benefit from participating in these research activities.

Potential benefits to society include identification of strategies to improve TB case finding and to decrease TB incidence and prevalence in low-income, high-burden countries. If successful, the proposed intervention could potentially be scaled up to improve TB care in similar settings.

Dissemination

The trial results will be communicated to stakeholders through dissemination meetings and to participating health centers using language-appropriate information sheets. Investigators will present results at relevant conferences, and submit manuscript(s) to peer-reviewed journals. Public access to the participant-level dataset of main trial results and statistical code will be made available.

Trial governance

Because of the low-risk nature of the research, the Principal Investigator will be responsible for monitoring the data, assuring protocol compliance, and conducting safety reviews on a quarterly basis. An independent Trial Steering Committee (TSC) will meet approximately every 6 months and as needed. Prior to each meeting, the Principal Investigator will submit a progress report, including recommendations on whether the project should continue unchanged, require modification/amendment, or close to enrollment to an independent Trial Steering Committee (TSC). All major modifications (e.g., study design, sample size, study termination or suspension), will be approved by the TSC and ethics committees.

Abbreviations

AFB	Acid-Fast Bacilli
ART	Anti-retroviral therapy
CFR	Code of Federal Regulations
CRT	Cluster-randomized trial
DALY	Disability-adjusted life year
DHHS	Department of Health & Human Services
DMC	Data Monitoring Committee
EQA	External quality assurance
FDA	U.S. Food and Drug Administration
GCP	Good Clinical Practice
ICH	International Council for Harmonisation of Technical Requirements for Pharmaceuticals for Human Use
IRB	Institutional review board
ISTC	International Standards for TB Care
KAP	Knowledge, attitudes, and practices
LED	Light-emitting diode
MDR-TB	Multi-drug-resistant tuberculosis
MOU	Memorandum of Understanding
MUREC	Makerere University Research Ethics Committee
NIH	National Institutes of Health
NHLBI	National Heart, Lung, and Blood Institute
NTLP	National Tuberculosis and Leprosy Program
NTP	National TB Program
NTRL	National TB Reference Laboratory
PRECEDE	Predisposing, Reinforcing, and Enabling Constructs in Educational/Environmental Diagnosis and Evaluation
PSQ	Patient Satisfaction Questionnaire
RE-AIM	Reach Effectiveness Adoption Implementation Maintenance
REDCap	Research Electronic Data Capture
TB	Tuberculosis
TPB	Theory of Planned Behavior
TSC	Trial Steering Committee
UCSF	University of California San Francisco
UCSF CHR	UCSF Committee on Human Research
UNCST	Uganda National Council for Science and Technology
WHO	World Health Organization
XPEL TB	GeneXpert Performance Evaluation for Linkage to Tuberculosis Care
ZN	Ziehl–Neelsen

Competing interests

The investigators declare that they have no competing interests.

Trial Steering Committee

Jerry Friedland (Yale University)
Noah Kiwanuka (Makerere University)
Madhukar Pai (McGill University)
Andrew Ramsay (WHO/TDR)
Grant Theron (Stellenbosch University)

References

1. WHO. Global tuberculosis report 2016. Geneva, Switzerland: World Health Organization; 2016.
2. MacPherson P, Houben RM, Glynn JR, Corbett EL, Kranzer K. Pre-treatment loss to follow-up in tuberculosis patients in low- and lower-middle-income countries and high-burden countries: a systematic review and meta-analysis. Bull World Health Organ. 2014;92(2):126-38. doi: 10.2471/BLT.13.124800. PubMed PMID: 24623906; PMCID: PMC3949536.
3. Cattamanchi A, Dowdy DW, Davis JL, Worodria W, Yoo S, Joloba M, Matovu J, Hopewell PC, Huang L. Sensitivity of direct versus concentrated sputum smear microscopy in HIV-infected patients suspected of having pulmonary tuberculosis. BMC Infect Dis. 2009;9:53. Epub 2009/05/08. doi: 1471-2334-9-53 [pii]10.1186/1471-2334-9-53. PubMed PMID: 19419537; PMCID: 2690598.
4. Chihota VN, Ginindza S, McCarthy K, Grant AD, Churchyard G, Fielding K. Missed Opportunities for TB Investigation in Primary Care Clinics in South Africa: Experience from the XTEND Trial. PloS one. 2015;10(9):e0138149. doi: 10.1371/journal.pone.0138149. PubMed PMID: 26383102; PMCID: PMC4575203.
5. Davis J, Katamba A, Vasquez J, Crawford E, Sserwanga A, Kakeeto S, Kizito F, Dorsey G, den Boon S, Vittinghoff E, Huang L, Adatu F, Kamya MR, Hopewell PC, Cattamanchi A. Evaluating tuberculosis case detection via real-time monitoring of tuberculosis diagnostic services. American journal of respiratory and critical care medicine. 2011;184(3):362-7. doi: 10.1164/rccm.201012-1984OC. PubMed PMID: 21471088; PMCID: 3175538.
6. Aspler A, Menzies D, Oxlade O, Banda J, Mwenge L, Godfrey-Faussett P, Ayles H. Cost of tuberculosis diagnosis and treatment from the patient perspective in Lusaka, Zambia. The international journal of tuberculosis and lung disease : the official journal of the International Union against Tuberculosis and Lung Disease. 2008;12(8):928-35. PubMed PMID: 18647453.
7. Chandrasekaran V, Ramachandran R, Cunningham J, Balasubramaniun R, Thomas A, Sudha G, Jegannatha Rao K, Perkins M, PR N. Factors leading to tuberculosis diagnostic drop-out and delayed treatment initiation in Chennai, India. The international journal of tuberculosis and lung disease : the official journal of the International Union against Tuberculosis and Lung Disease. 2005;9(S1):172.
8. Kemp JR, Mann G, Simwaka BN, Salaniponi FM, Squire SB. Can Malawi's poor afford free tuberculosis services? Patient and household costs associated with a tuberculosis diagnosis in Lilongwe. Bull World Health Organ. 2007;85(8):580-5. PubMed PMID: 17768515; PMCID: 2636388.
9. Shete PB, Haguma P, Miller CR, Ochom E, Ayakaka I, Davis JL, Dowdy DW, Hopewell P, Katamba A, Cattamanchi A. Pathways and costs of care for patients with tuberculosis symptoms in rural Uganda. The international journal of tuberculosis and lung disease : the official journal of the International Union against Tuberculosis and Lung Disease. 2015;19(8):912-7. doi: 10.5588/ijtld.14.0166. PubMed PMID: 26162356.
10. Simwaka BN, Bello G, Banda H, Chimzizi R, Squire BS, Theobald SJ. The Malawi National Tuberculosis Programme: an equity analysis. Int J Equity Health. 2007;6:24. Epub 2008/01/01. doi: 1475-9276-6-24 [pii]10.1186/1475-9276-6-24. PubMed PMID: 18163918; PMCID: 2253525.
11. Botha E, den Boon S, Lawrence KA, Reuter H, Verver S, Lombard CJ, Dye C, Enarson DA, Beyers N. From suspect to patient: tuberculosis diagnosis and treatment initiation in health facilities in South Africa. The international journal of tuberculosis and lung disease : the official journal of the International Union against Tuberculosis and Lung Disease. 2008;12(8):936-41. Epub 2008/07/24. PubMed PMID: 18647454.

12. Den Boon S, Semitala F, Cattamanchi A, Walter N, Worodria W, Joloba M, Huang L, Davis JL. Impact Of Patient Drop-out On The Effective Sensitivity Of Smear Microscopy Strategies. American journal of respiratory and critical care medicine. 2010;181:A2258.
13. Ouyang H, Chepote F, Gilman RH, Moore DA. Failure to complete the TB diagnostic algorithm in urban Peru: a study of contributing factors. Trop Doct. 2005;35(2):120-1. Epub 2005/06/23. doi: 10.1258/0049475054037002. PubMed PMID: 15970047.
14. WHO. WHO monitoring of Xpert MTB/RIF roll-out. Available at: http://www.who.int/tb/areas-of-work/laboratory/mtb-rif-rollout/en/ [cited 2015 January 15].
15. Steingart KR, Schiller I, Horne DJ, Pai M, Boehme CC, Dendukuri N. Xpert(R) MTB/RIF assay for pulmonary tuberculosis and rifampicin resistance in adults. The Cochrane database of systematic reviews. 2014;1:CD009593. doi: 10.1002/14651858.CD009593.pub3. PubMed PMID: 24448973.
16. Dowdy DW, Cattamanchi A, Steingart KR, Pai M. Is scale-up worth it? Challenges in economic analysis of diagnostic tests for tuberculosis. PLoS medicine. 2011;8(7):e1001063. doi: 10.1371/journal.pmed.1001063. PubMed PMID: 21814496; PMCID: 3144197.
17. Keeler E, Perkins MD, Small P, Hanson C, Reed S, Cunningham J, Aledort JE, Hillborne L, Rafael ME, Girosi F, Dye C. Reducing the global burden of tuberculosis: the contribution of improved diagnostics. Nature. 2006;444 Suppl 1:49-57. doi: 10.1038/nature05446. PubMed PMID: 17159894.
18. Churchyard GJ, on behalf of the Xtend study team. Xpert MTB/RIF vs microscopy as the first line TB test in South Africa: mortality, yield, initial loss to follow up and proportion treated. The Xtend Study. Conference on Retroviruses and Opportunistic Infections; Boston, USA: Available at: http://www.stoptb.org/wg/gli/assets/documents/M6/Churchyard%20-%20XTEND%20study.pdf; 2014.
19. Cepheid. GeneXpert Omni: The True Point of Care Molecular Diagnostic System: Cepheid Inc; 2015. Available from: http://www.cepheid.com/us/genexpert-omni.
20. Glasgow RE, Vogt TM, Boles SM. Evaluating the public health impact of health promotion interventions: the RE-AIM framework. American journal of public health. 1999;89(9):1322-7. PubMed PMID: 10474547; PMCID: 1508772.
21. Godin G, Belanger-Gravel A, Eccles M, Grimshaw J. Healthcare professionals' intentions and behaviours: a systematic review of studies based on social cognitive theories. Implementation science : IS. 2008;3:36. doi: 10.1186/1748-5908-3-36. PubMed PMID: 18631386; PMCID: 2507717.
22. Ajzen I. The theory of planned behavior. Organizational Behavior and Human Decision Processes. 1991;50:179-211.
23. Green LW, Krueter M. Health Program Planning - An Educational and Ecological Approach. 4th ed. Philadelphia, USA: McGraw-Hill; 2005.
24. Davis DA, Thomson MA, Oxman AD, Haynes RB. Evidence for the effectiveness of CME. A review of 50 randomized controlled trials. Jama. 1992;268(9):1111-7. PubMed PMID: 1501333.
25. Cattamanchi A, Miller C, Tapley A, Haguma P, Ochom E, Ackerman S, Davis JL, Katamba A, Handley MA. Health worker perspectives on barriers to delivery of routine tuberculosis diagnostic evaluation services in Uganda: A qualitative study to guide clinic-based interventions. . BMC Health Services Research. (in press).
26. Hayes RJ, Moulton LH. Cluster Randomized Trials. Boca Raton, Florida, USA: CRC Press; 2009.
27. Ivers NM, Halperin IJ, Barnsley J, Grimshaw JM, Shah BR, Tu K, Upshur R, Zwarenstein M. Allocation techniques for balance at baseline in cluster randomized trials: a methodological review. Trials. 2012;13:120. doi: 10.1186/1745-6215-13-120. PubMed PMID: 22853820; PMCID: 3503622.

28. Sismanidis C, Moulton LH, Ayles H, Fielding K, Schaap A, Beyers N, Bond G, Godfrey-Faussett P, Hayes R. Restricted randomization of ZAMSTAR: a 2 x 2 factorial cluster randomized trial. Clinical trials. 2008;5(4):316-27. doi: 10.1177/1740774508094747. PubMed PMID: 18697846.
29. Grogan S, Conner M, Norman P, Willits D, Porter I. Validation of a questionnaire measuring patient satisfaction with general practitioner services. Qual Health Care. 2000;9(4):210-5. PubMed PMID: 11101705; PMCID: PMC1743536.
30. Nabbuye-Sekandi J, Makumbi FE, Kasangaki A, Kizza IB, Tugumisirize J, Nshimye E, Mbabali S, Peters DH. Patient satisfaction with services in outpatient clinics at Mulago hospital, Uganda. Int J Qual Health C. 2011;23(5):516-23. doi: 10.1093/intqhc/mzr040. PubMed PMID: WOS:000294810300004.
31. World Health Organization. WHO Meeting Report of a Technical Expert Consultation: Non-inferiority analysis of Xpert MTB/RIF Ultra compared to Xpert MTB/RIF Geneva: World Health Organization; 2017. Available from: http://apps.who.int/iris/bitstream/10665/254792/1/WHO-HTM-TB-2017.04-eng.pdf.
32. Sandelowski M, Leeman J. Writing usable qualitative health research findings. Qualitative health research. 2012;22(10):1404-13. doi: 10.1177/1049732312450368. PubMed PMID: 22745362.
33. Sandelowski MJ. Justifying qualitative research. Research in nursing & health. 2008;31(3):193-5. doi: 10.1002/nur.20272. PubMed PMID: 18288640.
34. Voils CI, Sandelowski M, Barroso J, Hasselblad V. Making Sense of Qualitative and Quantitative Findings in Mixed Research Synthesis Studies. Field methods. 2008;20(1):3-25. doi: 10.1177/1525822X07307463. PubMed PMID: 18677415; PMCID: 2493048.
35. Salje H, Andrews JR, Deo S, Satyanarayana S, Sun AY, Pai M, Dowdy DW. The importance of implementation strategy in scaling up Xpert MTB/RIF for diagnosis of tuberculosis in the Indian health-care system: a transmission model. PLoS medicine. 2014;11(7):e1001674. doi: 10.1371/journal.pmed.1001674. PubMed PMID: 25025235; PMCID: 4098913.
36. Dowdy DW, Basu S, Andrews JR. Is passive diagnosis enough? The impact of subclinical disease on diagnostic strategies for tuberculosis. American journal of respiratory and critical care medicine. 2013;187(5):543-51. doi: 10.1164/rccm.201207-1217OC. PubMed PMID: 23262515; PMCID: 3733406.
37. Sun AY, Denkinger CM, Dowdy DW. The impact of novel tests for tuberculosis depends on the diagnostic cascade. The European respiratory journal. 2014;44(5):1366-9. doi: 10.1183/09031936.00111014. PubMed PMID: 25186263; PMCID: 4254765.

GeneXpert Performance Evaluation for Linkage to Tuberculosis Care:
The XPEL TB Trial

ClinicalTrials.gov Identifier:
NCT03044158

Pan African Clinical Trials Registry:
PACTR201610001763265

Sponsored by:
NIH/NHLBI

Unique Protocol ID:
R01HL130192

Principal Investigator:
Adithya Cattamanchi MD, MAS

Draft or Version Number:
Version 2.0

Day Month Year
September 11, 2019

Statement of Compliance

The study will be carried out in accordance with Good Clinical Practice (GCP) as required by the following:

- *U.S. Code of Federal Regulations applicable to clinical studies (45 CFR 46)*
- *ICH GCP E6*
- *Completion of Human Subjects Protection Training*
- *NIH Clinical Terms of Award*

Refer to: http://www.hhs.gov/ohrp/humansubjects/guidance/45cfr46.htm#46
http://www.fda.gov/cder/guidance/959fnl.pdf
http://grants.nih.gov/grants/guide/notice-files/NOT-OD-01-061.html
http://cme.cancer.gov/c01/

Signature Page

The signature below constitutes the approval of this protocol and the attachments, and provides the necessary assurances that this trial will be conducted according to all stipulations of the protocol, including all statements regarding confidentiality, and according to local legal and regulatory requirements and applicable US federal regulations and ICH guidelines.

Uganda Study Site Principal Investigator:*

Signed:___________________________________ Date:______________________

Achilles Katamba, MBChB, PhD

Overall Study Principal Investigator:*

Signed:___________________________________ Date:______________________

Adithya Cattamanchi, MD, MAS

Table of Contents

Overview....6
Key Roles....7
Table 1. Timeline of Activities....8
Background....9
Background information....9
Theoretical basis for intervention strategy....11
Table 2. Key clinic-level barriers targeted by intervention strategy.....12
Objectives....13
Methods/Design....14
Overview....14
Target Setting and Study Population....14
Eligibility criteria for the cluster-randomized trial....14
Eligibility criteria for mixed methods and health economic sub-studies....15
Recruitment....15
Health centers....15
Participants for cluster-randomized trial....16
Participants for mixed methods and health economic sub-studies....16
Intervention and Control (Wait-list) Arms....16
Figure 2. Comparison of TB diagnostic evaluation in the intervention and control arms....17
On-Site Molecular Testing....17
Process Redesign for Same-Day TB Diagnosis and Treatment....18
Performance Feedback....18
Randomization....19
Procedures....19
Health center training....19
Patient-level data collection....20
Symptom Screening....20
Patient surveys....21
Provider surveys....21
Health system costing....21
Focus group discussions....21

Assessment of patient's vital status, diagnostic and treatment status, HIV status, and ART status 22
Provider interviews 22
Figure 3. Site visit activities 22
Outcomes 23
Definitions 23
I. Primary Outcome (Effectiveness) 24
II. Secondary Outcomes (Effectiveness) 24
III. Implementation Outcomes 25
IV. Cost-effectiveness/Modeling Outcomes 27
Data Management 27
Statistical Analysis 28
Aim 1 28
Aim 2 28
Aim 3 29
Sample Size Considerations 30
Aim 1 30
Revised detectable effect size estimates: 30
Table 3: Detectable effect sizes 31
Aim 2 31
Aim 3 31
Ethical Considerations 32
Potential risks to participants 32
Protection against risk 32
Data protection 33
Potential benefits of the study to participants and society 33
Dissemination 33
Trial governance 33
Abbreviations 34
Competing interests 35
Trial Steering Committee 35
References 36

Overview

**Title**	GeneXpert Performance Evaluation for Linkage to Tuberculosis Care: The XPEL TB Trial
**Target Population**	Adults undergoing evaluation for pulmonary TB (N=11,283)
**Sites**	20 TB microscopy centers (10 intervention, 10 control) in Uganda
**Study Design**	Clustered-randomized trial with nested mixed methods and economic/transmission modeling studies
**Study Duration**	18 months
**Objectives**	**Aim 1: To compare patient outcomes at health centers randomized to intervention vs. standard-of-care TB diagnostic evaluation strategies.**<br>• *Intervention:* onsite molecular testing for TB + process redesign to facilitate same-day TB diagnosis and treatment + performance feedback<br>• *Standard-of-care*: onsite ZN or LED fluorescence microscopy + hub-based GeneXpert testing per existing protocols<br><br>**Aim 2: To identify processes and contextual factors that influence the effectiveness and fidelity of the intervention TB diagnostic evaluation strategy.**<br><br>**Aim 3: To compare the costs and epidemiological impact of intervention vs. standard-of-care TB diagnostic evaluation strategies.**

Key Roles

Principal Investigator

Adithya Cattamanchi, MD, MAS
Associate Professor of Medicine, University of California San Francisco
San Francisco General Hospital
1001 Potrero Ave, Room 5K1
San Francisco, CA 94110
Phone: +1-415-206-5489
Fax: +1-415-695-1551
adithya.cattamanchi@ucsf.edu

Uganda Principal Investigator

Achilles Katamba, MBChB, MSc, PhD
Senior Lecturer, Makerere University College of Health Sciences
Upper Mulago Hill Road
Kampala, Uganda
Phone: +256-(0)414-530-692
Fax: +256-(0)414-540-524
axk95@case.edu

NHLBI Program Officer

Elisabet Caler, PhD
AIDS Program Lung Team Leader
Lis.caler@nih.gov

Table 1. Timeline of Activities

	2018				2019				2020				2021			
	Q1	Q2	Q3	Q4	Q1	Q2	Q3	Q4	Q1	Q2	Q3	Q4	Q1	Q2	Q3	Q4
**Randomization and site training**																
Randomization unveiling ceremony			■													
Post randomization site visits (to introduce intervention or conduct guideline refresher training)			■													
**Assess patient-level outcomes**																
Collect TB diagnosis and treatment initiation data				■	■	■	■	■	■							
Collect 6-month vital status data						■	■	■	■	■	■					
**Assess intervention implementation (intervention sites)**																
Collect process metric data				■	■	■	■	■	■							
Conduct health worker interviews/focus groups									■							
**Assess whether targeted barriers are modified**																
Provider survey (all involved in TB evaluation)								■	■							
Patient cost & satisfaction survey						■	■									
**Assess cost-effectiveness and impact**																
Conduct health system costing studies								■	■							
**Data cleaning, analysis and manuscript preparation**										■	■	■	■	■	■	■

Background

Background information

Prompt diagnosis and treatment of TB patients is essential to making progress towards TB elimination. However, at least 4.3 million of the estimated 10.4 million new cases in 2015 were not detected and reported to the World Health Organization (**WHO**).[1] There are three overarching reasons for this large gap: TB patients are not being notified to public health authorities, not seeking care, or not being diagnosed and treated even after seeking care. The last reason represents a clear health system failure that is pervasive in high burden countries – a recent systematic review of published studies found that up to 38% of sputum smear-positive patients in Africa and 28% in Asia are lost to follow-up prior to treatment initiation.[2] Patients with smear-negative TB are even less likely to complete the diagnostic cascade of care and be linked to treatment.

A principal reason for these failures in linkage to care is the inadequacy of the current approach to TB diagnosis at community health centers. It is well known that sputum smear microscopy, the most common test for TB at community health centers worldwide, has important limitations that contribute to delays in TB diagnosis and treatment. First, smear microcopy has sub-optimal sensitivity, identifying only about 50% of patients who actually have TB.[3] Second, the typical process of sputum collection and smear examination is burdensome for patients and staff. Guidelines usually require staff to ask patients to submit a sputum specimen on the day of presentation, return the following morning to submit a second specimen, and return a third time for treatment initiation (if smear-positive) or to consider further workup (if smear-negative). In high burden countries, clinicians fail to refer up to half of patients reporting TB symptoms for sputum smear examination.[4, 5] In addition, the direct and indirect costs of this standard multi-day diagnostic evaluation process consume up to 3 months of household income for already poor patients.[6-10] It is therefore not surprising that a substantial proportion do not return after their initial health center visit to submit additional sputum specimens, collect results or initiate treatment if smear-positive.[5, 11-13]

To address these limitations, there has been substantial donor investment in scale-up of Xpert MTB/RIF (**Xpert**)[14], a semi-automated molecular assay endorsed by the WHO in 2010 and by the US FDA in 2013. Xpert identifies 90% of TB cases, has a 2-hour turn-around time, and can be performed with minimal training and human resource requirements.[15] Although this represents an important advance, modeling analyses have found that Xpert is unlikely to bend the TB incidence curve significantly, primarily because it cannot be deployed at community health centers in high burden countries.[16] Indeed, because of high device costs and infrastructure requirements, the vast majority of Xpert devices are being placed at district or higher-level facilities[14], which are accessed by <15% of the population.[17] Many countries have therefore adopted a hub-and-spoke model in which several community health centers (spokes) are linked to an Xpert testing site (hub). However, a cluster-randomized trial has found that Xpert implementation using a hub-and-spoke model did not impact mortality; the major reason was failure to link patients with confirmed TB to treatment.[18]

The next generation of molecular diagnostics has strong potential to close gaps in the TB diagnostic cascade of care. In particular, GeneXpert Omni is a portable, single-cartridge version of the GeneXpert platform that is already WHO- and FDA-approved, and widely used in Uganda and other high TB burden countries. Because it only has a single test module (instead of 4 or more), it is lower cost (subsidized price $5000 vs. $10000 or more for previous GeneXpert platforms) and has lower power requirements enabling it to be battery-operated.[19] These features increase the likelihood that onsite Xpert testing can eventually be scaled up as a replacement for sputum smear at community health centers.

Our *overall objective* is to assess the effectiveness, implementation and costs of a streamlined TB diagnostic evaluation strategy based around rapid, onsite molecular testing. The intervention strategy was developed based on theory-informed assessment of barriers to TB diagnostic evaluation at community health centers in Uganda and a process of engagement with local stakeholders. It includes: 1) Point-of-care molecular testing using GeneXpert as a replacement for sputum smear microscopy; 2) Re-structuring of clinic-level procedures to facilitate same-day TB diagnosis and treatment; and 3) Quarterly feedback of TB evaluation metrics to health center staff. Our central hypothesis is that the intervention strategy will have high uptake and increase the number of patients diagnosed with and treated for active pulmonary TB. To test this hypothesis, we will conduct a *pragmatic cluster-randomized trial* at community health centers that provide TB microscopy services in Uganda in partnership with the National TB Program (**NTP**). We utilize an *effectiveness-implementation hybrid design* in which, concurrent with the clinical trial, we will conduct nested mixed methods, health economic and modeling studies to assess 1) whether the intervention strategy modifies targeted barriers to TB diagnostic evaluation; 2) fidelity of implementation of the intervention components (*i.e,* the degree to which intervention components were implemented as intended vs. adapted across sites); and 3) cost-effectiveness and public health impact. We will use the RE-AIM framework to evaluate how the intervention strategy affects processes and outcomes important to patients and TB programs, and to determine its scale-up potential. RE-AIM encompasses 5 dimensions common to successful multi-level interventions: depth of reach into a target population; effectiveness; factors that promote adoption; resources needed for implementation; and factors that ensure maintenance over time.[20] Thus, our focus is on evaluating the impact of the proposed multi-level intervention, rather than evaluating the impact of its individual components. The process of guideline implementation and technology uptake is complex, and it is unlikely that desired outcomes can be achieved with a simple intervention. However, if the desired outcomes are achieved, the proposed mixed methods studies will identify which barriers were modified, providing some insight into which intervention components are critical. In addition, future studies can focus on testing simplified versions of the multi-level intervention that remove one or more components.

Theoretical basis for intervention strategy

The intervention strategy was designed based on a mixed methods study conducted at six health centers in Uganda to better understand reasons for gaps along the TB diagnosis cascade of care and to inform intervention design. The process was guided by the Theory of Planned Behavior as

Figure 1. Theory-informed barrier assessment and intervention design

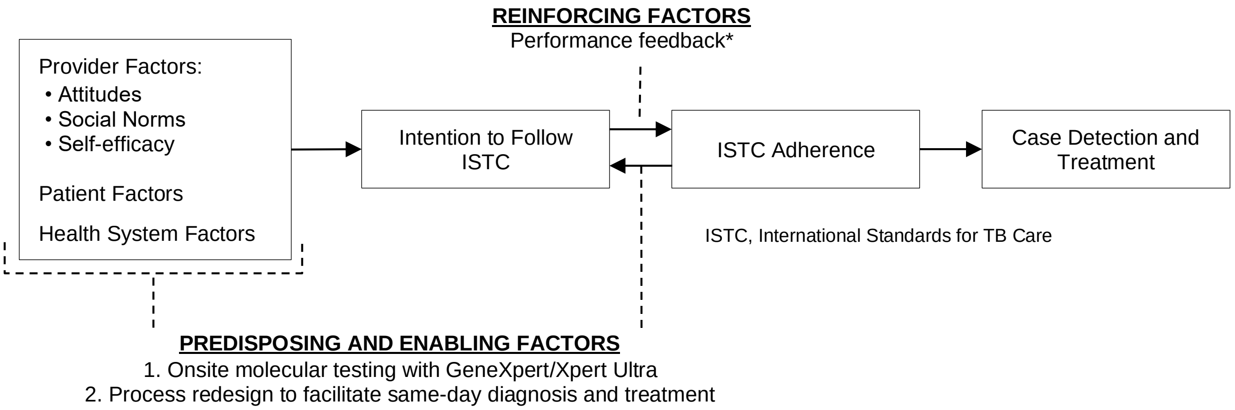

the conceptual framework and the PRECEDE model to select intervention components (**Figure 1**). In a systematic review of guideline implementation studies, the Theory of Planned Behavior was the most likely theory to predict guideline adherence.[21] This theory asserts that intention is the best predictor of behavior and that three factors mediate the strength of intention: 1) *attitudes* – expected value of behavioral performance; 2) *subjective norms* – what others think about the behavior; and 3) *self-efficacy* – perception of ability to overcome barriers to behavioral performance.[22] The PRECEDE model was chosen based on its strong empirical base and applicability to guideline adherence. The model is based on three factors relevant to health behavior change: 1) *predisposing* factors – prior motives that either support or inhibit behavior; 2) *reinforcing* factors – rewards or punishments following a behavior or anticipated as a consequence of it; and 3) *enabling* factors – objective characteristics of an individual or environment that facilitate behavior.[23] A meta-analysis of 50 randomized controlled trials of continuing medical education demonstrated that the studies employing a combination of interventions representing PRECEDE categories were the most likely to influence patient outcomes.[24]

The intervention strategy was designed in consultation with the Uganda NTP to target modifiable clinic-level barriers identified in the formative assessment as well as key theoretical constructs. Key barriers to TB evaluation identified through semi-structured interviews with 22 staff at the six health centers and organized within PRECEDE model categories are shown in **Table 2**.[25] The intervention strategy includes: 1) onsite molecular testing to reduce laboratory workload and increase the sensitivity of TB diagnostic testing; 2) re-structuring clinic-level procedures to facilitate same-day TB diagnosis and treatment, thereby increasing provider self-efficacy/motivation and enabling linkage of confirmed TB patients to treatment; and 3) performance feedback to increase communication between staff and with NTP supervisors, thereby reinforcing uptake of onsite molecular testing and adherence to TB evaluation guidelines. Re-assessing these barriers as part of Aim 2 will help understand whether the XPEL TB intervention worked as intended and help explain why it was or was not effective in improving TB diagnostic evaluation outcomes.

Table 2. Key clinic-level barriers targeted by intervention strategy.

**PRECEDE framework**	**Recurring themes highlighting barriers targeted by the intervention strategy**
**Predisposing factors**<br>(Knowledge, attitudes, beliefs, intention)	• Time and resource constraints → low self-efficacy<br>• Low motivation of staff<br>• Low sensitivity of sputum smear microscopy<br>• Poor patient perception of care at government health centers
**Enabling Factors**<br>(Factors that if addressed make it easier to initiate the desired behavior)	• Failure of patients to return after initial visit (due to time and costs)<br>• Inability to track and follow-up patients → low-self-efficacy<br>*"When they have a cough for more than 2 weeks they are sent to the lab. But the problem is they get the first sample and sometimes, actually most times they don't bring the second sample."*
**Reinforcing Factors**<br>(Factors that if addressed make it easier to continue the desired behavior)	• Lack of communication and coordination among staff<br>• Insufficient oversight from NTP<br>*"…Actually at times we have met but we don't meet [regularly], only when we realize there is a problem that's when we communicate and say why is this happening, then we try to rectify."*

Objectives

Aim 1: To compare patient outcomes at health centers randomized to intervention vs. standard-of-care TB diagnostic evaluation strategies. We will randomize 20 community health centers to continue standard TB evaluation (routine microscopy plus referral of patients for Xpert testing per existing processes of care) or to implement the intervention strategy (1. Onsite molecular testing; 2. Re-structuring clinic-level procedures to facilitate same-day TB diagnosis and treatment; and 3. Performance feedback). We will compare reach and effectiveness based on the numbers and proportions of patients (N=11,283) who complete TB testing, are found to have TB, and have treatment initiated within one week of specimen provision.

Aim 2: To identify processes and contextual factors that influence the effectiveness and fidelity of the intervention TB diagnostic evaluation strategy. We will use quantitative process metrics to assess the adoption and maintenance over time of the core components of the intervention strategy. We will also collect quantitative and qualitative data to describe the fidelity of implementation of each component and faithfulness to our conceptual model.

Aim 3: To compare the costs and epidemiological impact of intervention vs. standard-of-care TB diagnostic evaluation strategies We will model the incremental costs and cost-effectiveness of intervention relative to standard-of-care TB diagnostic evaluation from the health system and patient perspective. We will then construct an epidemic model of the population-level impact of the intervention strategy on TB incidence and mortality.

Methods/Design

Overview

The study proposes to conduct a pragmatic, parallel cluster-randomized trial with nested mixed methods, health economic and modeling studies to evaluate the effectiveness, implementation and costs of the intervention strategy relative to standard TB diagnostic evaluation at community health centers that are part of Xpert referral networks in Uganda. The effectiveness of the intervention strategy will be assessed using routine data collected as part of mandatory reporting to the Uganda NTLP on consecutive patients who present to participating health centers during the 18-month trial period and meet eligibility criteria. Selected patients and providers who provide informed consent will also be surveyed, interviewed, and/or participate in focus groups to identify reasons for its success or failure across study sites (Aim 2), and to collect relevant cost data for cost-effectiveness analyses (Aim 3).

Target Setting and Study Population

The target setting is community health centers with TB microscopy units (*i.e.,* the lowest level of the health system where TB diagnostic services are provided by the Uganda NTLP). The unit of randomization will be community health centers (N=20). The unit of analysis will be patients undergoing TB diagnostic evaluation during the 18-month trial period (estimated total 11,283 over 18-month trial period).

Eligibility criteria for the cluster-randomized trial

A. *Site-level Inclusion Criteria*
    1. Use standard (multi-day) sputum smear microscopy as the primary method of TB diagnosis
    2. Participate in NTP-sponsored external quality assurance (**EQA**) for sputum smear microscopy
    3. Send samples to a district or regional hospital/health center for Xpert testing
B. *Site-level Exclusion Criteria*
    1. Do not agree to be randomized to standard-of-care vs. intervention arms
    2. Perform sputum smear examination on <150 patients per year (based on 2015 data)
    3. Diagnose <15 smear-positive TB cases per year (based on 2015 data)
C. *Patient-level Inclusion Criteria*
    1. Initiate evaluation for active pulmonary TB at a study health center
D. *Patient-level Exclusion Criteria*
    1. Have sputum collected for monitoring of response to anti-TB therapy
    2. Have sputum collected as part of active, community-based case finding (e.g., contact tracing, community outreach campaign)
    3. Referred to a study health center for TB treatment after a diagnosis is established elsewhere
    4. Started on TB treatment for extra-pulmonary TB only

In addition, patients age <18 years and those with a documented prior history of TB treatment (e.g., reason for Xpert testing or TB treatment marked as treatment failure, relapse, treatment after loss to follow-up, etc.) will be excluded from the primary analysis of all outcomes. Patients identified as RIF resistant by Xpert testing will be excluded from the primary analysis of all outcomes except for the comparison of the number and proportion of patients with RIF resistance between arms.

Eligibility criteria for mixed methods and health economic sub-studies

Patient Surveys: Same eligibility criteria as for the CRT.

Provider Survey: Providers at each study site who are (a) aged ≥18 years; (b) employed by the peripheral health center; and (c) involved in the conduct or supervision of health center work related to diagnosis and management of TB will be included.

Focus Group Discussion and Interviews: Same inclusion criteria as for the provider survey.

Recruitment

Health centers

Potential trial sites were identified from a list of Uganda NTLP-affiliated TB microscopy centers that refer samples to Xpert testing hubs. Study staff reviewed 2014 TB testing and treatment data reported to the Uganda NTLP to identify health centers that meet eligibility criteria (based on numbers of patients tested for TB and found to be smear-positive), focusing on those within 150 km of Kampala for study feasibility purposes. Study staff then obtained permission from District Health Officials and Health Center Directors for visiting health centers to confirm eligibility and assess interest in study participation. At the site assessment visit, study staff counted from the 2015 TB laboratory register the number of patients tested by microscopy and the number of smear-positive patients (excluding patients tested for treatment monitoring or as part of community-based active case finding activities). Health centers confirmed to be eligible and that expressed interest in participating in the study were reviewed with Uganda NTLP and NTRL Directors. Following their approval, research staff met with the District Health Officer (**DHO**) to inform him or her about the project and requested the participation of potential trial sites in the District. The DHO was asked to sign a Memorandum of Understanding (**MoU**) that describes key study procedures and expectations of participating sites. If the DHO signed the MoU, study staff scheduled an enrollment visit with potential trial sites in the District by calling the facility 3-4 days prior to the anticipated visit date. Project staff then contacted the Program Manager of the Uganda NTLP to arrange for an NTLP representative to participate in the enrollment site visit. During the site visit, project staff followed a standard script to present the project to health center staff, outlined the project aims, and described project procedures. Project staff presented a copy of the MoU signed by the DHO and endorsed by the Chief Administrative Officer and NTLP Program Manager to the health center in-charge. Of note, the final list of 20 sites to be randomized was determined in consultation with the trial statistician (to minimize potential for site-level variation that could impact power).

Participants for cluster-randomized trial

This is a pragmatic trial that will study outcomes of routine care. Thus, rather than recruiting participants, the intervention and control arms will be compared using data available in routine TB laboratory and treatment registers for consecutive patients who present to participating health centers during the two-year trial period and meet eligibility criteria (estimated N=11,283 patients).

Participants for mixed methods and health economic sub-studies

Patient Cost and Satisfaction Surveys: The patient cost and satisfaction surveys will be administered during the baseline (pre-randomization) period (N=20/site; 400 total) and in the second year after randomization (N=40/site, 800 total). Health center staff will assist with identification and recruitment of patients who are interested in participating in the surveys (from among patients providing sputum samples or receiving results). Participation will be voluntary. Research staff will then contact interested patients by phone at the end of their health center visit to review the verbal consent script, answer questions the patient may have, and administer the survey to consenting patients.

Provider Survey: The provider survey will be administered during the baseline (pre-randomization) period and in the second year after randomization. All eligible providers at each study site (N=5-10/site; 100-200 total) will be invited to participate in the survey. Participation will be voluntary and written informed consent will be obtained.

Provider Focus Group Discussions and Interviews: Focus group discussions will occur during the second year after randomization and semi-structured interviews after the trial is completed. All eligible providers at intervention sites (N=5-10/site; 50-100 total) will be invited to participate in focus group discussions. Providers (N=20-30) at low- and high-performing sites will be invited to participate in semi-structured interviews. Participation in focus group discussions and interviews will be voluntary and written informed consent will be obtained.

Intervention and Control (Wait-list) Arms

With the current standard-of-care (*i.e.*, control arm), patients make up to 4 health center visits to complete TB diagnostic evaluation (**Figure 2**). At the first visit, sputum is collected for smear microscopy. All patients are required to return for a second visit; patients are initiated on TB treatment if the first sputum is smear-positive or are asked to provide a second sputum specimen and return for a third visit to collect results if the first sputum is smear-negative. Patients who return for a third visit are initiated on TB treatment if the second sputum is smear-positive or are asked to provide a third sputum specimen that is sent out for Xpert testing. Patients are asked to return for a fourth visit to collect Xpert results. Patients who return for a fourth visit are initiated on TB treatment if Xpert results are positive (or referred to an MDR TB treatment center if Xpert testing indicates rifampin resistance). Of note, HIV-infected patients have sputum collected for Xpert testing at their first visit and all patients could be started on TB treatment empirically at any visit.

The intervention strategy seeks to facilitate diagnosis and treatment of TB at the initial health center visit (or referral of patients to MDR TB treatment center if rifampin resistance is detected by Xpert). As further described below, it includes the following components: 1) onsite molecular

testing as a replacement for microscopy; 2) process re-design; and 3) feedback of TB diagnostic evaluation quality indicators to health center staff.

Figure 2. Comparison of TB diagnostic evaluation in the intervention and control arms

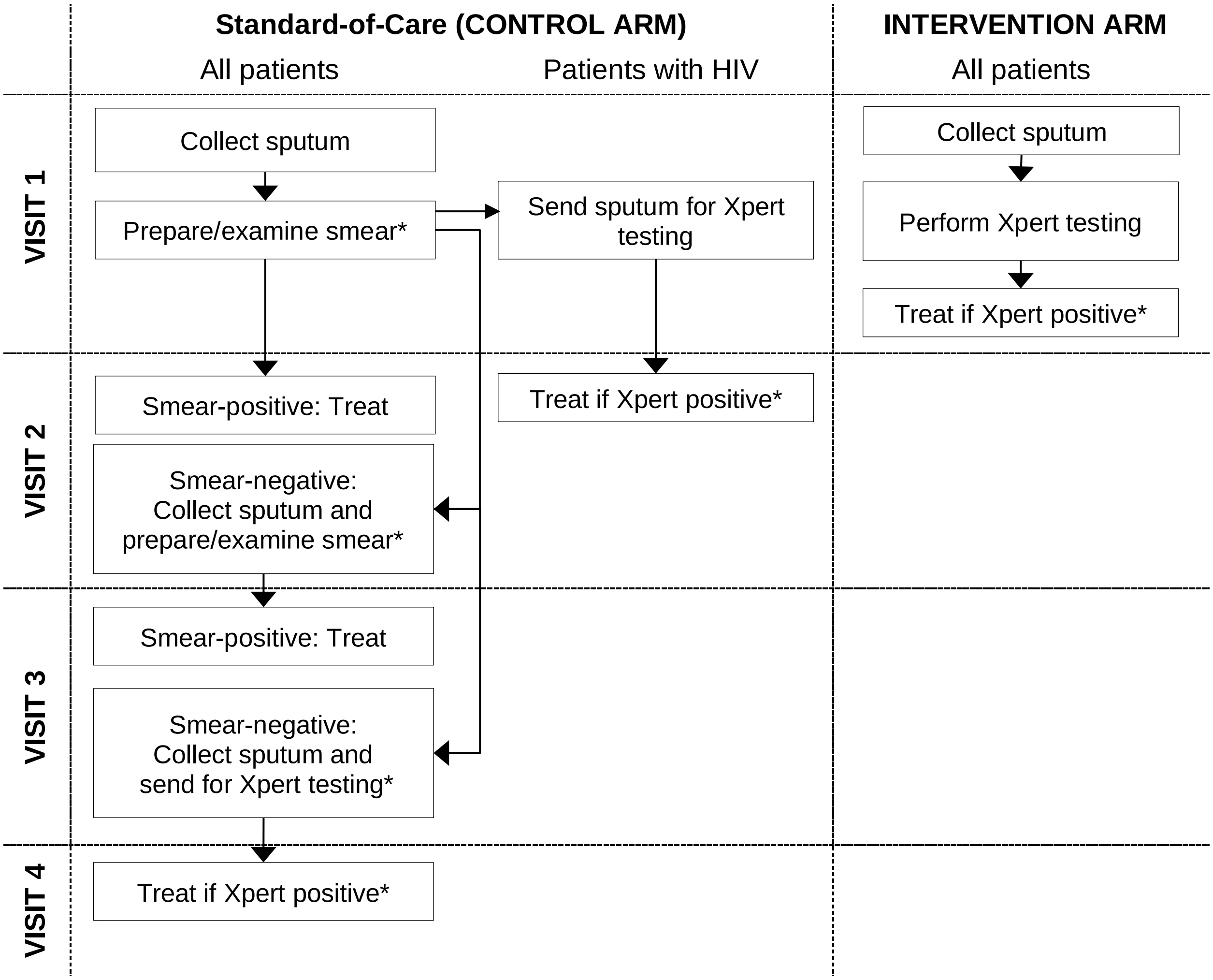

*Consider empiric treatment or additional testing if test results are negative or invalid

On-Site Molecular Testing

GeneXpert Omni (Cepheid, Sunnyvale, CA) is a point-of-care molecular diagnostic system designed for lower level health facilities. It is small (1.0 kg weight), portable, and consumes less power than previous GeneXpert systems. The standard configuration includes a single test cartridge module, a mobile device, a country-specific AC/DC power cord, a supplemental battery, and a user guide. The rechargeable battery allows for up to 4 hours of operation with voltage surge protection for unexpected power supply interruptions. Additionally, a supplemental battery can be charged for up to 12 hours of use in the event of sustained power loss. Data is stored in real-time via cloud-based connectivity to Cepheid Cloud Control using Wi-Fi or cellular networks. Training materials will be provided by Cepheid, with an anticipated training time of one half-day. Additional device-integrated videos and self-instructions are available for training reinforcement.[19]

Due to delays in release of GeneXpert Omni, the trial will begin with intervention sites using a modified conventional GeneXpert instrument (GeneXpert I). GeneXpert I is a one-module device with a specially-fitted dust cover that can be powered using a UPS/battery back and can be operated by a touchscreen tablet instead of a laptop computer. Sites randomized to the intervention arm will be given a solar panel charger to charge the UPS/battery pack. Once GeneXpert Omni is available, it will replace the GeneXpert I instrument. After the trial is completed, a GeneXpert Omni or GeneXpert I device will be provided to control health centers and health center staff will be trained on the use and maintenance of the device.

Of note, Xpert testing in both the control and intervention arms will occur using Xpert MTB/RIF Ultra, which is already being scaled-up in Uganda.

Process Redesign for Same-Day TB Diagnosis and Treatment

Research and Uganda NTLP staff will engage health center staff in a discussion of how to re-organize clinical, laboratory and pharmacy services to enable same-day TB diagnosis and treatment. Health center staff will be asked to identify site-specific solutions to:

1. Rapid screening for TB at registration desk and/or waiting area
2. Immediate referral of patients identified during screening as needing further testing to the lab for sputum collection
3. On-demand testing of sputum specimens and immediate reporting of results to clinicians and patients
4. Availability of TB pharmacy services throughout the clinic day

Agreed upon changes to existing procedures and staff responsibilities will be recorded and reviewed with the health center in-charge, lab director and TB pharmacist.

Performance Feedback

Performance feedback is a strategy employing regular monitoring and feedback to allow health care workers to critically analyze performance and identify areas for improvement. A systematic review of audit and feedback interventions identified 5 factors associated with greater impact: low baseline performance, feedback coming from a supervisor or colleague, feedback provided multiple times, feedback delivered in both verbal and written form, and feedback including explicit targets and an action plan.26 Thus, performance feedback will involve delivery of a monthly Report Card which displays 1) TB diagnostic evaluation quality indicators for the current month and for the previous 6 months; 2) performance data averaged across all intervention health centers; and 3) performance data for the top- performing intervention health center. The Report Card will include a section that asks staff to write down their interpretation of the performance data, identify barriers to performance improvement, and specify plans to improve performance. The Report Card will be introduced to health center staff at the post-randomization site visit and will then be sent electronically every month to the health center in- charge or TB focal person. Health workers will be asked to review the Report Card at monthly staff meetings to devise a performance improvement plan. This process will continue monthly, with each new Report Card being used to evaluate the success of plans developed in the previous month and determine the

need for new actions. Report Cards will be collected by study staff at quarterly site visits to assess use and completion.

Randomization

Health centers will be grouped into two strata using baseline data for the primary outcome (see Outcomes below), and randomized within strata to help reduce between-cluster variation and improve balance between arms at baseline.[26] Restriction, a common approach in cluster-randomized trials with a small number of clusters, may be used to help achieve baseline balance of important site- and patient-level covariates.[27] We will consider restricting on factors likely to be associated with the primary outcome including health center region (four quadrants of Uganda), health center size (based on volume of patients tested for TB), HIV prevalence among TB patients, and patient cost and satisfaction with care. The restriction factor will be calculated and the validity of the restriction assessed using the validity matrix.[28] The Trial Statistician (KF) will be responsible for the randomization. Randomization will be unveiled to health center directors (or their designated representative) at a meeting chaired by the Uganda NTLP Director.

Procedures

The following procedures will occur at site visits to each participating health center. Site visits will occur approximately every 2-4 months throughout the study.

Health center training

Training will occur at the first site visit after health center enrollment and after health center randomization.

Following enrollment, we will conduct TB guideline and registry training at the first pre-randomization site visit. Project staff, an NTLP/NTRL representative and the District TB Officer will conduct the training jointly using a standardized slide set that emphasizes Uganda NTLP guidelines/algorithms for TB diagnosis and treatment. In addition, project staff will assess the completeness of data recording in TB registers, and provide training as needed to improve completeness. Last, project staff will visit all GeneXpert testing hubs that receive samples from project sites, and provide refresher training on GeneXpert device maintenance and operation.

Following randomization, we will conduct a 2-day site visit to each intervention and control health center.

At intervention health centers, we will engage staff in a discussion of the site-specific laboratory, clinical, and pharmacy workflow reorientation required as part of the intervention TB diagnostic evaluation strategy, with an emphasis on prompt identification of patients suspected of TB, immediate referral to the laboratory for sputum collection, on-demand molecular testing of sputum, communication of results to clinicians, and same-day treatment initiation. A GeneXpert I device and solar panel will be installed (with both replaced by GeneXpert Omni once it becomes available in 2019), and laboratory staff will be trained on sputum collection, sputum testing using GeneXpert, recording of results, and device maintenance. Laboratory and clinical staff will be trained on interpretation of results. In addition, all staff will be introduced to performance feedback Report Cards and expectations for reviewing and acting on the Report Cards. A staff member will

be identified to record any change made in the “Comments” section of the Report Card. Lab and drug inventories will be reviewed to ensure there is an adequate supply of sputum collection cups, glass slides, staining reagents and TB drugs. Completeness of TB laboratory and treatment registers will be assessed, and re-training provided as needed.

At control health centers, we will re-emphasize messages related to TB guidelines presented during the initial pre-trial site visit, including guidelines and procedures related to referral of sputum samples for Xpert testing. Laboratory staff will receive refresher training on sputum collection and sputum smear microscopy, including proficiency testing using panel slides. Lab and drug inventories will be reviewed to ensure there is an adequate supply of sputum collection cups, glass slides, staining reagents and TB drugs. Completeness of TB laboratory and treatment registers will be assessed, and re-training provided as needed.

Patient-level data collection

Patient demographic and clinical (HIV and TB testing and treatment) data will be collected at all sites. At the first pre-trial site visit, project staff will photograph each page of the following data sources from January 2016 until the visit date: 1) NTLP Presumptive TB, Laboratory and Treatment registers and 2) Xpert laboratory requisition forms. Project staff will also train two health center staff (one primary, one backup) identified by the health center in-charge to take photos of these data sources every two weeks for the duration of the project (between October 1, 2018 and March 31, 2020) using a camera-enabled smartphone, and to upload the photos to a central secure server through REDCap mobile. Health center staff will be trained to delete photos from the phone after upload confirmation. In addition to these data sources, study staff will 1) Review pre-ART and ART registers during scheduled site visits to verify ART status for any HIV-positive patient missing ART information; 2) review additional data sources (*e.g.,* clinic registration logbook) as needed at scheduled site visits to attempt to track down key information missing from primary data sources; and 3) call MDR TB treatment centers to determine referral, additional testing and treatment status and outcomes for patients with RIF resistance identified through Xpert testing.

Symptom Screening

A questionnaire adapted from the routine TB case finding guide will be administered to document TB symptoms reported at each patient’s initial visit to the health clinic. This information will be used to assess the proportion of patients referred for TB testing who were eligible for testing based on reported symptoms. Surveys will be administered by phone two weeks after the patient’s initial clinic visit to avoid influencing the primary outcome. Surveys will also be administered in-person to patients who provide a sputum sample for TB testing. In-person surveys will take place during quarterly site visits. All patients who are at least 18 years old, meet the main study inclusion criteria, and enrolled over a specified three-month period will be eligible for participation in the symptom screening activity. Due to logistical feasibility, however, it is anticipated that only patients with a documented valid phone number and patients selected for in-person interviews during quarterly site visits will be included in this activity. Study staff will prioritize clinics with a smaller proportion of patients with phone numbers available for in-person interviews.

Patient surveys

We will administer cost and satisfaction with care surveys to 20 patients at each health center (N=400 total) in the pre-randomization period and to 40 patients at each health center (N=800) at least 6 months post-randomization (April-September 2019). The two surveys will involve the same patients when feasible, but administered to different patients if a patient does not wish to complete both.

The cost survey will collect data on direct and indirect costs (time to complete visit, lost wages, etc.) of TB diagnostic evaluation. Cost data collection will be based on the Tool to Estimate Patient Costs developed by the TB Coalition for Technical Assistance, which we have already adapted and used in Uganda.

The satisfaction with care survey will assess 18 items taken from the previously validated Patient Satisfaction Questionnaire (**PSQ**)[29] and adapted to the Ugandan context.[30] The items are constructed with a five-point Likert scale with categories ranging from "strongly disagree" to "strongly agree", and include both positively and negatively worded questions to minimize the potential bias that occurs from clustering of responses to one side of the scale. The questionnaire measures general satisfaction as well as four dimensions of care known to impact satisfaction: 1) Accessibility, availability, and convenience of health services (3 items); 2) Provider interpersonal skills (5 items); 3) Provider technical competence with respect to patient education, examination and counseling (4 items); and 4) Health facility environment, specifically with respect to the cleanliness and space in the waiting area (2 items).

Provider surveys

We will administer a survey to all health workers involved in providing TB diagnostic and/or treatment services at each study site in the pre-randomization period and again at quarterly site visits beginning at visits that occur at least 12 months after randomization (October 2019 – March 2020). The survey will collect data on four key constructs of the Theory of Planned Behavior (**TPB**) as related to adherence to TB evaluation guidelines (**Figure 1**): intention, beliefs/attitude, normative beliefs/subjective norms and self-efficacy/perceived behavioral control. Based on prior experience, we anticipate 5-10 health workers will be involved in TB diagnostic/treatment services at each health center (N=100-200 health workers total pre- and post-randomization).

Health system costing

Health system cost data will be collected from all 20 sites at least 12 months after randomization (October 2019 – March 2020). Cost data will be collected through detailed budgetary analysis, interviews of key staff members, review of logbooks and/or timesheets to record proportions of staff time devoted to various activities, and direct observation (*e.g.*, time-motion studies of at least 10 patients and 3 staff members per clinic).

Focus group discussions

Study staff will conduct focus group discussions (one at each intervention health center) with all eligible providers at least 12 months after randomization. The focus group discussions will assess fidelity and barriers to uptake of each intervention component. Focus group discussions will be conducted by trained and experienced staff, audio-recorded, and professionally transcribed.

Assessment of patient's vital status, diagnostic and treatment status, HIV status, and ART status

Study staff will call all eligible patients included in the study 6 months after their initial health center visit to assess vital status, diagnostic and treatment status, HIV status, and ART status (if the patient is HIV-infected). Staff will call phone numbers recorded in the lab register, Xpert referral form and/or TB treatment register. For participants who cannot be reached, staff will make up to two additional phone calls on successive days. If the participant or a family member cannot be reached by telephone, study staff will work with a community health worker from the health center at which the participant underwent TB testing to conduct a home visit (using the address recorded in the lab register, Xpert referral form and/or TB treatment register). In addition to tracing efforts, staff will review TB treatment register data to ascertain vital status for participants who cannot be reached by phone but are known to have initiated TB treatment.

Provider interviews

Study staff will conduct in depth semi-structured interviews with 20-30 (2-3 per site) eligible providers to understand reasons for variability in uptake and effectiveness of the intervention strategy after completion of the trial. Health workers will be sampled purposively (*i.e.,* from low- and high-performing sites). Interviews will be conducted by trained and experienced staff, audio-recorded, and professionally transcribed.

Figure 3. Site visit activities

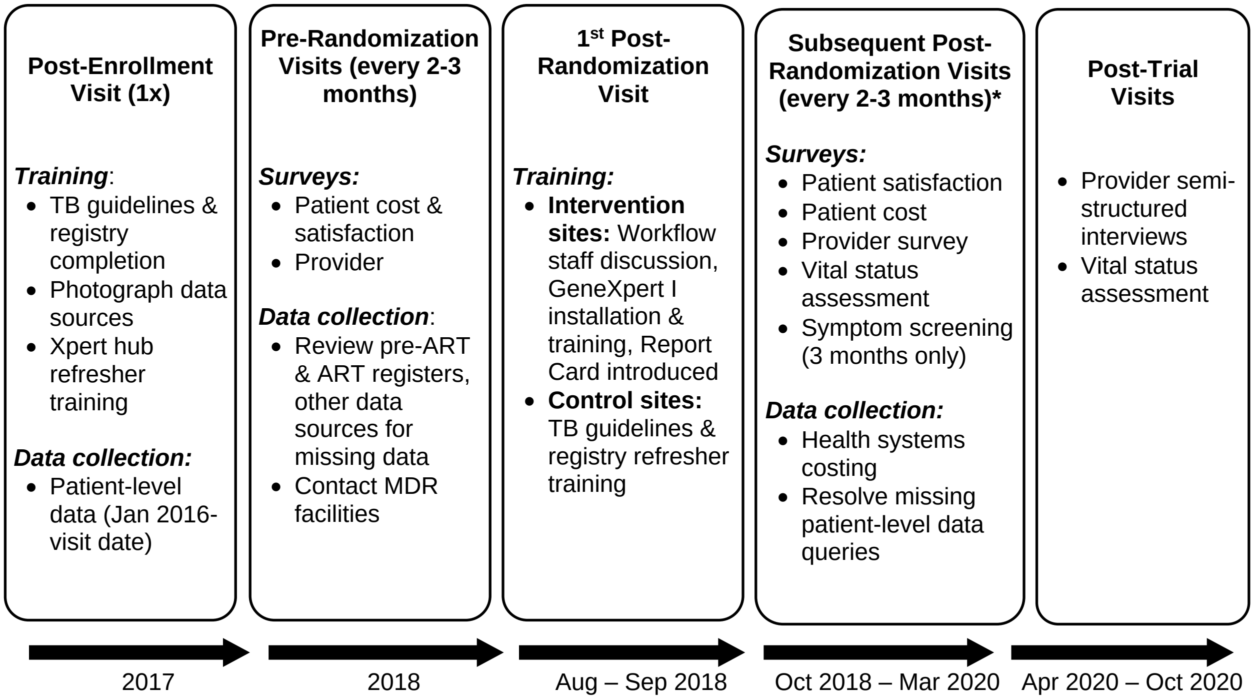

* At intervention health centers, GeneXpert I will be replaced with GeneXpert Omni once Omni is available at a regularly scheduled post-randomization visit. Lab staff will receive training on use of GeneXpert Omni and the solar panel used to power the GeneXpert I instrument will be removed.

Outcomes

Definitions

- ***Number enrolled***: Number of eligible patients identified over a defined enrolment period through review of the NTLP Presumptive TB register, NTLP Laboratory register, GeneXpert laboratory requisition forms, GxAlert database and NTLP Treatment register at each study site (includes patients treated for TB without undergoing any sputum testing).

- ***Date enrolled***: Earliest date recorded in the NTLP Presumptive TB register, NTLP Laboratory register, GeneXpert laboratory requisition form, GxAlert database, or NTLP Treatment register. Records will be prospectively reviewed using name, sex, and age to identify patients presenting multiple times over the study period. Visits occurring within six months of the initial presentation will be considered as part of the same episode.

- ***Number referred for testing***: Number of eligible patients identified through review of the NTLP Presumptive TB register, NTLP Laboratory register, GeneXpert laboratory requisition forms, and GxAlert database at each study site.

- ***Number tested***: Number of eligible patients with any smear or Xpert result entered into the NTLP Presumptive TB register, NTLP Laboratory register, GeneXpert laboratory requisition forms, NTLP treatment register, or GxAlert database at each study site.

- **Number completing testing**: Number of eligible patients with one valid Xpert result plus number of eligible patients with a valid smear result entered into the NTLP Presumptive TB register, NTLP Laboratory register, GeneXpert laboratory requisition forms, GxAlert database, or NTLP treatment register. Definitions of valid results are as follows:

    - A positive Xpert result with a semi-quantitative result of high, medium, low, or very low for all patients; a positive Xpert Ultra result with a semi-quantitative result of trace for HIV-positive patients; or second positive Xpert Ultra result if the initial result is trace-positive for HIV-negative patients;
    - A negative Xpert or Xpert Ultra result for all patients;[31]
    - One positive or two negative smear results for HIV-negative/HIV status unknown patients.

- ***Number diagnosed***: Number of eligible patients with microbiologically-confirmed TB via a positive smear and/or Xpert test result entered into the NTLP Presumptive TB register, NTLP Laboratory register, GeneXpert laboratory requisition forms, NTLP treatment register, and/or GxAlert database within 6 months of *date enrolled*.

- ***Number with suspected RIF resistant TB***: Number of eligible patients with RIF resistance identified by Xpert testing entered into the GxAlert database, GeneXpert laboratory requisition forms, and/or NTLP Laboratory registers within 6 months of *date enrolled*.

- ***Number with confirmed RIF resistant TB***: Number of eligible patients with RIF resistance identified by Xpert testing entered into the GxAlert database, GeneXpert laboratory requisition forms, and/or NTLP Laboratory registers, and RIF resistance confirmed by culture-based DST or a second molecular assay as determined by review of Lab Register at MDR treatment center within 6 months of *date enrolled*.

- ***Number treated***: Number of eligible patients entered into the NTLP Treatment register as having started Category I or II regimen, or started on MDR treatment at the MDR treatment center as determined by review of Treatment Register at MDR treatment center within 6 months of *date enrolled.*

- ***Number completing treatment***: *Number treated* and with a treatment outcome of cured or completed entered into the NTLP Treatment register or indicated by MDR treatment center staff through direct follow up.

- ***Number died***: *Number treated* and with a treatment outcome of died entered into the NTLP Treatment register within 6 months of *date enrolled*, or number eligible with outcome entered as died on direct follow-up form.

- ***Time-to-diagnosis***: Number of days from *date enrolled* to earliest date of positive smear or Xpert result recorded in NTLP Laboratory register, GeneXpert laboratory requisition form, NTLP treatment register, or GxAlert database within 6 months of *date enrolled*.

- ***Time-to-treatment***: Number of days from *date enrolled* to treatment start date entered into NTLP Treatment register if treatment start date is within 6 months of *date enrolled.*

I. Primary Outcome (Effectiveness)

Outcome	Numerator	Denominator
Number treated for microbiologically-confirmed TB within two weeks of referral for sputum-based testing	Number diagnosed and time-to-treatment within 14 days	None

II. Secondary Outcomes (Effectiveness)

	**A. Testing**		
**Outcome**		**Numerator**	**Denominator**
Number referred for TB testing		Number referred for testing	None
Proportion completing testing		Number completing testing	Number referred for testing
	**B. Diagnosis**		
**Outcome**		**Numerator**	**Denominator**
Number diagnosed with microbiologically-confirmed TB*		Number diagnosed	None
Proportion diagnosed with microbiologically-confirmed TB*		Number diagnosed	Number referred for testing
Number suspected/diagnosed with RIF-resistant TB*		Number with suspected/confirmed RIF resistant TB	None
Proportion suspected/diagnosed with RIF-resistant TB*		Number with suspected/confirmed RIF resistant TB	Number referred for testing

Time to microbiologically-confirmed TB		Time-to-diagnosis if microbiologically-confirmed TB	None
	**C. Treatment**		
**Outcome**		**Numerator**	**Denominator**
Number treated for TB*		Number treated	None
Proportion treated for TB*		Number treated	Number enrolled
Number treated for microbiologically-confirmed TB*		Number diagnosed AND treated	None
Proportion treated for microbiologically-confirmed TB*		Number diagnosed AND treated	Number referred for testing
Proportion with microbiologically-confirmed TB treated*		Number diagnosed AND treated	Number diagnosed
Time-to-treatment of microbiologically-confirmed TB		Time-to-treatment if microbiologically-confirmed TB and treated	None
	**D. Follow-up**		
**Outcome**		**Numerator**	**Denominator**
Number with microbiologically-confirmed TB completing treatment		Number diagnosed AND completing treatment	None
Proportion with microbiologically-confirmed TB completing treatment		Number diagnosed AND completing treatment	Number diagnosed AND treated
Number who died within 6 months		Number died	None
Proportion who died within 6 months**		Number died	Number enrolled

** Outcome will be assessed within 1 day and within 14 days of initial sputum submission. One day was chosen because the intervention focuses on same-day diagnosis and treatment. 14 days was chosen because the diagnostic process could take 7-10 days in the control arm depending on the frequency of sample transport to Xpert testing sites.*
***Treatment outcomes will be assessed at 7, 9, 12, and 24-month post-treatment initiation intervals determined by drug regimen.*

III. Implementation Outcomes

**Comparison across arms**	
**Outcome**	**Definition**
Patient costs<br>a) Total costs	 a) Sum of all patient-reported costs on cost questionnaire

b) Total direct costs	b) Total costs paid by the patient (i.e., excluding lost wages or other lost opportunities to earn income)
c) Total indirect costs	c) Patient-reported lost wages or other lost opportunities to earn income
Patient satisfaction with care survey:	
a) General satisfaction with care	a) Sum of score on 3 question items (Q16, 17 and 18)
b) Convenience of services	b) Sum of score on 3 question items (Q1, 2 and 3)
c) Health facility environment	c) Sum of score on 2 question items (Q4 and 5)
d) Provider interpersonal skills	d) Sum of score on 5 question items (Q7-11)
e) Provider technical competence	e) Sum of score on 4 question items (Q12-15)
Provider survey:	
a) Intention	a) Score on 1 question item (Q4)
b) Attitudes/beliefs	b) Sum of score on 3 question items (Q5a, 6a, and 6b)
c) Social norms/expectations	c) Sum of score on 2 question items (Q5b, 6c and 6d)
d) Self-efficacy/behavioral control	d) Sum of score on 5 question items (Q5c, 6e and 6f)

**Intervention arm only**		
**Outcome**	**Numerator**	**Denominator**
Process metrics:		
a) GeneXpert device non-operation days	a) GeneXpert device non-operation days[1]	a) Total number of lab operation days[1]
b) Proportion tested by GeneXpert:	b) Number with GeneXpert result date	b) Number tested by GeneXpert [2]
1. On same day as initial sputum submission	1. Same as initial sputum submission date[2]	
2. By next day after initial sputum submission	2. By next day after initial sputum submission date[2]	
c) Proportion with indeterminate results	c) Number with coded error, invalid or no test result[2]	c) Number of initial GeneXpert tests done[2]
d) Proportion with coded error result	d) Number with coded error result[2]	d) Number of initial GeneXpert tests done[2]
e) Proportion with invalid result	e) Number with invalid result[2]	e) Number of initial GeneXpert tests done[2]
f) Proportion with “no test” result	f) Number with “no test” result[2]	f) Number of initial GeneXpert tests done[2]
g) Proportion with GeneXpert test repeated if initial result indeterminate	g) Number with second GeneXpert result[2]	g) Number with initial indeterminate GeneXpert result[2]
	h) # treated on same day as initial health center visit[3]	h) # Xpert-positive[2]

h) Proportion treated on same-day if Xpert-positive<br>i) Proportion of report cards reviewed at staff meetings	i) Number of Report Cards with Comments section filled[4]	i) Total number of report cards issues to health centers[3]
Variation in and barriers to intervention strategy uptake	Themes emerging from focus group discussions, semi-structured interviews with health center staff	

Source of data: [1] Health center laboratory log; [2] GeneXpert Software; [3] Treatment register; [4] Performance feedback report card

IV. Cost-effectiveness/Modeling Outcomes

Outcome	Definition	Source
Incremental cost per DALY averted	(Cost in intervention – cost in control)/(DALYs averted by intervention – DALYs averted in control) from societal perspective	Markov-based decision models
Incremental health system cost per DALY averted	As above, but from health system/provider perspective	
Incremental patient cost per DALY averted	As above, but from patient perspective	
Projected reduction in TB incidence over 10 years	(Projected incidence in control model – projected incidence in intervention model)/(Projected incidence in control model), cumulative over 10 years	Transmission models
Projected reduction in TB mortality over 10 years	(Projected mortality in control model – projected mortality in intervention model)/(Projected mortality in control model), cumulative over 10 years	

Data Management

Dr. Fielding (statistician) will oversee data management in conjunction with UCSF- and Uganda-based study coordinators using the NIH-recommended Research Electronic Data Capture (**REDCap**) software, password-protected and accessible only to research staff. All data collection forms will be entered into standardized REDCap forms, with validation of data using range and consistency checks. Quality control procedures will include review of all study data collection forms for completeness and accuracy prior to data capture. Study staff will begin data capture by compiling photos from each individual health center for each data collection period. Using patient name, age, and sex, the staff will create one unique REDCap database entry (*i.e.* record) per patient by tracking patients across data sources in the following order: 1) Presumptive TB Register; 2) Laboratory Register; 3) Xpert Referral form; and 4) Treatment Register. If the patient cannot be located in a subsequent data source, the staff will check the next, until all data sources are consulted. If a patient does not meet inclusion criteria for analysis due to examination type (outreach, transferred in, follow up, or other), the staff will not collect data beyond the patient's

name, sex, age, and exam type from the Laboratory Register. Once patients have been matched across all data sources, the staff will run a series of reports in REDCap to verify the completeness and accuracy of key variables impacting patient eligibility and/or study outcomes: age, sex, HIV status, examination type, smear and Xpert test results, outcomes dates (sputum collection, test results, start and end of treatment, treatment outcome), and treatment status. Study staff will review the original data photographs to verify the information is missing, and they will compile a list of follow up items for each health center. Research staff will phone health center staff after each 2-week period of data is extracted to resolve missing information and clarify any discrepancies or uncertainties in matching. Once the health center reviews the missing and/or inaccurate data, the study staff will update REDCap. The UCSF study coordinator will visit Uganda 2-3 times a year and review a random sample of forms and primary data sources for quality assurance.

Statistical Analysis

Aim 1

A detailed Statistical Analysis Plan will be developed. Briefly, we will calculate and compare all effectiveness outcomes for the two trial arms using an intention-to-treat analysis. We will use descriptive statistics and 95% confidence intervals to summarize the yield, efficiency, and speed outcomes for the two arms by site. To assess the intervention effect on outcomes, we will use methods appropriate for randomization of a small number of clusters and accounting for the stratified design.[26] A cluster-level analysis, giving each cluster equal weight, will be conducted to calculate unadjusted ratio and difference effect measures, and their associated 95% confidence intervals. Adjusted effect estimates will also be calculated taking into account any imbalance of important factors at baseline by study arm. Pre-specified subgroup analyses (*e.g.,* gender and HIV status) will be conducted for the primary outcome. A detailed statistical analysis plan will document methods used for the analysis of primary and secondary outcomes, and document pre-specified sub-group analyses.

Aim 2

1) Descriptive analyses – We will report process metrics on a monthly basis to assess adoption and maintenance of each intervention component overall, within key patient sub-groups, and at individual sites. We will report median and change between pre- and post-randomization assessments in **A)** patient costs; **B)** patient satisfaction with care; and **C)** provider TPB construct scores at control and intervention sites.

2) Comparative analyses – To identify patient-, provider-, and/or clinic-level factors independently associated with adoption and maintenance of intervention components, we will develop linear or logistic regression models, taking into account the clustered design (for example, robust standard errors). To compare by study arm the change from baseline (pre-randomization) to post-intervention in **A)** patient cost; **B)** patient satisfaction with care; and **C)** TPB construct scores, we will use a cluster-level analysis, similar to methods described for the primary outcome under Aim 1. The analysis will take into account the stratified design and an adjusted analysis will also be conducted taking into account other baseline imbalances by study arm.

3) Qualitative analyses – De-identified focus group or interview transcripts will be uploaded to the qualitative data analysis software Dedoose (SocioCultural Research Consultants, USA). Thematic interpretation[32-34] will include collaborative development of a coding framework and detailed coding of transcripts using Dedoose. Coded transcripts will be sorted to identify thematic groupings. The thematic groupings will be reviewed to identify emergent themes within each domain of the coding framework and quotes that best represent each domain. Thematic interpretation will focus on individual, social and structural factors associated with successful or unsuccessful adoption and/or maintenance of the intervention components at different sites.

Aim 3

Cost-effectiveness -- Our primary outcome will be the incremental cost-effectiveness of the intervention strategy from a societal perspective, measured as the cost per disability-adjusted life year (DALY) averted relative to standard TB evaluation. Secondary outcomes will include 1) the incremental cost of introducing and maintaining the intervention strategy (a measure of affordability for health systems) and 2) the incremental patient cost per diagnostic evaluation and per treatment initiated (a measure of affordability and access for patients). For all outcomes, we will report stratified results for key populations including women and people living with HIV. To assess cost-effectiveness, we will use the primary effectiveness data from Aim 1 and relevant literature estimates (e.g., clinical outcomes among those initiating treatment) to construct a Markov model including states for undiagnosed TB but seeking care, undiagnosed TB not seeking care (e.g., because initial diagnostic evaluation was too expensive), treated TB, self-resolved TB, and death. States will be subdivided according to smear and Xpert status (smear-positive, smear-negative/Xpert-positive, and smear/Xpert-negative) and HIV status (positive and negative, on/off ART, with CD4 strata). Data to inform transition probabilities and health utilities will come from study data where feasible (including specific questions of patients to ascertain probabilities such as future care-seeking if diagnoses are missed), and the literature where unavailable. We will follow international conventions for all procedures including economic costing, discounting, and reporting. We will conduct one-way sensitivity analyses across all model parameters, multi-way sensitivity analyses for those parameters found to be most influential, and a probabilistic uncertainty analysis in which all parameters are varied simultaneously using Latin Hypercube Sampling.

Population-Level Epidemiological Impact – We will construct a compartmental epidemic model of TB in Uganda to evaluate the potential impact of scaling up the intervention strategy across a representative district in Uganda. Using our team's prior models of TB diagnostics in India[35] and sub-Saharan Africa as a starting point, we will construct a population model that includes structure both for TB natural history (e.g., latent, subclinical, pre-diagnostic, diagnosis-seeking, treated)[36] and steps in the diagnostic cascade (e.g., pursuing diagnosis, diagnosed but not treated, treated).[35, 37] We will include structure for both HIV and MDR TB, and will link this model's structure to that of the Markov model constructed for cost-effectiveness analysis above (for purposes of explicitly estimating the importance of transmission to considerations of cost-effectiveness). We will fit the model to epidemiological data from a selected representative district in Uganda according to its TB incidence, prevalence, and mortality, as well as additional factors including prevalence of HIV and of MDR-TB. This model will project TB incidence and mortality over a primary time-frame of 10 years under two alternative scenarios: 1) standard TB diagnostic

evaluation and 2) streamlined TB diagnostic evaluation using the intervention strategy. In a secondary analysis, we will incorporate economic data as described above, comparing cost-effectiveness measured under a dynamic (epidemic-economic) framework to that of the Markov model. The dynamic transmission model has the advantage of incorporating transmission dynamics at the population level, but requires more assumptions (*e.g.*, homogeneous mixing in the source population). By comparing results from Markov and transmission modeling, we will be able to assess: 1) the relative contribution of population-level transmission to the overall effectiveness and cost-effectiveness of different TB diagnostic evaluation strategies over time and 2) the relative influence of given model parameters on cost- effectiveness under a cohort-based versus transmission-based evaluation model.

Sample Size Considerations

Aim 1

Original detectable effect size estimates: The study is based on the health clinic being the unit of randomization and aims to demonstrate the superiority of the intervention arm. The sample size calculation uses formulae appropriate for cluster-randomized trials with a parallel design and stratified and/or restricted randomization, including the addition of one extra cluster per arm to allow for the loss of degrees of freedom due to stratification to obtain conservative estimates.[26] The original detectable effect size estimates were based on the outcome being the proportion of patients treated for microbiologically-confirmed TB within two weeks of referral for sputum-based testing. A type I error of 5% and power of 90% was assumed. Pre-randomization data collected from January to December 2017 from the 20 selected trial sites in Uganda suggests the average proportion of patients referred for TB evaluation who initiate treatment for active TB within two weeks is 6.7%, and the coefficient of variation (k) between clusters is 0.36, with a harmonic mean of 268 patients enrolled at each health center cluster during the 18-month enrolment period. Based on these assumptions and assuming a type I error of 5%, we will have 90% power to detect a 6% or greater absolute increase in the outcome proportion in the intervention arm with a trial duration of 18 months (see **Table**). Table 1 also shows detectable absolute effect sizes with varying power (80%, 85%, 90%) during various trial time intervals and respective average cluster size estimates.

Average cluster size	Effect size (10 clusters per arm, *k*=0.36)		
	80% Power	85% Power	90% Power
178 (*12 months*)	5.9%	6.5%	7.4%
223 (*15 months)*	5.7%	6.3%	7.1%
268 (*18 months*)	5.5%	6.1%	6.9%
312 (*21 months*)	5.4%	6.0%	6.7%
357 (*24 months*)	5.3%	5.9%	6.6%

Revised detectable effect size estimates: The revised detectable effect size estimates are for the outcome being the number of patients treated for microbiologically-confirmed TB within two weeks of referral for sputum-based testing. We first used 10 months of pre-intervention period data across all 20 clinics to assess 1) the geometric mean number of patients diagnosed and treated

for TB within 14 days (new primary outcome), the natural log (ln) of the geometric mean and 3) the standard deviation of the natural log at control sites, intervention sites and overall.. For the pre-intervention period, the intervention clinics have a lower geometric mean number of clinic attendees who were diagnosed with TB and started on TB treatment within 14 days compared with the control arm:

	Geometric mean	Mean of ln outcome	SD of ln outcome
Control	14.70	2.69	0.23
Intervention	6.49	1.87	0.32

We then estimated the detectable effect size (expressed as a geometric mean ratio). The calculations assume the same parameters as for the original primary outcome (10 clinics/arm, 18-month trial duration to achieve geometric mean of 286 patients/cluster in control and intervention arms). Table 1 below shows the detectable effect size assuming within-arm SD of ln outcome of 0.2 to 0.3 and power of 80-90%. The detectable effect sizes shown below are conservative (calculations do not take into account the baseline differences between arms in the new primary outcome and are based on 10-months rather than full 24-months of pre-intervention data). Final calculations will be repeated once baseline data is fully entered and available for analysis.

Table 3: Detectable effect sizes

SD of ln outcome	Geometric Mean Ratio of the new primary outcome (intervention vs control)	
	80% power	90% power
0.2	1.30	1.36
0.3	1.49	1.58

We believe a GMR of 1.30-1.58 is a reasonable detectable effect size. Xpert MTB/RIF is twice as sensitive as smear microscopy (double the number of confirmed TB cases) and we expect onsite testing to reduce pre-treatment loss to follow-up by at least half (from 30% to <15%).

Aim 2

The sample size for Aim 2 analyses is either fixed (process metrics; provider surveys and focus groups) by parameters of the clinical trial or based on feasibility considerations (patient surveys and provider in depth interviews). For quantitative analyses, the sample size is sufficiently large (data on 3400 patients for process metrics analyses, 800 patient surveys, and 100-200 provider surveys) to enable multivariable analysis to identify factors associated with intervention adoption and maintenance.

Aim 3

As Aim 3 is primarily a modeling aim, sample size calculations are not applicable. Sample size considerations for the cost data (i.e., number of direct observations through time and motion studies, number of patients completing the cost questionnaires) are based on feasibility considerations and desire to estimate model parameters to sufficient levels of precision, as described above for Aim 2.

Ethical Considerations

Potential risks to participants

There are minimal risks to participants in this study. The primary risk to patients undergoing TB diagnostic evaluation during the study period is the potential for loss of confidentiality and stigma should their personal health information, including HIV or TB status, be disclosed. Patients participating in surveys also have the potential of sensitive information regarding their income being disclosed. Finally, the primary research risks for health workers related to the surveys and qualitative studies are punitive actions by the employer in response to the information they provide for research.

Protection against risk

The trial will be submitted for approval to the Research Ethics Committees of the University of California San Francisco and Makerere University College of Health Sciences, and to the Uganda National Council for Science and Technology. It is registered with the U.S. National Institutes of Health's ClinicalTrials.gov and the Pan African Clinical Trials Registry (PACTR) as a Phase 4 clinical trial. All study staff will be required to have completed Good Clinical Practice (GCP) training.

To minimize the potential for loss of confidentiality, all patient-identifiable data will be stored in locked or password protected areas accessible only to study personnel. Patient names will be used to match patient records across NTLP Laboratory and Treatment registers, but will not be included in the password-protected, electronic study database. Primary data collection forms used when matching records across data sources will be destroyed once entry of data into the electronic study database is completed.

To minimize risks to autonomy for patients and providers who participate in surveys, research staff will be carefully trained in how to administer the consent form to the individuals in the different target populations, with attention given to the background and principles of research ethics.

The Directors of participating health centers will be asked to sign a Memorandum of Understanding agreeing that their health center participate in the trial and agreeing not to introduce any new TB evaluation interventions during the time period of the trial without informing trial staff. Individual patients evaluated for TB at participating health centers will not be consented because the trial meets the requirements to qualify for waiver of informed consent under U.S. Department of Health and Human Services (DHHS) Regulation 46.116 (d): 1) No data or samples will be collected specifically for research purposes; 2) Patients will receive the same or higher quality of care; 3) It is not practical for health workers to obtain informed consent during the process of delivering routine clinical care; and 4) All pertinent TB testing results will be communicated to patients via routine or enhanced processes of care. Verbal informed consent using a script will be obtained from patients who participate in surveys. Written informed consent will be obtained from providers who participate in surveys, direct observation (*i.e.,* time-motion) studies and/or focus group discussions/interviews. The consent forms, which will be approved by institutional review boards (IRB) in the U.S. and Uganda, will be translated from English into the

local languages, and back-translated into English as required by IRBs to be sure that no significant language or concepts are lost in translation.

Data protection

All patient-identifiable data will be stored in locked or password-protected cabinets or databases accessible only to study personnel. Patient names will be used to match patient records across NTLP Laboratory and Treatment registers but will not be included in the password-protected, electronic study database. Primary data collection forms and images used when matching records across data sources will be destroyed once entry of data into the electronic study database is completed.

Potential benefits of the study to participants and society

Patients undergoing TB diagnostic evaluation at participating health centers may benefit from the study through potential enhanced diagnosis and treatment of TB, either because of training (control health centers) or training plus other interventions (intervention health centers). Earlier diagnosis and treatment of TB may lead to improved patient outcomes and reduced disease transmission in patients' communities.

Patients and providers who participate in surveys, in depth interviews or focus group discussions will receive sodas and/or lunch to compensate for their time. Otherwise, they will not directly benefit from participating in these research activities.

Potential benefits to society include identification of strategies to improve TB case finding and to decrease TB incidence and prevalence in low-income, high-burden countries. If successful, the proposed intervention could potentially be scaled up to improve TB care in similar settings.

Dissemination

The trial results will be communicated to stakeholders through dissemination meetings and to participating health centers using language-appropriate information sheets. Investigators will present results at relevant conferences, and submit manuscript(s) to peer-reviewed journals. Public access to the participant-level dataset of main trial results and statistical code will be made available.

Trial governance

Because of the low-risk nature of the research, the Principal Investigator will be responsible for monitoring the data, assuring protocol compliance, and conducting safety reviews on a quarterly basis. An independent Trial Steering Committee (TSC) will meet approximately every 6 months and as needed. Prior to each meeting, the Principal Investigator will submit a progress report, including recommendations on whether the project should continue unchanged, require modification/amendment, or close to enrollment to an independent Trial Steering Committee (TSC). All major modifications (e.g., study design, sample size, study termination or suspension), will be approved by the TSC and ethics committees.

Abbreviations

AFB	Acid-Fast Bacilli
ART	Anti-retroviral therapy
CFR	Code of Federal Regulations
CRT	Cluster-randomized trial
DALY	Disability-adjusted life year
DHHS	Department of Health & Human Services
DMC	Data Monitoring Committee
EQA	External quality assurance
FDA	U.S. Food and Drug Administration
GCP	Good Clinical Practice
ICH	International Council for Harmonisation of Technical Requirements for Pharmaceuticals for Human Use
IRB	Institutional review board
ISTC	International Standards for TB Care
KAP	Knowledge, attitudes, and practices
LED	Light-emitting diode
MDR-TB	Multi-drug-resistant tuberculosis
MOU	Memorandum of Understanding
MUREC	Makerere University Research Ethics Committee
NIH	National Institutes of Health
NHLBI	National Heart, Lung, and Blood Institute
NTLP	National Tuberculosis and Leprosy Program
NTP	National TB Program
NTRL	National TB Reference Laboratory
PRECEDE	Predisposing, Reinforcing, and Enabling Constructs in Educational/Environmental Diagnosis and Evaluation
PSQ	Patient Satisfaction Questionnaire
RE-AIM	Reach Effectiveness Adoption Implementation Maintenance
REDCap	Research Electronic Data Capture
TB	Tuberculosis
TPB	Theory of Planned Behavior
TSC	Trial Steering Committee
UCSF	University of California San Francisco
UCSF CHR	UCSF Committee on Human Research
UNCST	Uganda National Council for Science and Technology
WHO	World Health Organization
XPEL TB	GeneXpert Performance Evaluation for Linkage to Tuberculosis Care
ZN	Ziehl–Neelsen

Competing interests

The investigators declare that they have no competing interests.

Trial Steering Committee

Jerry Friedland (Yale University)
Noah Kiwanuka (Makerere University)
Madhukar Pai (McGill University)
Andrew Ramsay (Division of Infection and Global Health, St Andrews University Medical School, Scotland)
Grant Theron (Stellenbosch University)

References

1. WHO. Global tuberculosis report 2016. Geneva, Switzerland: World Health Organization; 2016.
2. MacPherson P, Houben RM, Glynn JR, Corbett EL, Kranzer K. Pre-treatment loss to follow-up in tuberculosis patients in low- and lower-middle-income countries and high-burden countries: a systematic review and meta-analysis. Bull World Health Organ. 2014;92(2):126-38. doi: 10.2471/BLT.13.124800. PubMed PMID: 24623906; PMCID: PMC3949536.
3. Cattamanchi A, Dowdy DW, Davis JL, Worodria W, Yoo S, Joloba M, Matovu J, Hopewell PC, Huang L. Sensitivity of direct versus concentrated sputum smear microscopy in HIV-infected patients suspected of having pulmonary tuberculosis. BMC Infect Dis. 2009;9:53. Epub 2009/05/08. doi: 1471-2334-9-53 [pii]
10.1186/1471-2334-9-53. PubMed PMID: 19419537; PMCID: 2690598.
4. Chihota VN, Ginindza S, McCarthy K, Grant AD, Churchyard G, Fielding K. Missed Opportunities for TB Investigation in Primary Care Clinics in South Africa: Experience from the XTEND Trial. PloS one. 2015;10(9):e0138149. doi: 10.1371/journal.pone.0138149. PubMed PMID: 26383102; PMCID: PMC4575203.
5. Davis J, Katamba A, Vasquez J, Crawford E, Sserwanga A, Kakeeto S, Kizito F, Dorsey G, den Boon S, Vittinghoff E, Huang L, Adatu F, Kamya MR, Hopewell PC, Cattamanchi A. Evaluating tuberculosis case detection via real-time monitoring of tuberculosis diagnostic services. American journal of respiratory and critical care medicine. 2011;184(3):362-7. doi: 10.1164/rccm.201012-1984OC. PubMed PMID: 21471088; PMCID: 3175538.
6. Aspler A, Menzies D, Oxlade O, Banda J, Mwenge L, Godfrey-Faussett P, Ayles H. Cost of tuberculosis diagnosis and treatment from the patient perspective in Lusaka, Zambia. The international journal of tuberculosis and lung disease : the official journal of the International Union against Tuberculosis and Lung Disease. 2008;12(8):928-35. PubMed PMID: 18647453.
7. Chandrasekaran V, Ramachandran R, Cunningham J, Balasubramaniun R, Thomas A, Sudha G, Jegannatha Rao K, Perkins M, PR N. Factors leading to tuberculosis diagnostic drop-out and delayed treatment initiation in Chennai, India. The international journal of tuberculosis and lung disease : the official journal of the International Union against Tuberculosis and Lung Disease. 2005;9(S1):172.
8. Kemp JR, Mann G, Simwaka BN, Salaniponi FM, Squire SB. Can Malawi's poor afford free tuberculosis services? Patient and household costs associated with a tuberculosis diagnosis in Lilongwe. Bull World Health Organ. 2007;85(8):580-5. PubMed PMID: 17768515; PMCID: 2636388.
9. Shete PB, Haguma P, Miller CR, Ochom E, Ayakaka I, Davis JL, Dowdy DW, Hopewell P, Katamba A, Cattamanchi A. Pathways and costs of care for patients with tuberculosis symptoms in rural Uganda. The international journal of tuberculosis and lung disease : the official journal of the International Union against Tuberculosis and Lung Disease. 2015;19(8):912-7. doi: 10.5588/ijtld.14.0166. PubMed PMID: 26162356.
10. Simwaka BN, Bello G, Banda H, Chimzizi R, Squire BS, Theobald SJ. The Malawi National Tuberculosis Programme: an equity analysis. Int J Equity Health. 2007;6:24. Epub 2008/01/01. doi: 1475-9276-6-24 [pii]
10.1186/1475-9276-6-24. PubMed PMID: 18163918; PMCID: 2253525.
11. Botha E, den Boon S, Lawrence KA, Reuter H, Verver S, Lombard CJ, Dye C, Enarson DA, Beyers N. From suspect to patient: tuberculosis diagnosis and treatment initiation in health facilities in South Africa. The international journal of tuberculosis and lung disease : the official journal of the International Union against Tuberculosis and Lung Disease. 2008;12(8):936-41. Epub 2008/07/24. PubMed PMID: 18647454.

12. Den Boon S, Semitala F, Cattamanchi A, Walter N, Worodria W, Joloba M, Huang L, Davis JL. Impact Of Patient Drop-out On The Effective Sensitivity Of Smear Microscopy Strategies. American journal of respiratory and critical care medicine. 2010;181:A2258.
13. Ouyang H, Chepote F, Gilman RH, Moore DA. Failure to complete the TB diagnostic algorithm in urban Peru: a study of contributing factors. Trop Doct. 2005;35(2):120-1. Epub 2005/06/23. doi: 10.1258/0049475054037002. PubMed PMID: 15970047.
14. WHO. WHO monitoring of Xpert MTB/RIF roll-out. Available at: http://www.who.int/tb/areas-of-work/laboratory/mtb-rif-rollout/en/ [cited 2015 January 15].
15. Steingart KR, Schiller I, Horne DJ, Pai M, Boehme CC, Dendukuri N. Xpert(R) MTB/RIF assay for pulmonary tuberculosis and rifampicin resistance in adults. The Cochrane database of systematic reviews. 2014;1:CD009593. doi: 10.1002/14651858.CD009593.pub3. PubMed PMID: 24448973.
16. Dowdy DW, Cattamanchi A, Steingart KR, Pai M. Is scale-up worth it? Challenges in economic analysis of diagnostic tests for tuberculosis. PLoS medicine. 2011;8(7):e1001063. doi: 10.1371/journal.pmed.1001063. PubMed PMID: 21814496; PMCID: 3144197.
17. Keeler E, Perkins MD, Small P, Hanson C, Reed S, Cunningham J, Aledort JE, Hillborne L, Rafael ME, Girosi F, Dye C. Reducing the global burden of tuberculosis: the contribution of improved diagnostics. Nature. 2006;444 Suppl 1:49-57. doi: 10.1038/nature05446. PubMed PMID: 17159894.
18. Churchyard GJ, on behalf of the Xtend study team. Xpert MTB/RIF vs microscopy as the first line TB test in South Africa: mortality, yield, initial loss to follow up and proportion treated. The Xtend Study. Conference on Retroviruses and Opportunistic Infections; Boston, USA: Available at: http://www.stoptb.org/wg/gli/assets/documents/M6/Churchyard%20-%20XTEND%20study.pdf; 2014.
19. Cepheid. GeneXpert Omni: The True Point of Care Molecular Diagnostic System: Cepheid Inc; 2015. Available from: http://www.cepheid.com/us/genexpert-omni.
20. Glasgow RE, Vogt TM, Boles SM. Evaluating the public health impact of health promotion interventions: the RE-AIM framework. American journal of public health. 1999;89(9):1322-7. PubMed PMID: 10474547; PMCID: 1508772.
21. Godin G, Belanger-Gravel A, Eccles M, Grimshaw J. Healthcare professionals' intentions and behaviours: a systematic review of studies based on social cognitive theories. Implementation science : IS. 2008;3:36. doi: 10.1186/1748-5908-3-36. PubMed PMID: 18631386; PMCID: 2507717.
22. Ajzen I. The theory of planned behavior. Organizational Behavior and Human Decision Processes. 1991;50:179-211.
23. Green LW, Krueter M. Health Program Planning - An Educational and Ecological Approach. 4th ed. Philadelphia, USA: McGraw-Hill; 2005.
24. Davis DA, Thomson MA, Oxman AD, Haynes RB. Evidence for the effectiveness of CME. A review of 50 randomized controlled trials. Jama. 1992;268(9):1111-7. PubMed PMID: 1501333.
25. Cattamanchi A, Miller C, Tapley A, Haguma P, Ochom E, Ackerman S, Davis JL, Katamba A, Handley MA. Health worker perspectives on barriers to delivery of routine tuberculosis diagnostic evaluation services in Uganda: A qualitative study to guide clinic-based interventions. . BMC Health Services Research. (in press).
26. Hayes RJ, Moulton LH. Cluster Randomized Trials. Boca Raton, Florida, USA: CRC Press; 2009.
27. Ivers NM, Halperin IJ, Barnsley J, Grimshaw JM, Shah BR, Tu K, Upshur R, Zwarenstein M. Allocation techniques for balance at baseline in cluster randomized trials: a methodological review. Trials. 2012;13:120. doi: 10.1186/1745-6215-13-120. PubMed PMID: 22853820; PMCID: 3503622.

28. Sismanidis C, Moulton LH, Ayles H, Fielding K, Schaap A, Beyers N, Bond G, Godfrey-Faussett P, Hayes R. Restricted randomization of ZAMSTAR: a 2 x 2 factorial cluster randomized trial. Clinical trials. 2008;5(4):316-27. doi: 10.1177/1740774508094747. PubMed PMID: 18697846.
29. Grogan S, Conner M, Norman P, Willits D, Porter I. Validation of a questionnaire measuring patient satisfaction with general practitioner services. Qual Health Care. 2000;9(4):210-5. PubMed PMID: 11101705; PMCID: PMC1743536.
30. Nabbuye-Sekandi J, Makumbi FE, Kasangaki A, Kizza IB, Tugumisirize J, Nshimye E, Mbabali S, Peters DH. Patient satisfaction with services in outpatient clinics at Mulago hospital, Uganda. Int J Qual Health C. 2011;23(5):516-23. doi: 10.1093/intqhc/mzr040. PubMed PMID: WOS:000294810300004.
31. World Health Organization. WHO Meeting Report of a Technical Expert Consultation: Non-inferiority analysis of Xpert MTB/RIF Ultra compared to Xpert MTB/RIF Geneva: World Health Organization; 2017. Available from: http://apps.who.int/iris/bitstream/10665/254792/1/WHO-HTM-TB-2017.04-eng.pdf.
32. Sandelowski M, Leeman J. Writing usable qualitative health research findings. Qualitative health research. 2012;22(10):1404-13. doi: 10.1177/1049732312450368. PubMed PMID: 22745362.
33. Sandelowski MJ. Justifying qualitative research. Research in nursing & health. 2008;31(3):193-5. doi: 10.1002/nur.20272. PubMed PMID: 18288640.
34. Voils CI, Sandelowski M, Barroso J, Hasselblad V. Making Sense of Qualitative and Quantitative Findings in Mixed Research Synthesis Studies. Field methods. 2008;20(1):3-25. doi: 10.1177/1525822X07307463. PubMed PMID: 18677415; PMCID: 2493048.
35. Salje H, Andrews JR, Deo S, Satyanarayana S, Sun AY, Pai M, Dowdy DW. The importance of implementation strategy in scaling up Xpert MTB/RIF for diagnosis of tuberculosis in the Indian health-care system: a transmission model. PLoS medicine. 2014;11(7):e1001674. doi: 10.1371/journal.pmed.1001674. PubMed PMID: 25025235; PMCID: 4098913.
36. Dowdy DW, Basu S, Andrews JR. Is passive diagnosis enough? The impact of subclinical disease on diagnostic strategies for tuberculosis. American journal of respiratory and critical care medicine. 2013;187(5):543-51. doi: 10.1164/rccm.201207-1217OC. PubMed PMID: 23262515; PMCID: 3733406.
37. Sun AY, Denkinger CM, Dowdy DW. The impact of novel tests for tuberculosis depends on the diagnostic cascade. The European respiratory journal. 2014;44(5):1366-9. doi: 10.1183/09031936.00111014. PubMed PMID: 25186263; PMCID: 4254765.

Table of Amendments

XPEL TB Trial Protocol

Change	Date of change
**Change**	**Date of change**
**Overview**: We increased the sample size from 6500-7500 to 11,283	September 11, 2019
**Overview**: We reduced the study duration from 2 years to 18 months	September 11, 2019
**Timeline:** We added a timeline of activities	September 11, 2019
**Symptom Screening**: In response to the increase in patients referred for TB testing in the intervention arm, we documented TB symptoms reported at each patent's initial visit to the health clinic for a quarter to assess the proportion of patients referred for TB testing who are eligible for testing based on reported symptoms	September 11, 2019
**Outcomes**: We updated the primary outcome from a proportion to a count	September 11, 2019
**Sample Size Considerations**: We added text on the revised detectable effect size estimates given the change in the primary outcome from a proportion to a count	September 11, 2019
**Trial Steering Committee**: We updated affiliations for one of the trial steering committee members	September 11, 2019

Statistical Analysis Plan: XPEL TB

<table>
<tr><td>Full Title</td><td colspan="3">GeneXpert Performance Evaluation for Linkage to Tuberculosis Care: The XPEL TB Trial</td></tr>
<tr><td>Acronym</td><td colspan="3">XPEL TB</td></tr>
<tr><td rowspan="6">Document History</td><td>Version No.</td><td>Version Date</td><td>Description of Change</td></tr>
<tr><td>1.0</td><td>2019-10-11</td><td>Initial release</td></tr>
<tr><td></td><td></td><td></td></tr>
<tr><td></td><td></td><td></td></tr>
<tr><td></td><td></td><td></td></tr>
<tr><td></td><td></td><td></td></tr>
<tr><td>Trial Registration</td><td colspan="3">ClinicalTrials.gov (NCT03044158)
Pan African Clinical Trials Registry (PACTR201610001763265)</td></tr>
<tr><td>Principal Investigators</td><td colspan="3">Adithya Cattamanchi, Achilles Katamba</td></tr>
<tr><td>SAP Authors</td><td colspan="3">Katherine Fielding, Katherine Farr, Tania Reza</td></tr>
</table>

1. Introduction

1.1 Aim

To evaluate the performance of GeneXpert in improving tuberculosis diagnosis and treatment initiation rates using a multi-faceted strategy to streamline care (XPEL TB) relative to the prevailing standard-of-care in Uganda.

2. Background and Objectives

2.1 Specific Objectives

1. To compare patient outcomes at health centers randomized to intervention vs. standard-of-care TB diagnostic evaluation strategies.
    a. Intervention: Onsite molecular testing for TB with GeneXpert device + process redesign to facilitate same-day TB diagnosis and treatment + performance feedback
    b. Standard-of-care: Onsite ZN or LED fluorescence microscopy + hub-based GeneXpert testing per existing protocols
2. To identify processes and contextual factors that influence the effectiveness and fidelity of the intervention TB diagnostic evaluation strategy.
3. To compare the costs and epidemiological impact of intervention vs. standard-of-care TB diagnostic evaluation strategies.

2.2 Summary of aims

The study proposes to conduct a pragmatic, parallel cluster-randomized trial (CRT) with nested mixed methods, health economic and modelling studies to evaluate the effectiveness, implementation and costs of the intervention strategy relative to standard TB diagnostic evaluation at community health centers that are part of Xpert referral networks in Uganda. The effectiveness of the intervention strategy will be assessed using routine data collected as part of mandatory reporting to the Uganda NTLP on consecutive patients who present to participating health centers during the 18-month enrolment period and meet eligibility criteria. Selected patients and providers who provide informed consent will also be surveyed and/or interviewed to identify reasons for its success or failure across study sites (Aim 2), and to collect relevant cost data for cost-effectiveness analyses (Aim 3).

2.3 General comments

This statistical analysis plan covers all effectiveness, implementation, and cost-effectiveness/modelling outcomes. Trial results reporting will follow the CONSORT 2010 statement extension to cluster randomized trials.

This document summarizes analyses for the primary outcome of the number of patients treated for microbiologically-confirmed TB within 14 days of referral for sputum-based testing; and secondary outcomes for ***testing*** (number and proportion of patients completing TB testing), ***diagnosis*** (number and proportion diagnosed with microbiologically-confirmed TB, number and proportion diagnosed with RIF-resistant TB, time to microbiologically-confirmed TB), ***treatment*** (number and proportion treated for TB, time to treatment of microbiologically-confirmed TB), and ***follow-up*** (number and proportion with microbiologically-confirmed TB completing treatment, number and proportion who died within 6 months). These endpoints will be evaluated through review of TB registers and GeneXpert testing logs for all patients initiating TB evaluation at participating health centers.

This Statistical Analysis Plan is written in support of and is predominantly consistent with the full trial protocol; however, this analysis plan takes precedence. The analysis plan covers the primary and secondary study objectives.

3. Trial Summary

3.1 Trial design

The study is a parallel cluster-randomized trial (CRT) with two arms of equal number of clusters. The type of cluster used is *institutional health units*; in this case, health centers that provide TB microscopy services in Uganda in partnership with the National TB Program. Entire health centers will be the unit of randomization rather than individual participants to reduce contamination. The unit of analysis will be patients undergoing TB diagnostic evaluation during the 18-month enrolment period. The trial includes nested mixed methods and economic analyses to assess the implementation and cost-effectiveness of the intervention.

3.2 Study population

Eligibility Criteria

A. Site-level Inclusion Criteria
1. Use standard (multi-day) sputum smear microscopy as the primary method of TB diagnosis
2. Participate in NTP-sponsored external quality assurance (EQA) for sputum smear microscopy
3. Send samples to a district or regional hospital/health center for Xpert testing

B. Site-level Exclusion Criteria
1. Do not agree to be randomized to standard-of-care vs. intervention arms
2. Perform sputum smear examination on <150 patients per year (based on 2015 data)
3. Diagnose <15 smear-positive TB cases per year (based on 2015 data)

C. Patient-level Inclusion Criteria
1. Initiating TB evaluation at a study health center

D. Patient-level Exclusion Criteria
1. Have sputum collected for monitoring of response to anti-TB therapy (*i.e.*, recorded as Examination type=Follow Up in NTLP Laboratory Register);
2. Have sputum collected as part of active, community-based case finding (e.g., contact tracing, community outreach campaign) (*i.e.*, noted in the remarks in NTLP Laboratory Register);
3. Referred to a study health center for TB treatment after a diagnosis is established elsewhere (*i.e,* noted in the remarks in NTLP Laboratory Register);
4. Started on TB treatment for extra-pulmonary TB only (*i.e.,* recorded as Disease Class=EP (Extra Pulmonary TB) in NTLP Treatment Register).

The patient population contributing to the primary analysis of all outcomes will not include patients age <18 years and those with a documented prior history of TB treatment (e.g., reason for Xpert testing or TB treatment marked as treatment failure, relapse, treatment after loss to follow-up, etc.). In addition, patients identified as RIF resistant by Xpert testing will not contribute to primary and secondary analysis except for the number and proportion suspected or diagnosed with RIF-resistant TB.

Settings and Locations

20 microscopy centers met eligibility criteria based on an initial review of NTLP data. 5 (25%) health centers are level four health facilities (*i.e.* those serving counties with a target population of 100,000 people), 14 (70%) are level three (*i.e.* those serving sub-counties with a target population of 20,000 people), and one (5%) is a hospital. 18 (90%) health facilities are government-owned, one (St. Francis Njeru) is owned by an NGO, and one (Bishop Asili) is a private not for profit facility. The administrative

authority is the Ministry of Health for 18 (90%) health centers, the Uganda Catholic Medical Bureau for St. Francis Njeru, and the local government for Bukulula. 12 (60%) of these health centers are located in the Central region of Uganda, and eight (40%) in the Eastern region. Four health centers are located in the Mayuge district, three in Kayunga, three in Iganga, and one in each of the districts of Buikwe, Kalungu, Kamuli, Luwero, Lwengo, Mityana, Mpigi, Mubende, Nakasongola, and Wakiso.[6] District populations in 2014 ranged from 181,799 (Nakasongola) to 1,997,418 (Wakiso), with a median population of 465,099 (IQR 358,288-490,789).[7] Five (25%) health centers are in subcounties classified in the 2014 census as urban, while 15 (75%) are rural.[8] Health centers range in distance to central Kampala from 16 to 151 kilometers, with a median of 110 km (IQR 76-128). The range of distance from each health center to their primary Xpert hub is 1 to 50 km, median of 16 km (IQR 13-24).

3.3 Interventions

Intervention arm: Health centers randomized to the intervention arm will received the following intervention components: 1) onsite molecular testing for TB with GeneXpert as a replacement for sputum smear microscopy; 2) process redesign to facilitate same-day TB diagnosis and treatment; and 3) performance feedback of TB diagnostic evaluation quality indicators to health center staff.

Control arm: Health centers randomized to the control arm will follow the standard-of-care TB evaluation strategies, which are onsite ZN or LED fluorescence microscopy and hub-based GeneXpert testing for selected patients in accordance with Uganda NTLP guidelines.

3.4 Outcomes

Definitions used to assess study outcomes

- ***Number enrolled***: Number of eligible patients identified over a defined enrolment period through review of the NTLP Presumptive TB register, NTLP Laboratory register, GeneXpert laboratory requisition forms, GxAlert database and NTLP Treatment register at each study site (includes patients treated for TB without undergoing any sputum testing).

- ***Date enrolled***: Earliest date recorded in the NTLP Presumptive TB register, NTLP Laboratory register, GeneXpert laboratory requisition form, GxAlert database, or NTLP Treatment register. Records will be prospectively reviewed using name, sex, and age to identify patients presenting multiple times over the study period. Visits occurring within six months of the initial presentation will be considered as part of the same episode.

- ***Number referred for testing***: Number of eligible patients identified through review of the NTLP Presumptive TB register, NTLP Laboratory register, GeneXpert laboratory requisition forms, and GxAlert database at each study site.

- ***Number tested***: Number of eligible patients with any smear or Xpert result entered into the NTLP Presumptive TB register, NTLP Laboratory register, GeneXpert laboratory requisition forms, NTLP treatment register, or GxAlert database at each study site.

- **Number completing testing**: Number of eligible patients with one valid Xpert result plus number of eligible patients with a valid smear result entered into the NTLP Presumptive TB register, NTLP Laboratory register, GeneXpert laboratory requisition forms, GxAlert database, or NTLP treatment register. Definitions of valid results are as follows:
    - A positive Xpert result with a semi-quantitative result of high, medium, low, or very low for all eligible patients; a positive Xpert Ultra result with a semi-quantitative result of trace for eligible HIV-positive patients; or second positive Xpert Ultra result if the initial result is trace-positive for eligible HIV-negative patients;[9]
    - A negative Xpert result for all eligible patients;
    - One positive or two negative smear results for eligible HIV-negative/HIV status unknown patients.

- ***Number diagnosed***: Number of eligible patients with microbiologically-confirmed TB via a positive smear and/or valid Xpert test result entered into the NTLP Presumptive TB register, NTLP Laboratory register, GeneXpert laboratory requisition forms, NTLP treatment register, and/or GxAlert database within 6 months of *date enrolled*.

- ***Number with suspected RIF resistant TB***: Number of eligible patients with RIF resistance identified by Xpert testing entered into the GxAlert database, GeneXpert laboratory requisition forms, and/or NTLP Laboratory registers within 6 months of *date enrolled*.

- ***Number with confirmed RIF resistant TB***: Number of eligible patients with RIF resistance identified by Xpert testing entered into the GxAlert database, GeneXpert laboratory requisition forms, and/or NTLP Laboratory registers, and RIF resistance confirmed by culture-based DST or a second molecular assay as determined by review of Lab Register at MDR treatment center within 6 months of *date enrolled*.

- ***Number treated***: Number of eligible patients entered into the NTLP Treatment register as having started Category I or II regimen, or started on MDR treatment at the MDR treatment center as determined by review of Treatment Register at MDR treatment center within 6 months of *date enrolled.*

- ***Number completing treatment***: *Number treated* and with a treatment outcome of cured or completed entered into the NTLP Treatment register or indicated by MDR treatment center staff through direct follow up.

- ***Number died***: *Number treated* and with a treatment outcome of died entered into the NTLP Treatment register within 6 months of *date enrolled*, or number eligible with treatment outcome entered as died on direct follow-up form.

- ***Time-to-diagnosis***: Number of days from *date enrolled* to earliest date of positive smear or valid Xpert result recorded in NTLP Presumptive TB register, NTLP Laboratory register, GeneXpert laboratory requisition form, NTLP treatment register, or GxAlert database within 6 months of *date enrolled*.

- ***Time-to-treatment***: Number of days from *date enrolled* to treatment start date entered into NTLP Treatment register if treatment start date is within 6 months of *date enrolled.*

Primary Outcome

Outcome	Numerator	Denominator
Number treated for microbiologically-confirmed TB within 14 days of referral for sputum-based testing	Number diagnosed<br>and time-to-treatment within 14 days	None

Secondary Outcomes

**A. Testing**		
**Outcome**	**Numerator**	**Denominator**
Number referred for TB testing	Number referred for testing	None
Proportion completing testing	Number completing testing	Number referred for testing
**B. Diagnosis**		
**Outcome**	**Numerator**	**Denominator**
Number diagnosed with microbiologically-confirmed TB*	Number diagnosed	None
Proportion diagnosed with microbiologically-confirmed TB*	Number diagnosed	Number referred for testing

Number suspected/diagnosed with RIF-resistant TB*	Number with suspected/confirmed RIF resistant TB	None
Proportion suspected/diagnosed with RIF-resistant TB*	Number with suspected/confirmed RIF resistant TB	Number referred for testing
Time to microbiologically-confirmed TB**	Time-to-diagnosis if microbiologically-confirmed TB	None
**C. Treatment**		
**Outcome**	**Numerator**	**Denominator**
Number treated for TB*	Number treated	None
Proportion treated for TB*	Number treated	Number enrolled
Number treated for microbiologically-confirmed TB*	Number diagnosed AND treated	None
Proportion treated for microbiologically-confirmed TB*	Number diagnosed AND treated	Number referred for testing
Proportion with microbiologically-confirmed TB treated*	Number diagnosed AND treated	Number diagnosed
Time-to-treatment of microbiologically-confirmed TB**	Time-to-treatment if microbiologically-confirmed TB and treated	None
**D. Follow-up**		
**Outcome**	**Numerator**	**Denominator**
Number with microbiologically-confirmed TB completing treatment	Number diagnosed AND completing treatment	None
Proportion with microbiologically-confirmed TB completing treatment	Number diagnosed AND completing treatment	Number diagnosed AND treated
Number who died within 6 months	Number died	None
Proportion who died within 6 months***	Number died	Number enrolled

*Outcome will be assessed within 1 day and within 14 days of initial sputum submission. One day was chosen because the intervention focuses on same-day diagnosis and treatment. 14 days was chosen because the diagnostic process could take 7-10 days in the control arm depending on the frequency of sample transport to Xpert testing sites.
**For the control arm, missing Xpert result dates will be recorded as the next scheduled date of sputum transportation from the health center to the GeneXpert facility (results from the previous batch of testing are returned when the next batch of samples are picked up). For the intervention arm, missing Xpert result dates will be recorded as the day following sample collection.
***Treatment outcomes will be assessed at 7, 9, 12, and 24-month post-treatment initiation intervals determined by drug regimen.

3.5 Sample Size

Sample size

Based on pre-randomization data collected from January to December 2017, we estimate 5360 patients will be enrolled across 20 study sites over the 18-month enrolment period. Other study intervals ranging from 12 to 24 months are considered.

Justification

Original detectable effect size estimates: The study is based on the health center being the unit of randomization and aims to demonstrate the superiority of the intervention arm. The sample size calculation uses formulae appropriate for cluster-randomized trials with a parallel design and stratified and/or restricted randomization, including the addition of one extra cluster per arm to allow for the loss of degrees of freedom

due to stratification to obtain conservative estimates.[4] The original detectable effect size estimates were based on the outcome being the proportion of patients treated for microbiologically-confirmed TB within two weeks of referral for sputum-based testing. A type I error of 5% and power of 90% was assumed. Pre-randomization data collected from January to December 2017 from the 20 selected trial sites in Uganda suggests the average proportion of patients referred for TB evaluation who initiate treatment for active TB within two weeks is 6.7%, and the coefficient of variation (*k*) between clusters is 0.36, with a harmonic mean of 268 patients enrolled at each health center cluster during the 18-month enrolment period. Based on these assumptions and assuming a type I error of 5%, we will have 90% power to detect a 6% or greater absolute increase in the outcome proportion in the intervention arm (see **Table 1**). Table 1 also shows detectable absolute effect sizes with varying power (80%, 85%, 90%) during various trial time intervals and respective average cluster size estimates.

The minimum detectable effect size is based on published literature, our preliminary data demonstrating the increased sensitivity of Xpert Ultra vs. smear microscopy (95% vs. 40%), and reduced pre-treatment loss to follow-up expected in the intervention vs. control arms due to rapid, onsite molecular testing (7% vs. 30%). When applying these parameters to a population of 1000 individuals with TB prevalence of 10%, we would expect 95 patients to be diagnosed with TB and 88 to start treatment within 14 days in the intervention arm, and 40 patients to be diagnosed with TB and 28 to start treatment within 14 days in the control arm. Thus, the expected difference in the primary outcome proportion would be 8.8% vs. 2.8%, or 6%.[5,6]

Table 1. Absolute difference in the proportion of clinic attendees initiating TB treatment within 2 weeks of initial sputum submission, among those referred for TB testing

	Effect size (10 clusters per arm, *k*=0.36)		
Average cluster size	80% Power	85% Power	90% Power
178 (*12 months*)	5.9%	6.5%	7.4%
223 (*15 months*)	5.7%	6.3%	7.1%
268 (*18 months*)	5.5%	6.1%	6.9%
312 (*21 months*)	5.4%	6.0%	6.7%
357 (*24 months*)	5.3%	5.9%	6.6%

Figure 1. Effect size by cluster size. Number of clusters per arm is 10, *k*=0.36.

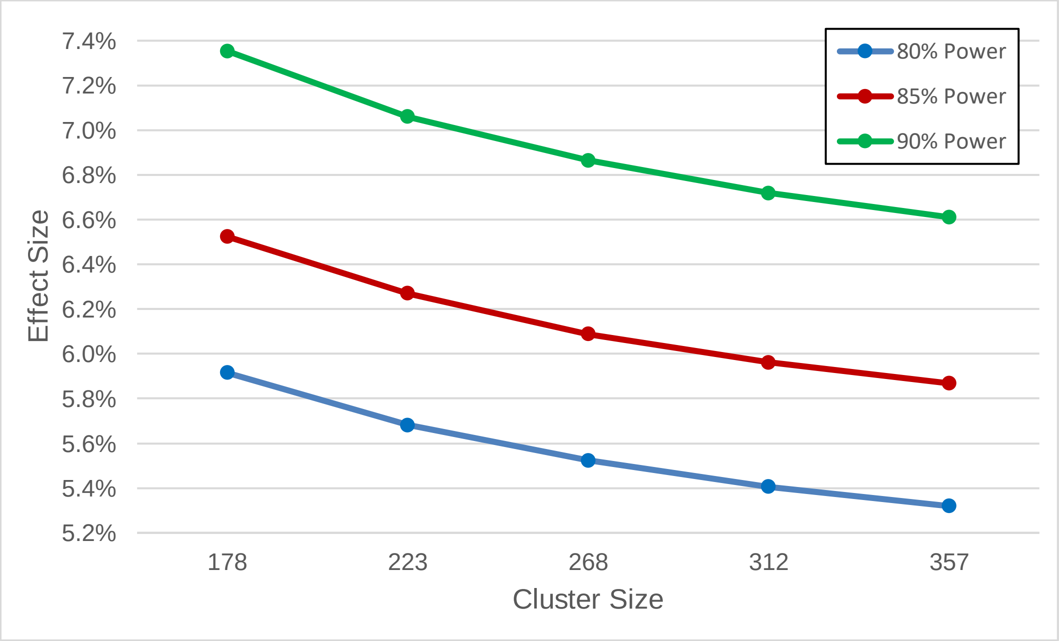

Revised detectable effect size estimates: The revised detectable effect size estimates are for the outcome being the number of patients treated for microbiologically-confirmed TB within two weeks of referral for

sputum-based testing. We first used 10 months of pre-intervention period data across all 20 clinics to assess 1) the geometric mean number of patients diagnosed and treated for TB within 14 days (new primary outcome), the natural log (ln) of the geometric mean and 3) the standard deviation of the natural log at control sites, intervention sites and overall.. For the pre-intervention period, the intervention clinics have a lower geometric mean number of clinic attendees who were diagnosed with TB and started on TB treatment within 14 days compared with the control arm:

	Geometric mean	Mean of ln outcome	SD of ln outcome
Control	14.70	2.69	0.23
Intervention	6.49	1.87	0.32

We then estimated the detectable effect size (expressed as a geometric mean ratio). The calculations assume the same parameters as for the original primary outcome (10 clinics/arm, 18-month trial duration to achieve geometric mean of 286 patients/cluster in control and intervention arms). Table 1 below shows the detectable effect size assuming within-arm SD of ln outcome of 0.2 to 0.3 and power of 80-90%. The detectable effect sizes shown below are conservative (calculations do not take into account the baseline differences between arms in the new primary outcome and are based on 10-months rather than full 24-months of pre-intervention data). Final calculations will be repeated once baseline data is fully entered and available for analysis.

Table 3: Detectable effect sizes

SD of ln outcome	Geometric Mean Ratio of the new primary outcome (intervention vs control)	
	80% power	90% power
0.2	1.30	1.36
0.3	1.49	1.58

We believe a GMR of 1.30-1.58 is a reasonable detectable effect size. Xpert MTB/RIF is twice as sensitive as smear microscopy (double the number of confirmed TB cases) and we expect onsite testing to reduce pre-treatment loss to follow-up by at least half (from 30% to <15%).

3.6 Blinding

The trial will be open-label for participants and researchers, as blinding of the assigned intervention is not feasible given intervention implementation at the health center level. Staff, however, will be blinded to the intervention allocations through the use of codes to identify clusters rather than health center names. Where possible, the investigators and study staff will be masked to ongoing aggregated data by study. The trial statistician and data manager will have access to the aggregate data. Some site-level data will be used for performance feedback to sites in the intervention arm; this data will not be a comparison to baseline sites across study arms.

4. Randomization

4.1 Sequence Generation

A random allocation sequence will be generated by STATA 14 (StataCorp LP, College Station, TX). Randomisation of clusters to the intervention or control arm was conducted using stratification and restriction. Stratified randomisation was used to help reduce between-cluster variability and achieve balance at baseline. Using data from 2017 on the percentage of adult clinic attendees being investigated for TB who started TB treatment within 14 days, the clusters were stratified into two equal-sized groups – “low” and “high” percentages of this outcome based on the median. Restricted randomisation was used to help ensure balance for key factors. Variables we restricted on were; year cluster enrolled in project (2017 vs. 2018); sputum transport frequency to Xpert hub; distance to Xpert hub; number of patients evaluated for

TB; percentage starting TB treatment within 14d; urban vs. rural health clinics; HIV prevalence; number of facilities per district. For these restriction criteria the overall proportion of unacceptable allocations was calculated and the the validity assessed using the validity matrix.[1] From a total of 63,504 possible randomisations based on two equal-sized strata, applying the restriction criteria resulted in 11,392 randomisations; one of these was chosen at random.

4.2 Allocation concealment mechanism

Allocation concealment will be used to prevent selection bias by concealing from the research staff the allocation sequence until assignment has occurred.

4.3 Implementation

The trial statistician will generate the random allocation assignments, and clusters will be enrolled by local study staff. Eligible individual patients will be included in clusters through complete enumeration. District health officers provide administrative authority and consent to health center participation prior to randomization. Consent is documented in a signed Memorandum of Understanding (MOU) between the district and the National Tuberculosis and Leprosy Control Program (NTLP), endorsed by the Chief Administrative Officer, the District Health Officer, and the Program Manager for NTLP. A copy of the agreement is provided to the facility in-charge at each health center.

Public ceremony for randomization will be held in Kampala, Uganda. Facility in-charges, district health officers, and representatives from NTLP will attend.

4.4 Statistical Methods

4.4.1 General analyses principles

We will calculate and compare all effectiveness outcomes for the two trial arms using prespecified analyses following the intention-to-treat principle. Statistical analysis methods to determine intervention effect on outcomes will be used that are appropriate for CRTs with a small number of clusters. Analysis of cluster-level summaries is based on a total of 20 clusters (10 per arm), each comprised of one health center, and each being given equal weight. Effect estimates will be reported with 95% confidence intervals, and an associated P-value.

4.4.2 Baseline data

Baseline variables will be cross-tabulated to check for appropriate balance between study arms and to characterize the study population using summary statistics both at cluster and individual levels (**Table 1, 2**).

Cluster (*i.e.* health center) level data will be collapsed across study arms and compared using mean, median and range with standard deviations and/or interquartile ranges as appropriate. The basis of this analysis will be data on demographics, health center and laboratory infrastructure, organizational hierarchy, Xpert referral network, and TB testing and treatment collected during site assessment, recruitment, and guidelines training.

Individual level data for all patients eligible for analysis will also be collapsed across study arms and compared using mean, median and range with standard deviations and/or interquartile ranges as appropriate. These individual factors include age, sex, HIV status, and ART status.

We will also summarize data obtained during the pre-randomization period for the cohort of health centers and patients who are being enrolled in the study and randomized to a trial arm.

Descriptive data between control and intervention arms will be presented in tabular format in lieu of formal statistical comparison or significance tests, and will be reviewed to confirm comparability and identify any substantial imbalances. Adjusted effect estimates will be calculated by study arm to account for imbalance

of covariates which are risk factors for the outcome of interest. Losses in degrees of freedom due to cluster-level covariate adjustments will be accounted for in all calculations.

4.4.3 Primary analysis of primary outcome

Analysis will be conducted at the cluster (clinic)-level. The outcome is the count of the number treated for microbiologically-confirmed TB within two weeks of referral for sputum-based testing at the clinic-level. Initially Poisson regression will be used, with an offset at the clinic level of the total number of months the clinic contributes individuals to the study (starting from 22 October 2018). Evidence for over-dispersion will be assessed and if over-dispersion is detected then negative binomial regression will be used, using the same offset. Either model will adjust for randomisation strata and the count of microbiologically-confirmed TB cases that initiated on treatment within 14 days in the 12 month period before 22 October 2018. The exact functional form of this covariate will be identified through fractional polynomials or by using $\log_e$ transformation. Further adjustment for clinic-level factors that show imbalance will be explored, though the number of adjustment factors will be limited as all are at the clinic-level. See tables 4 and 5a.

Xpert Ultra cartridges will be used in our intervention (see Outcomes Definitions Section 3.4). Two sensitivity analyses will be performed reclassifying 1) all trace-positive results as negative and 2) trace-positive results in HIV negative patients as true positive (even if not confirmed by a second positive test) to determine the impact of Ultra on the primary outcome.

No sub-group analyses for strata defined at the individual-level (eg., sex and HIV status) will be conducted as the primary outcome analyses is conducted at the cluster-level.

4.4.4 Analysis of secondary outcomes

Secondary outcomes are reported as either counts, proportions, or time to event, and are derived from data collected across routine data sources.

All analyses will adjust for randomisation strata. Intervention effect estimates will be reported for analyses (1) adjusting for randomisation strata only and (2) adjusting for randomisation strata and individual-level (proportions and time to event only) and clinic-level factors considered imbalanced, as outlined below. See table 5b.

4.4.4.1 Count outcomes:

The analytic approach will be the same as described for the primary outcome. . The outcome is the count of the respective outcome at the clinic-level. Analysis will be conducted at the cluster (clinic)-level. Either Poisson or negative binomial regression will be used, depending on over-dispersion. Adjustment will be made for the clinic-level count of the outcome using data in the 12 month period before 22 October 2018. The exact functional form of this covariate will be identified through fractional polynomials or by using $\log_e$ transformation.

4.4.4.2 *Proportions*

The analysis will give each cluster equal weight. The overall risk for each cluster will be calculated and shown, by strata and arm. A log transformation (where necessary) will be applied to the risk for each cluster. If a cluster has no events, 0.5 event will be added to all clusters in order that the log transformation can be conducted. The mean and standard deviation of these log risks will be used to obtain the geometric mean (GM) and associated 95% CI for each arm of the study.
The risk ratio is estimated using linear regression of the log risks/rates/means on stratum and arm.
An approximate standard error for the difference in log (GM) between arms is obtained based on the residual mean square from a two-way analysis of variance (ANOVA) of the cluster log risk on stratum, study arm and the interaction between stratum and study arm. The 95% CI is calculated from this standard error, using a t-statistic with 16 degrees of freedom.

Logistic regression will be used to adjust for any baseline imbalance at the individual level, adopting a two-stage approach. The regression model will include terms for the adjustment factors (individual level) and strata, but not study arm. For each cluster the fitted model will be used to obtain the ratio of observed to expected (O/E) events, and a log transformation will be applied to this ratio, where appropriate.
Linear regression of the log (O/E) on stratum and arm (2-way ANOVA) will be used to estimate the risk ratio. The variance for the ratio of mean O/E is calculated from the residual mean square from an ANOVA of log(O/E) on stratum, arm and the interaction between stratum and arm. The 95% CI is calculated from this variance, using a t-statistic with 16 degrees of freedom. Limited adjustment for individual level factors is only possible; age, sex, HIV status, and ART status. Adjustment will only be done if data completeness for the variable is more than 80% in both trial arms.

If imbalance is observed for the equivalent cluster-level outcome in the 12 month period before 22 October 2018, adjustment at the second stage described above will be conducted, with appropriate adjustment for the degrees of freedom for the intervention effect. The exact functional form of this cluster-level covariate will be identified through fractional polynomials or by using $\log_e$ transformation.

4.4.4.1 *Time to event*

The analysis will give each cluster equal weight. The arithmetic and geometric mean time to event (amongst all those experiencing the effect) will be calculated for each cluster and shown, by strata and arm. A log transformation of the individual-level time to event will likely be necessary as the times to event may be positively skewed, resulting in geometric means. Clusters where no individuals contribute time to event will be excluded from the analysis.

ANOVA will be used to calculate either the difference in means between arms or geometric mean ratio, depending on whether a log transformation was used. A two-stage approach, similar to as described above, will be used to adjust for confounders at the individual level. Limited adjustment for individual level factors is only possible; age, sex, HIV status, and ART status. Adjustment will only be done if data completeness for the variable is more than 80% in both trial arms. If imbalance is observed for the equivalent cluster-level outcome in the 12 month period before 22 October 2018, adjustment at the second stage described above will be conducted, with appropriate adjustment for the degrees of freedom for the intervention effect. The exact functional form of this cluster-level covariate will be identified through fractional polynomials or by using $\log_e$ transformation.

4.4.5 Sub-group analyses for secondary outcomes

Pre-specified sub-group analyses for a subset of secondary outcomes will be conducted to assess whether the effect of the intervention differs between sub-groups. The sub-groups are (1) HIV status at enrolment (self-reported, laboratory test resulted as noted on primary data sources, or through pre-ART and ART register data extraction), (2) and sex. The effect of the intervention will be estimated for each sub-group, and P-values for effect modification, appropriate for individual-level covariates, will be reported.[2] These sub-group analyses will be conducted for the following secondary outcomes: proportion completing testing; proportion diagnosed with microbiologically-confirmed TB; proportion treated for microbiologically-confirmed TB within two weeks of referral for sputum-based testing; and the proportion who died within 6 months. See table 5c.

Tables

Table 1. Summary of baseline study variables

		Intervention	Control
Year of enrolment into project	*2017 or 2018*		
Distance to Xpert hub	*Median (IQR)*		
Sputum transport frequency to Xpert hub	*Once per week or ≥2 times per week*		
Total number referred for TB testing*	#		
Average number referred for TB testing, across clusters*	Harmonic mean		
Region	*Central or Eastern, % (n/N)*		
District	*% per district*		
Geographical area	*Urban or rural, % (n/N)*		
Population of sub-county	#		
Patients undergoing TB testing who are diagnosed with and treated for TB within 14 days*	*(< 6.2% or ≥ 6.2%)*		
HIV prevalence among TB patients*	*% (n/N)*		

* in the 12 month period before 22 October 2018

Table 2. Summary of demographic variables among those referred for TB testing* (collapsed over cluster)

		Intervention	Control
Total number referred for TB testing	#		
Average number referred for TB testing, across clusters	Harmonic mean		
Missing data for sex	*% (n/N_{total})*		
Female	*% ($n/N_{non\text{-}miss}$)*		
Missing data for age	*% (n/ N_{total})*		
Age	*Median (IQR), $N_{non\text{-}miss}$*		
HIV status known:	*% (n/N_{total})*		
HIV-positive	*% ($n/N_{non\text{-}miss}$)*		
ART status known:	*% ($n/N_{total\ HIV+}$)*		
ART prevalence	*% ($n/N_{non\text{-}miss\ HIV+}$)*		

* in the intervention period after 22 October 2018

Table 3: Summary of numbers referred for TB testing* in intervention and control arms (collapsed over cluster) using data from record review and facility follow up.

		Intervention	Control
Xpert result	*Negative*<br>*Positive*<br>*Unknown*		
Semi-Quantitative result	*High*<br>*Medium*<br>*Low*<br>*Very Low*<br>*Trace*		
RIF result	*Negative*<br>*Positive*<br>*Indeterminate/Invalid/No Result/Error*<br>*Unknown/No Result*		
MDR status	*Yes, confirmed positive by DST*<br>*No, confirmed negative by DST*<br>*Unknown*		
Smear result	*Negative*<br>*Positive*<br>*Unknown*		
Treatment status	*Yes, on treatment*<br>*No, not on treatment*<br>*Unknown*		
Treatment category	*Category 1- 2RHZE/6EH*<br>*Category 1- 2RHZE/4RH*<br>*Category 2-2SRHZE/1RHZE/5RHE*<br>*Category 3-2RHE/4RH*<br>*Other*		
Treatment outcome	*Cured*<br>*Completed*<br>*Failure (smear positive)*<br>*Died*<br>*Transferred out*<br>*Lost to Follow up*		

* in the period after 22 October 2018

Table 4: Primary outcome, number treated for microbiologically-confirmed TB within two weeks of initial sputum submission, summary of number diagnosed within 14 days, number referred for TB testing, and proportion, by control and intervention arm

	Control arm		Intervention arm	
Cluster	# diagnosed and treated within 14 days	total number of months the clinic contributes individuals to the study	# diagnosed and treated within 14 days	total number of months the clinic contributes individuals to the study
1				
2				
3				
4				
5				
6				
7				
8				
9				
10				
Geometric Mean				

Table 5a: Primary outcome: number treated within 14 days, unadjusted and adjusted risk ratio (95% confidence intervals) and associated P-values

Primary outcome	I: #	C: #	Unadjusted RR* (95% CI)	P-value	Adjusted RR** (95% CI)	P-value
Primary analysis						
Sensitivity analysis 1						
Sensitivity analysis 2						

I Intervention ; C control

* adjusted for randomisation strata; ** adjusted for randomisation strata and count of microbiologically-confirmed TB cases that initiated on treatment within 14 days in the 12 month period before 22 October 2018

Table 5b: Secondary outcomes: unadjusted and adjusted risk ratio (95% confidence intervals) and associated P-values

Secondary outcomes		I:	C:	RR (95% CI)	P-value	Adjusted RR (95% CI)	P-value
A. Testing							
Number referred for TB testing	GM						
Proportion completing testing	% (n/N)						
B. Diagnosis							
Number diagnosed with microbiologically-confirmed TB (by day 1)	GM						
Number diagnosed with microbiologically-confirmed TB (by day 14)	GM						
Proportion diagnosed with microbiologically-confirmed TB (by day 1)	% (n/N)						
Proportion diagnosed with microbiologically-confirmed TB (by day 14)	% (n/N)						
Number suspected/diagnosed with RIF-resistant TB (by day 1)	GM						
Number suspected/diagnosed with RIF-resistant TB (by day 14)	GM						

Proportion suspected/diagnosed with RIF-resistant TB (by day 1)	% (n/N)						
Proportion suspected/diagnosed with RIF-resistant TB (by day 14)	% (n/N)						
Time to microbiologically-confirmed TB	GM						
C. Treatment							
Number treated for TB (by day 1)	GM						
Number treated for TB (by day 14)	GM						
Proportion treated for TB (by day 1)	% (n/N)						
Proportion treated for TB (by day 14)	% (n/N)						
Number treated for microbiologically-confirmed TB (by day 1)	GM						
Number treated for microbiologically-confirmed TB (by day 14)	GM						
Proportion treated for microbiologically-confirmed TB (by day 1)	% (n/N)						
Proportion treated for microbiologically-confirmed TB (by day 14)	% (n/N)						
Proportion with microbiologically-confirmed TB treated (by day 1)	% (n/N)						
Proportion with microbiologically-confirmed TB treated (by day 1)	% (n/N)						
Time-to-treatment of microbiologically-confirmed TB	GM						
D. Follow-up							
Number with microbiologically-confirmed TB completing treatment	GM						
Proportion with microbiologically-confirmed TB completing treatment	% (n/N)						
Number who died within 6 months	GM						
Proportion who died within 6 months	% (n/N)						

GM geometric mean

Table 5c: Sub-group analyses for selected secondary outcomes: stratum-specific unadjusted and adjusted risk ratios (95% confidence intervals) and associated P-values for interaction

Secondary outcomes		I:	C:	RR (95% CI)	P-value	Adjusted RR (95% CI)	P-value	P-value for interaction
A. Proportion completing testing								
HIV-positive	% (n/N)							
HIV-negative	% (n/N)							
Male	% (n/N)							
Female	% (n/N)							
B. Proportion diagnosed with microbiologically-confirmed TB (by day 14)								
HIV-positive	% (n/N)							
HIV-negative	% (n/N)							
Male	% (n/N)							
Female	% (n/N)							
C. Proportion treated for microbiologically-confirmed TB (by day 14)								
HIV-positive	% (n/N)							
HIV-negative	% (n/N)							
Male	% (n/N)							
Female	% (n/N)							
D. Proportion who died within 6 months								
HIV-positive	% (n/N)							
HIV-negative	% (n/N)							
Male	% (n/N)							
Female	% (n/N)							

References

1. Xpert Omni Performance Evaluation (XPEL TB). 2017; https://clinicaltrials.gov/ct2/show/NCT03044158.
2. Xpert Omni Performance Evaluation for Linkage to Tuberculosis Care: The XPEL TB Trial. 2016; http://www.pactr.org/ATMWeb/appmanager/atm/atmregistry?dar=true&tNo=PACTR201610001763265.
3. Campbell MK, Piaggio G, Elbourne DR, Altman DG, Group C. Consort 2010 statement: extension to cluster randomised trials. BMJ. 2012;345:e5661.
4. Hayes RJ, Moulton LH. Cluster Randomized Trials. Boca Raton, Florida, USA: CRC Press; 2009.
5. Farr K, Nalugwa T, Shete P, et al. Late Breaking Abstract - The tuberculosis diagnostic evaluation cascade of care at microscopy centers linked to Xpert MTB/RIF hubs in Uganda. European Respiratory Journal Sep 2017, 50 (suppl 61) OA1943; DOI: 10.1183/1393003.congress-2017.OA1943.
6. Ministry of Health. Health Facilities Inventory. Kampala, Uganda 2012.
7. Uganda Bureau of Statistics. Census 2014 Results. 2014; http://www.ubos.org/2014-census/census-2014-final-results/.
8. Uganda National Population and Housing Census 2014 Provisional Results. 2014; http://ugandadata.org/redbin/RpWebEngine.exe/Portal?&BASE=cen2014.
9. World Health Organization. WHO Meeting Report of a Technical Expert Consultation: Non-inferiority analysis of Xpert MTB/RIF Ultra compared to Xpert MTB/RIF 2017; http://apps.who.int/iris/bitstream/10665/254792/1/WHO-HTM-TB-2017.04-eng.pdf.
10. Moulton LH. Covariate-based constrained randomization of group-randomized trials. Clinical trials. 2004;1(3):297-305.
11. Sismanidis C, Moulton LH, Ayles H, et al. Restricted randomization of ZAMSTAR: a 2 x 2 factorial cluster randomized trial. Clinical trials. 2008;5(4):316-327.
12. Cheung YB, Jeffries D, Thomson A, Milligan P. A simple approach to test for interaction between intervention and an individual-level variable in community randomized trials. Trop Med Int Health. 2008;13(2):247-255.
13. Analysis of TB prevalence and drug resistance surveys. TB surveillance and surveys: a training workshop for consultants. 2011; http://www.who.int/tb/advisory_bodies/impact_measurement_taskforce/meetings/survmay11_analysis_clustering_missing_data_sismanidis.pdf

Figure X. Comparison of TB diagnostic evaluation in the intervention and control arms

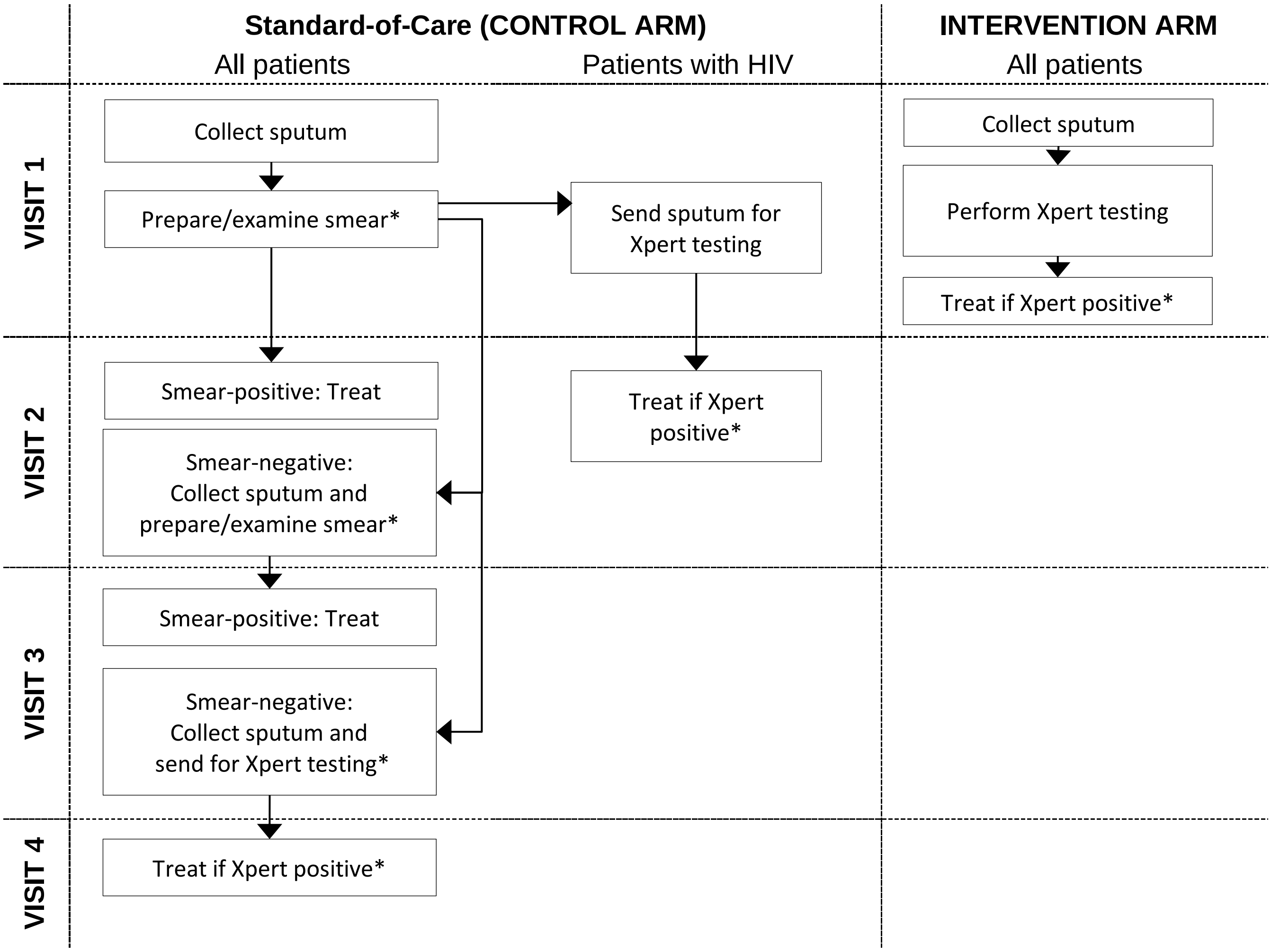

*Consider empiric treatment or additional testing if test results are negative or invalid

CONSORT diagram

CONSORT diagram will be constructed show individual and cluster-level data at baseline and follow-up and based on the CONSORT extension to CRTs.

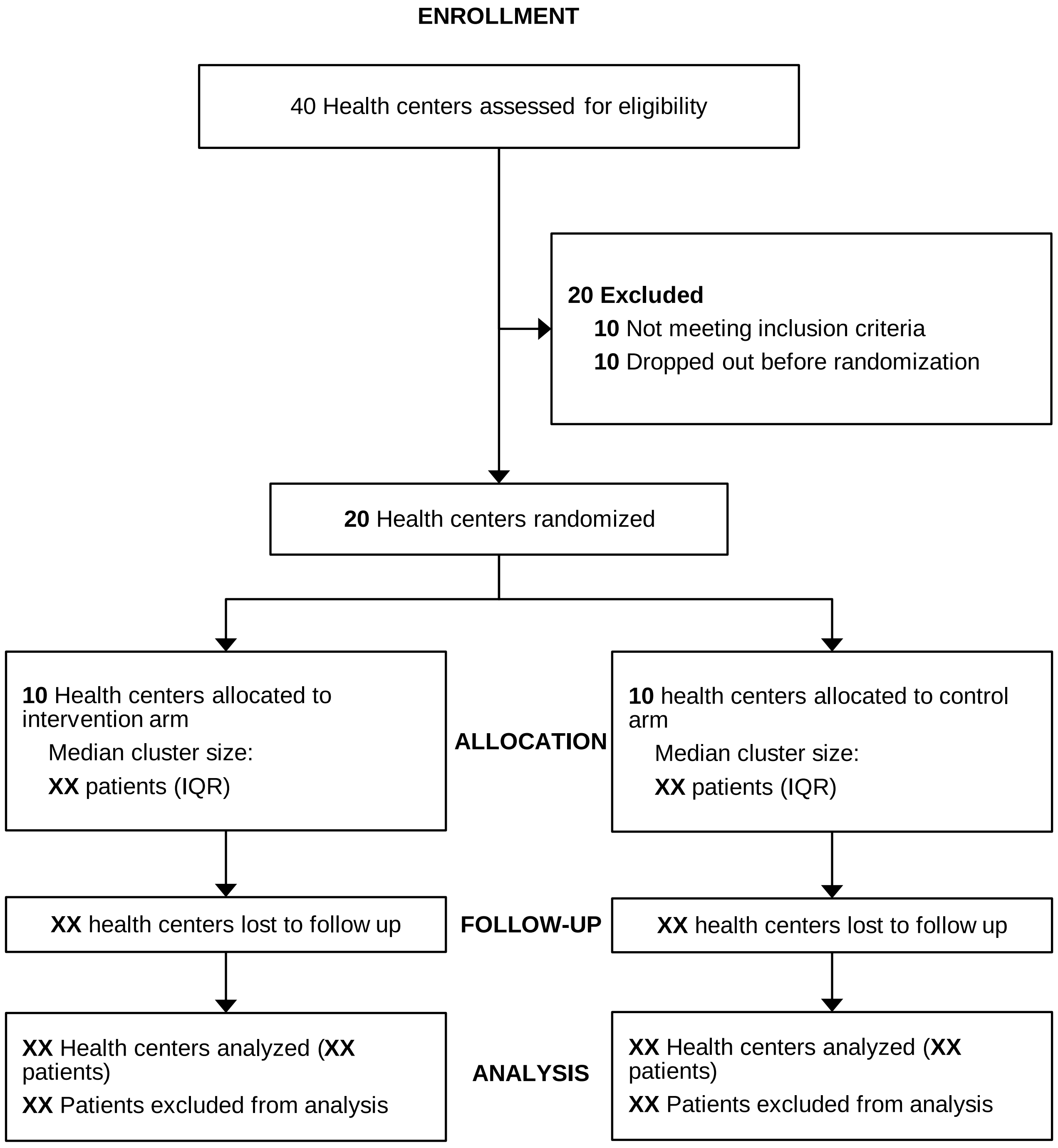

1. Moulton LH. Covariate-based constrained randomization of group-randomized trials. *Clinical trials.* 2004;1(3):297-305.
2. Cheung YB, Jeffries D, Thomson A, Milligan P. A simple approach to test for interaction between intervention and an individual-level variable in community randomized trials. *Trop Med Int Health.* 2008;13(2):247-255.

Statistical Analysis Plan: XPEL TB

**Full Title**	GeneXpert Performance Evaluation for Linkage to Tuberculosis Care: The XPEL TB Trial		
**Acronym**	XPEL TB		
**Document History**	**Version No.**	**Version Date**	**Description of Change**
	1.0	2019-10-11	Initial release
	1.1	2019-11-21	Updated based on Investigators' inputs
	1.2	2020-01-10	Version circulated to TSC
	1.3	2020-03-04	Final version
	1.4	2020-8-06	Updated appendix to include outcome details omitted from final version
	1.5	2021-07-12	Updated the denominator for proportion outcomes to improve clarity for reporting purposes
**Trial Registration**	ClinicalTrials.gov (NCT03044158)<br>Pan African Clinical Trials Registry (PACTR201610001763265)		
**Principal Investigators**	Adithya Cattamanchi, Achilles Katamba		
**SAP Authors**	Katherine Fielding, Katherine Farr, Tania Reza		

1. Introduction

1.1 Aim

To assess the effectiveness, implementation and costs of a streamlined TB diagnostic evaluation strategy based around rapid, onsite molecular testing

2. Background and Objectives

2.1 Specific Objectives

1. To compare patient outcomes at health centers randomized to intervention vs. standard-of-care TB diagnostic evaluation strategies.
    a. Intervention: Onsite molecular testing for TB with GeneXpert device + process redesign to facilitate same-day TB diagnosis and treatment + performance feedback
    b. Standard-of-care: Onsite ZN or LED fluorescence microscopy + hub-based GeneXpert testing per existing protocols
2. To identify processes and contextual factors that influence the effectiveness and fidelity of the intervention TB diagnostic evaluation strategy.
3. To compare the costs and epidemiological impact of intervention vs. standard-of-care TB diagnostic evaluation strategies.

2.2 Summary of aims

The study proposes to conduct a pragmatic, parallel cluster-randomized trial (CRT) with nested mixed methods, health economic and modelling studies to evaluate the effectiveness, implementation and costs of the intervention strategy relative to standard TB diagnostic evaluation at community health centers that are part of Xpert referral networks in Uganda. The effectiveness of the intervention strategy will be assessed using routine data collected as part of mandatory reporting to the Uganda NTLP on consecutive patients who present to participating health centers during the 18-month enrolment period and meet eligibility criteria. Selected patients and providers who provide informed consent will also be surveyed and/or interviewed to identify reasons for its success or failure across study sites (Aim 2), and to collect relevant cost data for analyses of cost-effectiveness and epidemiologic impact (Aim 3).

2.3 General comments

Trial reporting will follow the Standards for Reporting Implementation Studies (STaRI) checklist to evaluate the trial's coprimary aims: clinical effectiveness and implementation of onsite Xpert testing.[1] In addition, reporting will include elements from the CONSORT 2010 extension checklist for cluster randomized trials, such as the randomization process and a participant flow diagram.[2]

This Statistical Analysis Plan summarizes analyses for all effectiveness outcomes, including the primary outcome of the number of patients treated for microbiologically-confirmed TB within 14 days of referral for sputum-based testing. Secondary outcomes for ***testing*** (number and proportion of patients completing TB testing), ***diagnosis*** (number and proportion diagnosed with microbiologically-confirmed TB, number and proportion diagnosed with RIF-resistant TB, time to microbiologically-confirmed TB), ***treatment*** (number and proportion treated for TB, time to treatment of microbiologically-confirmed TB), and ***follow-up*** (number and proportion with microbiologically-confirmed TB completing treatment, number and proportion who died within 6 months) will also be assessed. These endpoints will be evaluated through review of TB registers and GeneXpert machine data for all patients initiating TB evaluation at participating health centers.

This Statistical Analysis Plan is written in support of and is predominantly consistent with the full trial protocol; however, this analysis plan takes precedence.

3. Trial Summary

3.1 Trial design

The study is a parallel cluster-randomized trial (CRT) with two arms of equal number of clusters. The type of cluster used is *institutional health units*; in this case, health centers that provide TB microscopy services in Uganda in partnership with the National TB Program. Entire health centers will be the unit of randomization rather than individual participants to reduce contamination. The unit of analysis will be patients undergoing TB diagnostic evaluation during the 18-month enrolment period. The trial includes nested mixed methods and economic analyses to assess the implementation and cost-effectiveness of the intervention.

3.2 Study population

Eligibility Criteria

A. *Site-level Inclusion Criteria*
    a. Use standard (multi-day) sputum smear microscopy as the primary method of TB diagnosis
    b. Participate in NTP-sponsored external quality assurance (EQA) for sputum smear microscopy
    c. Send samples to a district or regional hospital/health center for Xpert testing
B. *Site-level Exclusion Criteria*
    a. Do not agree to be randomized to standard-of-care vs. intervention arms
    b. Perform sputum smear examination on <150 patients per year (based on 2015 data)
    c. Diagnose <15 smear-positive TB cases per year (based on 2015 data)
C. Patient-level Inclusion Criteria
    a. Initiating TB evaluation at a study health center
D. Patient-level Exclusion Criteria
    a. Have sputum collected for monitoring of response to anti-TB therapy (i.e., recorded as a follow-up patient in routine data source);
    b. Have sputum collected as part of active, community-based case finding (e.g., contact tracing, community outreach campaign) (i.e., noted in the remarks in the NTLP Laboratory Register);
    c. Referred to a study health center for TB treatment after a diagnosis is established elsewhere (i.e., noted in the remarks in the NTLP Laboratory Register)
    d. Started on TB treatment for extra-pulmonary TB only (i.e., recorded as a Disease Class=EP (Extra Pulmonary TB) in the NTLP Treatment Register)

Settings and Locations

Twenty microscopy centers met eligibility criteria based on an initial review of NTLP data. 5 (25%) health centers are level four health facilities (*i.e.* those serving counties with a target population of 100,000 people), 14 (70%) are level three (*i.e.* those serving sub-counties with a target population of 20,000 people), and one (5%) is a hospital. 18 (90%) health facilities are government-owned, one (St. Francis Njeru) is owned by an NGO, and one (Bishop Asili) is a private not for profit facility. The administrative authority is the Ministry of Health for 18 (90%) health centers, the Uganda Catholic Medical Bureau for St. Francis Njeru, and the local government for Bukulula. 12 (60%) of these health centers are located in the Central region of Uganda, and eight (40%) in the Eastern region. Four health centers are located in the Mayuge district, three in Kayunga, three in Iganga, and one in each of the districts of Buikwe, Kalungu, Kamuli, Luwero, Lwengo, Mityana, Mpigi, Mubende, Nakasongola, and Wakiso.[3] District populations in 2014 ranged from 181,799 (Nakasongola) to 1,997,418 (Wakiso), with a median population of 465,099 (IQR 358,288-490,789).[4] Five (25%) health centers are in subcounties classified in the 2014 census as urban, while 15 (75%) are rural.[5] Health centers range in distance to central Kampala from 16 to 151 kilometers, with a median of 110 km (IQR 76-128). The range of distance from each health center to their primary Xpert hub is 1 to 50 km, median of 16 km (IQR 13-24).

3.3 Interventions

Intervention arm: Health centers randomized to the intervention arm will received the following intervention components: 1) onsite molecular testing for TB with GeneXpert as a replacement for sputum smear microscopy; 2) process redesign to facilitate same-day TB diagnosis and treatment; and 3) performance feedback of TB diagnostic evaluation quality indicators to health center staff.

Control arm: Health centers randomized to the control arm will follow the standard-of-care TB evaluation strategies, which are onsite ZN or LED fluorescence microscopy and hub-based GeneXpert testing for selected patients in accordance with Uganda NTLP guidelines.

3.4 Outcomes

Definitions used to assess study outcomes

- ***Eligible patient***: clinic attendees satisfying our inclusion and exclusion criteria (as listed above) AND excluding patients aged <18 years or those with a documented prior history of TB treatment (e.g., reason for Xpert testing or TB treatment marked as treatment failure, relapse, treatment after loss to follow-up, etc.). In addition, patients identified as RIF resistant by Xpert testing will not contribute to the primary and secondary analysis except for the number and proportion with RIF resistance detected by Xpert or RIF resistance confirmed by culture.

- ***Number enrolled (i.e., number evaluated for TB)***: Number of eligible patients identified over a defined enrolment period through review of the NTLP Presumptive TB register, NTLP Laboratory register, GeneXpert laboratory requisition forms and NTLP Treatment register at each study site (includes patients treated for TB without undergoing any sputum testing).

- ***Date enrolled***: Earliest date recorded in the NTLP Presumptive TB register, NTLP Laboratory register, GeneXpert laboratory requisition form, NTLP Treatment register or machine data. Records will be prospectively reviewed using name, sex, and age to identify patients presenting multiple times over the study period. Visits occurring within six months of enrollment will be considered as part of the same episode.

- ***Number referred for testing***: Number of eligible patients identified through review of the NTLP Presumptive TB register, NTLP Laboratory register and GeneXpert laboratory requisition forms at each study site. Patients indicated as new in the NTLP Treatment Register will also be considered testing referrals.

- ***Number tested***: Number of eligible patients with any smear or Xpert result entered into the NTLP Presumptive TB register, NTLP Laboratory register, GeneXpert laboratory requisition forms, or NTLP treatment register at each study site, or with a test result recorded in the onsite GeneXpert machine. Results must be dated within six months of enrolment. If result date is missing and cannot be found in the GeneXpert machine data, it will be assumed that the patient was tested within six months of enrolment.

- **Number completing testing**: Number of eligible patients with a valid Xpert result plus number of eligible patients with a valid smear result entered into the NTLP Presumptive TB register, NTLP Laboratory register, GeneXpert laboratory requisition forms or NTLP treatment register, or with a test result recorded in the GeneXpert machine data. Testing must be completed within six months of enrolment. If the test result is missing and cannot be found in the GeneXpert machine data, it will be assumed that the patient was tested within six months of enrolment. Definitions of valid results are as follows:
    - A positive Xpert (Ultra) result with a semi-quantitative result of high, medium, low, or very low for all eligible patients; a positive Xpert Ultra result with a semi-quantitative result of trace for eligible HIV-positive patients; or a second positive Xpert Ultra result (including trace) if the initial result is trace-positive for eligible HIV-negative patients;[6]

  - A negative Xpert result for all eligible patients;
  - One positive OR two negative smear results for eligible HIV-negative/HIV status unknown patients.

- ***Microbiologically-confirmed TB***: A positive smear and/or positive Xpert test result (as defined above) entered into the NTLP Presumptive TB register, NTLP Laboratory register, GeneXpert laboratory requisition forms, NTLP treatment register, and/or GeneXpert machine data (intervention sites only) within six months of enrolment. ***Number diagnosed***: Number of eligible patients with microbiologically-confirmed TB via a positive smear and/or valid Xpert test result entered into the NTLP Presumptive TB register, NTLP Laboratory register, GeneXpert laboratory requisition form, NTLP treatment register, and/or GeneXpert machine data within six months of enrolment.

- ***Number with RIF resistance detected by Xpert***: Number of eligible patients with RIF resistance identified by Xpert testing entered into the GeneXpert laboratory requisition forms and/or NTLP Laboratory registers within six months of enrolment .

- ***Number with RIF resistance confirmed by culture:*** Number of eligible patients with: (1) RIF resistance detected by Xpert testing entered into the GeneXpert laboratory requisition forms and/or NTLP Laboratory registers, and (2) RIF resistance confirmed by culture-based DST or a second molecular assay as determined by review of Lab Register at MDR treatment center within six months of enrolment .

- ***Number treated***: Number of eligible patients entered into the NTLP Treatment register as having started Category I or II regimen.

- ***Number completing treatment***: Number treated and with a treatment outcome of cured or completed entered into the NTLP Treatment register.

- ***Number died, among those initiated on TB treatment***: Number treated and with a treatment outcome of died entered into the NTLP Treatment register within six months of enrolment , or number treated and confirmed dead by next of kin with a date of death within six months of enrolment.

- ***Number died, among those with confirmed TB***: Number diagnosed and with a treatment outcome of died entered into the NTLP Treatment Register within six months of enrolment, or number diagnosed and confirmed dead by next of kin with a date of death within six months of enrolment.

- ***Number died, among all those eligible:*** Number treated and with a treatment outcome of died entered into the NTLP Treatment register within six months of enrolment, or number traced with a death recorded

- ***Time-to-diagnosis***: Number of days from *date enrolled* to earliest date of positive smear or valid Xpert result recorded in NTLP Presumptive TB register, NTLP Laboratory register, GeneXpert laboratory requisition form, or NTLP treatment register within six months of enrolment . For the control arm, attempts will be made to abstract the missing Xpert result dates from machine data from GeneXpert machines at testing hubs. For patients with microbiologically confirmed TB who are missing the smear or Xpert result date, the date of treatment initiation will be used as the date of diagnosis.

- ***Time-to-treatment***: Number of days from *date enrolled* to treatment start date entered into NTLP Treatment register if treatment start date is within six months of enrolment.

Primary Outcome

Outcome	Numerator	Denominator
Number treated for microbiologically-confirmed TB within 14 days of date enrolled.	Number diagnosed and time-to-treatment within 14 days	None

Secondary Outcomes

A. Testing		
**Outcome**	**Numerator**	**Denominator**
Number referred for TB testing	Number referred for testing	None
Proportion completing testing	Number completing testing	Number enrolled
**B. Diagnosis**		
**Outcome**	**Numerator**	**Denominator**
Number diagnosed with microbiologically-confirmed TB*	Number diagnosed	None
Proportion diagnosed with microbiologically-confirmed TB*	Number diagnosed	Number enrolled
Number with RIF resistance detected by Xpert/number with RIF resistance confirmed by culture*	Number with RIF resistance detected by Xpert/number with RIF resistance confirmed by culture	None
Proportion with RIF resistance detected by Xpert/proportion with RIF resistance confirmed by culture*	Number with RIF resistance detected by Xpert/number with RIF resistance confirmed by culture	Number enrolled
Time to microbiologically-confirmed TB**	Time-to-diagnosis if microbiologically-confirmed TB	None
**C. Treatment**		
**Outcome**	**Numerator**	**Denominator**
Number treated for TB*	Number treated	None
Proportion treated for TB*	Number treated	Number enrolled
Number treated for microbiologically-confirmed TB*	Number diagnosed AND treated	None
Proportion treated for microbiologically-confirmed TB*	Number diagnosed AND treated	Number enrolled
Proportion with microbiologically-confirmed TB treated*	Number diagnosed AND treated	Number diagnosed
Time-to-treatment of microbiologically-confirmed TB	Time-to-treatment if microbiologically-confirmed TB and treated	None
**D. Follow-up**		
**Outcome**	**Numerator**	**Denominator**
Number with microbiologically-confirmed TB completing treatment	Number diagnosed AND completing treatment	None
Proportion with microbiologically-confirmed TB completing treatment	Number diagnosed AND completing treatment	Number diagnosed AND treated
Number who died within 6 months	Number died	None

Proportion who died within 6 months***	Number died	Number enrolled
Number who died within 6 months	Number diagnosed AND died	None
Proportion who died within 6 months***	Number diagnosed AND died	Number diagnosed
Number who died within 6 months	Number treated AND died	None
Proportion who died within 6 months***	Number treated AND died	Number treated

*Outcome will be assessed within 1 day and within 14 days of enrolment. One day was chosen because the intervention focuses on same-day diagnosis and treatment. 14 days was chosen because the diagnostic process could take 7-10 days in the control arm depending on the frequency of sample transport to Xpert testing sites.
**For the control arm, missing Xpert result dates will be recorded as the next scheduled date of sputum transportation from the health center to the GeneXpert facility (results from the previous batch of testing are returned when the next batch of samples are picked up). For the intervention arm, missing Xpert result dates will be recorded as the day following sample collection.
***Vital status will be assessed between 4-9 months from enrolment

3.5 Sample Size

Sample size

Based on pre-randomization data collected from January to December 2017, we estimate 5360 patients will be enrolled across 20 study sites over the 18-month enrolment period. Other study intervals ranging from 12 to 24 months are considered.

Justification

Original detectable effect size estimates: The study is based on the health center being the unit of randomization and aims to demonstrate the superiority of the intervention arm. The sample size calculation uses formulae appropriate for cluster-randomized trials with a parallel design and stratified and/or restricted randomization, including the addition of one extra cluster per arm to allow for the loss of degrees of freedom due to stratification to obtain conservative estimates.[7] The original detectable effect size estimates were based on the outcome being the proportion of patients treated for microbiologically-confirmed TB within two weeks of referral for sputum-based testing. A type I error of 5% and power of 90% was assumed. Pre-randomization data collected from January to December 2017 from the 20 selected trial sites in Uganda suggested that the average proportion of patients referred for TB evaluation who initiate treatment for active TB within two weeks would be 6.7%, and the coefficient of variation (k) between clusters is 0.36, with a harmonic mean of 268 patients enrolled at each health center cluster during the 18-month enrolment period. Based on these assumptions and assuming a type I error of 5%, we will have 90% power to detect a 6% or greater absolute increase in the outcome proportion in the intervention arm (see **Table 1**). Table 1 also shows detectable absolute effect sizes with varying power (80%, 85%, 90%) during various trial time intervals and respective average cluster size estimates.

The minimum detectable effect size is based on published literature, our preliminary data demonstrating the increased sensitivity of Xpert Ultra vs. smear microscopy (95% vs. 40%), and reduced pre-treatment loss to follow-up expected in the intervention vs. control arms due to rapid, onsite molecular testing (7% vs. 30%). When applying these parameters to a population of 1000 individuals with TB prevalence of 10%, we would expect 95 patients to be diagnosed with TB and 88 to start treatment within 14 days in the intervention arm, and 40 patients to be diagnosed with TB and 28 to start treatment within 14 days in the control arm. Thus, the expected difference in the primary outcome proportion would be 8.8% vs. 2.8%, or 6%.[8]

Table 1. Absolute difference in the proportion of clinic attendees initiating TB treatment within 14 days of initial sputum submission, among those referred for TB testing

Average cluster size	Effect size (10 clusters per arm, k=0.36)		
	80% Power	85% Power	90% Power

178 (*12 months*)	5.9%	6.5%	7.4%
223 (*15 months*)	5.7%	6.3%	7.1%
268 (*18 months*)	5.5%	6.1%	6.9%
312 (*21 months*)	5.4%	6.0%	6.7%
357 (*24 months*)	5.3%	5.9%	6.6%

Figure 1. Effect size by cluster size. Number of clusters per arm is 10, *k*=0.36.

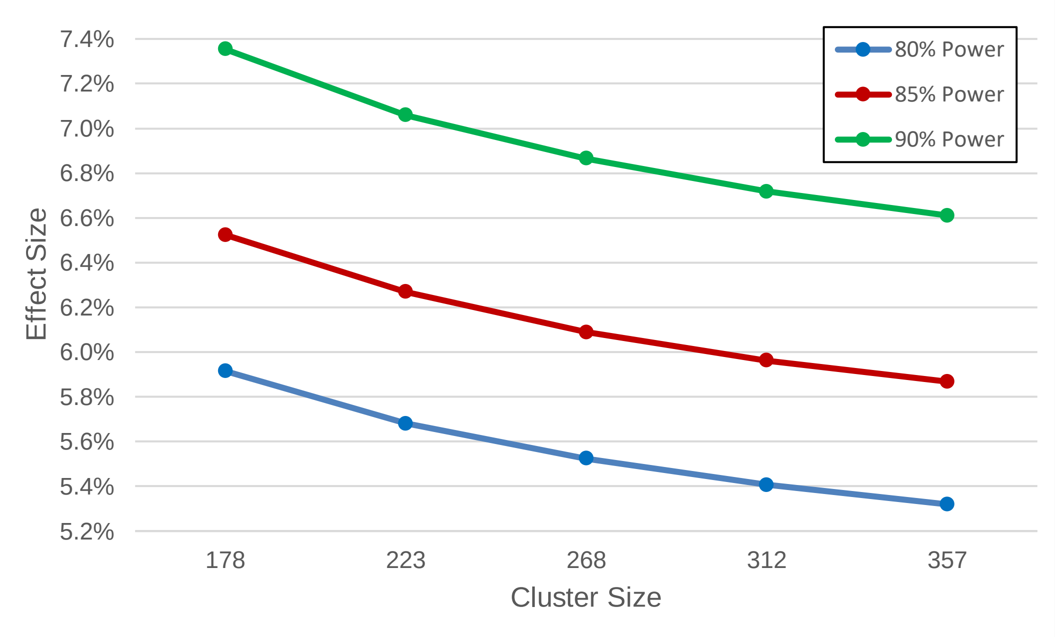

The primary outcome was changed to a count of the number of adults diagnosed and treated with TB at the cluster-level. The new primary outcome better reflects the intended effect of the multi-component intervention to drive more people who present to health facilities through the entire TB diagnostic evaluation cascade of care, including being screened for TB, tested for TB (if screen-positive), diagnosed with TB (if tested) and initiated on treatment (if diagnosed). Process re-design is expected to help improve the number screened and tested. Onsite molecular testing is expected to increase the numbers diagnosed (increased sensitivity) and treated (faster turn-around time and reduced workload). Performance feedback is expected to maintain improvement along each step of the cascade. Thus, the intervention is expected to increase both the number of patients entering and completing the cascade of care.

Revised detectable effect size estimates: The revised detectable effect size estimates are for the outcome being the number of patients treated for microbiologically-confirmed TB within two weeks of referral for sputum-based testing. We first used 10 months of pre-intervention period data across all 20 clinics to assess 1) the geometric mean number of patients diagnosed and treated for TB within 14 days (new primary outcome), the natural log (ln) of the geometric mean and 3) the standard deviation of the natural log at control sites, intervention sites and overall. For the pre-intervention period, the intervention clinics have a lower geometric mean number of clinic attendees who were diagnosed with TB and started on TB treatment within 14 days compared with the control arm (table 2).

Table 2: summary data from the baseline period

	Geometric mean	Mean of ln outcome	SD of ln outcome
Control	14.70	2.69	0.23

Intervention	6.49	1.87	0.32

We then estimated the detectable effect size (expressed as a geometric mean ratio). The calculations assume the same parameters as for the original primary outcome (10 clinics/arm, 18-month trial duration to achieve geometric mean of 268 patients/cluster in control and intervention arms). Table 3 below shows the detectable effect size assuming within-arm SD of ln outcome of 0.2 to 0.3 and power of 80-90%. The detectable effect sizes shown below are conservative (calculations do not take into account the baseline differences between arms in the new primary outcome and are based on 10-months rather than full 24-months of pre-intervention data). Final calculations will be repeated once baseline data is fully entered and available for analysis.

Table 3: Detectable effect sizes

SD of ln outcome	Geometric Mean Ratio of the new primary outcome (intervention vs control)	
	80% power	90% power
0.2	1.30	1.36
0.3	1.49	1.58

We believe a GMR of 1.30-1.58 is a reasonable detectable effect size. Xpert MTB/RIF is twice as sensitive as smear microscopy (double the number of confirmed TB cases) and we expect onsite testing to reduce pre-treatment loss to follow-up by at least half (from 30% to <15%).

3.6 Blinding

The trial will be open-label for participants and researchers, as blinding of the assigned intervention is not feasible given intervention implementation at the health center level. Staff, however, will be blinded to the intervention allocations through the use of codes to identify clusters rather than health center names. Where possible, the investigators and study staff will be masked to ongoing aggregated data by study. The trial statistician and data manager will have access to the aggregate data. Some site-level data will be used for performance feedback to sites in the intervention arm; this data will not be a comparison to baseline sites across study arms.

4. Randomization

4.1 Sequence Generation

A random allocation sequence will be generated by STATA 14 (StataCorp LP, College Station, TX). Randomisation of clusters to the intervention or control arm was conducted using stratification and restriction. Stratified randomisation was used to help reduce between-cluster variability and achieve balance at baseline. Using data from 2017 on the percentage of adult clinic attendees being investigated for TB who started TB treatment within 14 days, the clusters were stratified into two equal-sized groups – "low" and "high" percentages of this outcome based on the median. Restricted randomisation was used to help ensure balance for key factors. Variables we restricted on were; year cluster enrolled in project (2017 vs. 2018); sputum transport frequency to Xpert hub; distance to Xpert hub; number of patients evaluated for TB; percentage starting TB treatment within 14 days; urban vs. rural health clinics; HIV prevalence; number of facilities per district. For these restriction criteria the overall proportion of unacceptable allocations was calculated and the the validity assessed using the validity matrix.[9] From a total of 63,504 possible randomisations based on two equal-sized strata, applying the restriction criteria resulted in 11,392 randomisations; one of these was chosen at random.

4.2 Allocation concealment mechanism

Allocation concealment was used to prevent selection bias by concealing from the research staff the allocation sequence until assignment had occurred.

4.3 Implementation

The trial statistician generated the random allocation assignments, and clusters were enrolled by local study staff. Eligible individual patients will be included in clusters through complete enumeration. District health officers provide administrative authority and consent to health center participation prior to randomization. Consent is documented in a signed Memorandum of Understanding (MOU) between the district and the National Tuberculosis and Leprosy Control Program (NTLP), endorsed by the Chief Administrative Officer, the District Health Officer, and the Program Manager for NTLP. A copy of the agreement is provided to the facility in-charge at each health center.

A public ceremony for randomization was held in Kampala, Uganda. Facility in-charges, district health officers, and representatives from NTLP attended.

4.4 Statistical Methods

4.4.1 General analyses principles

We will calculate and compare all effectiveness outcomes for the two trial arms using prespecified analyses following the intention-to-treat principle. Statistical analysis methods to determine intervention effect on outcomes will be used that are appropriate for CRTs with a small number of clusters. Analysis of cluster-level summaries is based on a total of 20 clusters (10 per arm), each comprised of one health center, and each being given equal weight. Effect estimates will be reported with 95% confidence intervals, and an associated P-value.

4.4.2 Baseline data

Baseline variables (from the 12 month period before 22 October 2018 and socio-demographic data from 12 month period after 22 October 2018) will be cross-tabulated to check for appropriate balance between study arms and to characterize the study population using summary statistics both at cluster and individual levels (Annex **Table 1, 2**).

Cluster (*i.e.* health center) level data will be collapsed across study arms and compared using mean, median and range with standard deviations and/or interquartile ranges as appropriate. The basis of this analysis will be data on demographics, health center and laboratory infrastructure, organizational hierarchy, Xpert referral network, and TB testing and treatment collected during site assessment, recruitment, and guidelines training.

Individual level data for all patients eligible for analysis will also be collapsed across study arms and compared using mean, median and range with standard deviations and/or interquartile ranges as appropriate. These individual factors include age, sex, HIV status, and ART status.

We will summarize data obtained during the pre-randomization period for the cohort of health centers and patients who are being enrolled in the study and randomized to a trial arm (Annex table 1).

We will also summarise of numbers referred for TB testing in intervention and control arms (collapsed over cluster) in the 12 month period after 22 October 2018 (Annex Table 3).

Descriptive data between control and intervention arms will be presented in tabular format in lieu of formal statistical comparison or significance tests, and will be reviewed to confirm comparability and identify any substantial imbalances. Adjusted effect estimates will be calculated by study arm to account for imbalance of covariates which are risk factors for the outcome of interest. Losses in degrees of freedom due to cluster-level covariate adjustments will be accounted for in all calculations.

4.4.3 Primary analysis of primary outcome

Analysis will be conducted at the cluster (clinic)-level. The outcome is the count of the number treated for microbiologically-confirmed TB within two weeks of referral for sputum-based testing at the clinic-level. Initially Poisson regression will be used, with an offset at the clinic level of the total number of days the clinic contributes individuals to the study (starting from 22 October 2018). Evidence for over-dispersion will be assessed and if over-dispersion is detected then negative binomial regression will be used, using the same offset. Either model will adjust for randomisation strata and the count of microbiologically-confirmed TB cases that initiated on treatment within 14 days in the 12 month period before 22 October 2018. The exact functional form of this covariate will be identified through fractional polynomials. Further adjustment for clinic-level factors that show imbalance will be explored, though the number of adjustment factors will be limited as all are at the clinic-level. See Annex tables 4a and 4b, and 5a.

Xpert Ultra cartridges will be used in our intervention (see Outcomes Definitions Section 3.4). Two sensitivity analyses will be performed reclassifying 1) all trace-positive results as negative and 2) trace-positive results in HIV negative patients as true positive (even if not confirmed by a second positive test) to determine the impact of Ultra on the primary outcome. We expect to see trace-positive results in between 10-20% of all positive Xpert Ultra results, irrespective of study arm. Therefore, sensitivity analyses will only be done if the ratio of trace-positive results to all positive Xpert Ultra results is similar in both trial arms.

No sub-group analyses for strata defined at the individual-level (e.g., sex and HIV status) will be conducted as the primary outcome analyses are conducted at the cluster-level.

4.4.4 Analysis of secondary outcomes

Secondary outcomes are reported as either counts, proportions, or time to event (continuous outcome), and are derived from data collected across routine data sources.

All analyses will adjust for randomisation strata. Intervention effect estimates will be reported for analyses (1) adjusting for randomisation strata only and (2) adjusting for randomisation strata and individual-level (proportions and time to event only) and clinic-level factors considered imbalanced, as outlined below. See Annex table 5b.

4.4.4.1 Count outcomes:

The analytic approach will be the same as described for the primary outcome The outcome is the count of the respective outcome at the clinic-level. Analysis will be conducted at the cluster (clinic)-level. Either Poisson or negative binomial regression will be used, depending on over-dispersion. Adjustment will be made for the clinic-level count of the outcome using data in the 12 month period before 22 October 2018. The exact functional form of this covariate will be identified through fractional polynomials.

4.4.4.2 Proportions

The analysis will give each cluster equal weight. The overall risk for each cluster will be calculated and shown, by strata and arm. A log transformation (where necessary) will be applied to the risk for each cluster. If a cluster has no events, 0.5 event will be added to all clusters in order that the log transformation can be conducted. The mean and standard deviation of these log risks will be used to obtain the geometric mean (GM) and associated 95% CI for each arm of the study. The risk ratio is estimated using linear regression of the log risks on stratum and arm. An approximate standard error for the difference in log (GM) between arms is obtained based on the residual mean square from a two-way analysis of variance (ANOVA) of the cluster log risk on stratum, study arm and the interaction between stratum and study arm. The 95% CI is calculated from this standard error, using a t-statistic with 16 degrees of freedom. This will be the primary analysis.

Limited adjustment for individual level factors is possible; age, sex, HIV status, and ART status. Adjustment, using a complete case analysis, will only be done if data completeness for the variable is more than 80% in both trial arms and imbalance is observed (not assessed through a P-value).Logistic regression will be used to adjust for any baseline imbalance at the individual level, adopting a two-stage approach. The regression model will include terms for the adjustment factors (individual level) and strata, but not study arm. For each cluster the fitted model will be used to obtain the ratio of observed to expected (O/E) events, and a log transformation will be applied to this ratio, where appropriate.

Linear regression of the log (O/E) on stratum and arm (2-way ANOVA) will be used to estimate the risk ratio. The variance for the ratio of mean O/E is calculated from the residual mean square from an ANOVA of log(O/E) on stratum, arm and the interaction between stratum and arm. The 95% CI is calculated from this variance, using a t-statistic with 16 degrees of freedom

If imbalance is observed for the equivalent cluster-level outcome in the 12 month period before 22 October 2018, adjustment at the second stage described above will be conducted, with appropriate adjustment for the degrees of freedom for the intervention effect. The exact functional form of this cluster-level covariate will be identified through fractional polynomials.

4.4.4.1 *Time to event (continuous outcome)*

The analysis will give each cluster equal weight. The arithmetic and geometric mean for the continuous outcome of time to event (amongst all those experiencing the event) will be calculated for each cluster and shown, by strata and arm. A log transformation of the individual-level time to event will likely be necessary as the times to event may be positively skewed, resulting in geometric means. Clusters where no individuals contribute time to event outcome data will be excluded from the analysis.
ANOVA will be used to calculate either the difference in means between arms or geometric mean ratio, depending on whether a log transformation was used. This will be the primary analysis.

A two-stage approach, similar to as described above, will be used to adjust for confounders at the individual level. Limited adjustment for individual level factors is only possible; age, sex, HIV status, and ART status. Adjustment will only be done if data completeness for the variable is more than 80% in both trial arms and imbalance is observed (not assessed through a P-value). If imbalance is observed for the equivalent cluster-level outcome in the 12 month period before 22 October 2018, adjustment at the second stage described above will be conducted, with appropriate adjustment for the degrees of freedom for the intervention effect. The exact functional form of this cluster-level covariate will be identified through fractional polynomials.

4.4.5 Sub-group analyses for secondary outcomes

Pre-specified sub-group analyses for a subset of secondary outcomes will be conducted to assess whether the effect of the intervention differs between sub-groups. The sub-groups are (1) HIV status at enrolment (self-reported, laboratory test resulted as noted on primary data sources, or through pre-ART and ART register data extraction) defined as positive or negative, and (2) sex (male or female). The effect of the intervention will be estimated for each sub-group, and P-values for effect modification, appropriate for individual-level covariates, will be reported.[10] These sub-group analyses will be conducted for the following secondary outcomes: proportion completing testing; proportion of patients referred for TB testing who are diagnosed with microbiologically-confirmed TB within 14 days; proportion of patients with microbiologically-confirmed TB who are treated within 14 days of referral for sputum-based testing; and the proportion who died within 6 months. See Annex table 5c. Results from subgroup analyses will also be summarised as forest plots.

Annex tables

Table 1. Summary of baseline study variables at the cluster-level or using data for the 12 month period before 22 Oct 2018

		Intervention	Control
Year of enrolment into project	*2017 or 2018*		
Distance to Xpert hub	*Median (IQR)*		
Sputum transport frequency to Xpert hub	*Once per week or ≥2 times per week*		
Total number referred for TB testing*	#		
Average number referred for TB testing, across clusters*	Harmonic mean		
Region	*Central or Eastern, % (n/N)*		
District	*% per district*		
Geographical area	*Urban or rural, % (n/N)*		
Population of sub-county	#		
Patients undergoing TB testing who are diagnosed with and treated for TB within 14 days*	*(< 6.2% or ≥ 6.2%)*		
HIV prevalence among TB patients*	*% (n/N)*		

* in the 12 month period before 22 October 2018

Table 2. Summary of demographic variables among those referred for TB testing* (collapsed over cluster)

		Intervention	Control
Total number referred for TB testing	#		
Average number referred for TB testing, across clusters	Harmonic mean		
Missing data for sex	% (n/N_{total})		
Female	% $(n/N_{non\text{-}miss})$		
Missing data for age	% (n/ N_{total})		
Age	*Median (IQR)*, $N_{non\text{-}miss}$		
HIV status known:	% (n/N_{total})		
HIV-positive	% $(n/N_{non\text{-}miss})$		
ART status known:	% $(n/N_{total\ HIV+})$		
ART prevalence	% $(n/N_{non\text{-}miss\ HIV+})$		

* in the intervention period after 22 October 2018

Table 3: Summary of numbers referred for TB testing* in intervention and control arms (collapsed over cluster) using data from record review and facility follow up.

		Intervention	Control
Xpert result	*Negative*<br>*Positive*<br>*Unknown*		
Semi-Quantitative result	*High*<br>*Medium*<br>*Low*<br>*Very Low*<br>*Trace*		
RIF result	*Negative*<br>*Positive*<br>*Indeterminate/Invalid/No Result/Error*<br>*Unknown/No Result*		
MDR status	*Yes, confirmed positive by DST*<br>*No, confirmed negative by DST*<br>*Unknown*		
Smear result	*Negative*<br>*Positive*<br>*Unknown*		
Treatment status	*Yes, on treatment*<br>*No, not on treatment*<br>*Unknown*		
Treatment category	*Category 1- 2RHZE/6EH*<br>*Category 1- 2RHZE/4RH*<br>*Category 2-2SRHZE/1RHZE/5RHE*<br>*Category 3-2RHE/4RH*<br>*Other*		
Treatment outcome	*Cured*<br>*Completed*<br>*Failure (smear positive)*<br>*Died*<br>*Transferred out*<br>*Lost to Follow up*		

* in the period after 22 October 2018

Table 4a: Primary outcome - summary of number treated for microbiologically-confirmed TB within 14 days of referral for sputum-based testing and total number of months clinic contributes individuals to the study, by control and intervention arm

	Control arm		Intervention arm	
Cluster	# diagnosed and treated within 14 days	total number of months the clinic contributes individuals to the study	# diagnosed and treated within 14 days	total number of months the clinic contributes individuals to the study
1				
2				
3				
4				
5				
6				
7				
8				
9				
10				
Geometric Mean				

Table 4b: Primary outcome, summary of number treated for microbiologically-confirmed TB within 14 days of referral for sputum-based testing and total number of months clinic contributes individuals to the study, by control and intervention arm for the 12 month period before 22 October 2018

	**Control arm**		**Intervention arm**	
Cluster	# diagnosed and treated within 14 days	total number of months the clinic contributes individuals to the study	# diagnosed and treated within 14 days	total number of months the clinic contributes individuals to the study
1				
2				
3				
4				
5				
6				
7				
8				
9				
10				
Geometric Mean				

Table 5a: Primary outcome: summary of number treated for microbiologically-confirmed TB within 14 days of referral for sputum-based testing and, adjusted risk ratio (95% confidence intervals), and associated P-values

Primary outcome	I: #, GM	C: #. GM	Adjusted RR* (95% CI)	P-value
Primary analysis				
Sensitivity analysis 1				
Sensitivity analysis 2				

I Intervention ; C control, GM geometric mean

* adjusted for randomisation strata and count of microbiologically-confirmed TB cases that initiated on treatment within 14 days in the 12 month period before 22 October 2018. The RR and 95% CI, adjusting for randomisation strata only is XX (95% CI yy to zz).

Sensitivity analyses are: reclassifying 1) all trace-positive results as negative and 2) trace-positive results in HIV negative patients as true positive (even if not confirmed by a second positive test) to determine the impact of Ultra on the primary outcome.

Table 5b: Secondary outcomes: unadjusted and adjusted risk ratio (95% confidence intervals) and associated P-values

Secondary outcomes		I:	C:	RR (95% CI)	P-value	Adjusted RR (95% CI)	P-value
A. Testing							
Number referred for TB testing	# GM						
Proportion completing testing	% (n/N) GM %						
B. Diagnosis							
Number diagnosed with microbiologically-confirmed TB (by day 1)	# GM						
Number diagnosed with microbiologically-confirmed TB (by day 14)	# GM						
Proportion diagnosed with microbiologically-confirmed TB (by day 1)	% (n/N) GM %						
Proportion diagnosed with microbiologically-confirmed TB (by day 14)	% (n/N) GM %						

Number with RIF resistance detected by Xpert/ number with RIF resistance confirmed by culture (by day 1)	#<br>GM						
Number with RIF resistance detected by Xpert/ number with RIF resistance confirmed by culture (by day 14)	#<br>GM						
Proportion with RIF resistance detected by Xpert/proportion with RIF resistance confirmed by culture (by day 1)	% (n/N)<br>GM %						
Proportion with RIF resistance detected by Xpert/proportion with RIF resistance confirmed by culture (by day 14)	% (n/N)<br>GM %						
Time to microbiologically-confirmed TB	#<br>GM						
C. Treatment							
Number treated for TB (by day 1)	#<br>GM						
Number treated for TB (by day 14)	#<br>GM						
Proportion treated for TB (by day 1)	% (n/N)<br>GM %						
Proportion treated for TB (by day 14)	% (n/N)<br>GM %						
Number treated for microbiologically-confirmed TB (by day 1)	#<br>GM						
Number treated for microbiologically-confirmed TB (by day 14)	#<br>GM						
Proportion treated for microbiologically-confirmed TB (by day 1)	% (n/N)<br>GM %						
Proportion treated for microbiologically-confirmed TB (by day 14)	% (n/N)<br>GM %						
Proportion with microbiologically-confirmed TB treated (by day 1)	% (n/N)<br>GM %						
Proportion with microbiologically-confirmed TB treated (by day 1)	% (n/N)<br>GM %						
Time-to-treatment of microbiologically-confirmed TB	GM						
D. Follow-up							

Number with microbiologically-confirmed TB completing treatment	#<br>GM						
Proportion with microbiologically-confirmed TB completing treatment	% (n/N)<br>GM %						
Number who died within 6 months	#<br>GM						
Proportion who died within 6 months	% (n/N)<br>GM %						

GM geometric mean; GM % geometric mean of the cluster-level proportions

Table 5c: Sub-group analyses for selected secondary outcomes: stratum-specific unadjusted and adjusted risk ratios (95% confidence intervals) and associated P-values for interaction

Secondary outcomes		I:	C:	RR (95% CI)	P-value	Adjusted RR (95% CI)	P-value	P-value for interaction
A. Proportion completing testing								
HIV-positive	% (n/N)							
HIV-negative	% (n/N)							
Male	% (n/N)							
Female	% (n/N)							
B. Proportion referred for TB testing who are diagnosed with microbiologically-confirmed TB (by day 14)								
HIV-positive	% (n/N)							
HIV-negative	% (n/N)							
Male	% (n/N)							
Female	% (n/N)							
C. Proportion of patients with microbiologically-confirmed TB who are treated within 14 days of referral for sputum-based testing								
HIV-positive	% (n/N)							
HIV-negative	% (n/N)							
Male	% (n/N)							
Female	% (n/N)							
D. Proportion who died within 6 months								
HIV-positive	% (n/N)							
HIV-negative	% (n/N)							
Male	% (n/N)							
Female	% (n/N)							

Figure 1: Comparison of TB diagnostic evaluation in the intervention and control arms

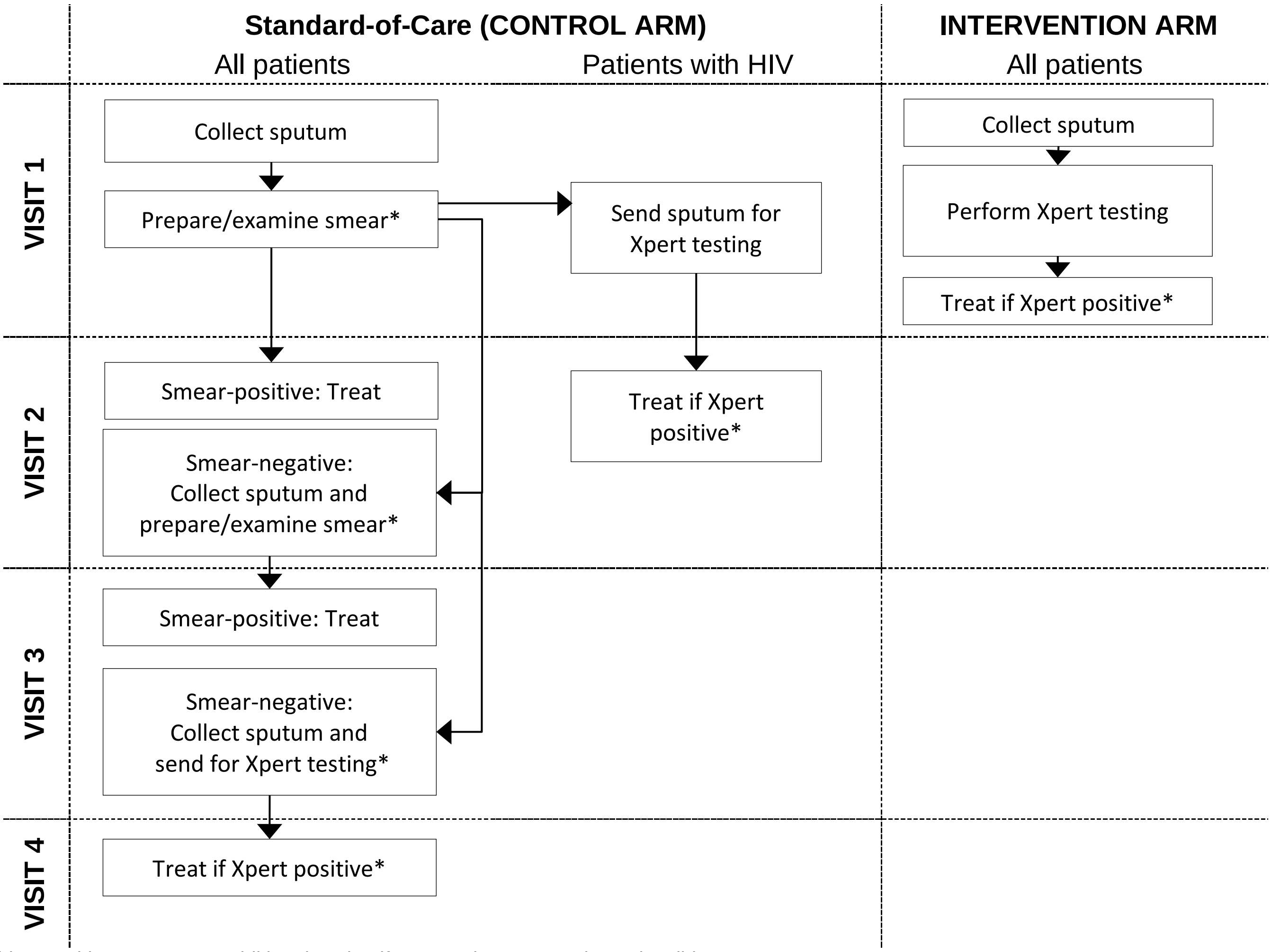

*Consider empiric treatment or additional testing if test results are negative or invalid

Figure 2: CONSORT diagram

CONSORT diagram will be constructed show individual and cluster-level data at baseline and follow-up and based on the CONSORT extension to CRTs.

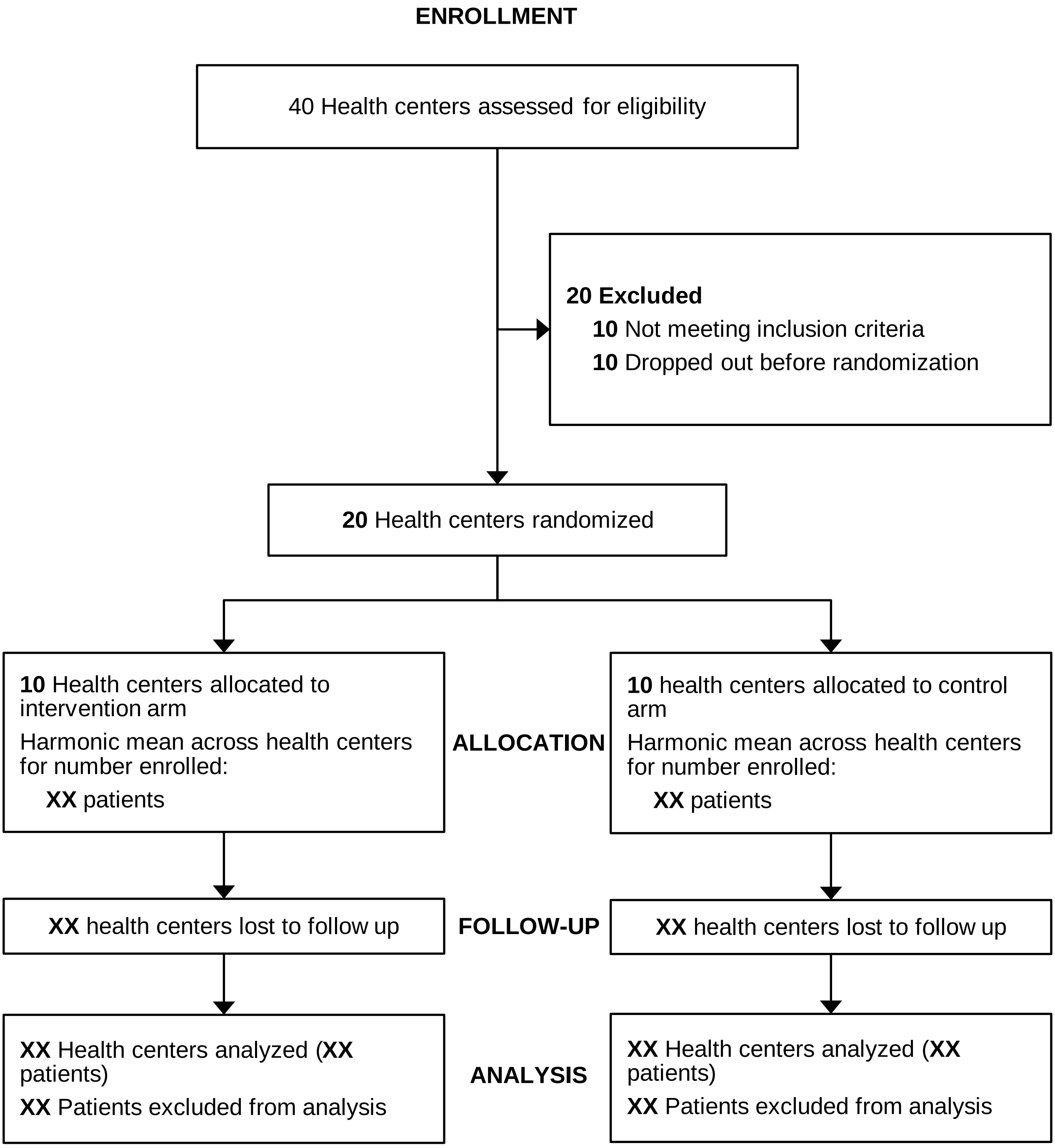

Post-hoc analyses

The following outcomes were not specified in the SAP but were included in the trial manuscript to ensure 1) each proportion outcome had a corresponding count outcome (or vice versa) and 2) every relevant step of the TB diagnostic evaluation cascade was represented (for those with microbiologically-confirmed TB).

Overall

- Number completing testing

Patients with microbiologically-confirmed TB

- Proportion diagnosed*

Subgroup analyses

- Number completing testing
- Number diagnosed with microbiologically-confirmed TB*
- Number treated for microbiologically-confirmed TB*
- Number treated for TB*
- Proportion with same-day diagnosis of microbiologically-confirmed TB
- Proportion with same-day treatment for microbiologically-confirmed TB
- Proportion treated for TB*

*Outcomes assessed within 1 day and within 14 days of initial sputum submission

References

1. Pinnock H, Barwick M, Carpenter CR, et al. Standards for Reporting Implementation Studies (StaRI): explanation and elaboration document. *BMJ Open.* 2017;7(4):e013318.
2. Campbell MK, Piaggio G, Elbourne DR, Altman DG, Group C. Consort 2010 statement: extension to cluster randomised trials. *BMJ.* 2012;345:e5661.
3. Ministry of Health (Republic of Uganda). Health Facilities Inventory. Department of Clinical Services. Kampala, Uganda 2012:140.
4. Uganda Bureau of Statistics. The National Population and Housing Census 2014 - Main Report. Kampala, Uganda 2016.
5. Uganda Bureau of Statistics. National Population and Housing Census 2014 - Provisional Results. Kampala, Uganda 2014.
6. World Health Organization. *Non-inferiority analysis of Xpert MTB/RIF Ultra compared to Xpert MTB/RIF.* Geneva: World Health Organization; 2017.
7. Hayes RJ, Moulton, L.H. *Cluster Randomized Trials.* Boca Raton: CRC Press; 2009.
8. Farr K, Nalugwa, T., Shete, P., Cattamanchi, A., Nantale, M., Ojok, C., Oyuku, D., Nabwire, S., Fielding, K., Joloba, M., Mugabe, F., Dowdy, D., Moore, D., Davis, J.L., Katamba, A. . Late Breaking Abstract - The tuberculosis diagnostic evaluation cascade of care at microscopy centers linked to Xpert MTB/RIF hubs in Uganda. *European Respiratory Journal.* 2017;50.
9. Moulton LH. Covariate-based constrained randomization of group-randomized trials. *Clin Trials.* 2004;1(3):297-305.
10. Cheung YB, Jeffries D, Thomson A, Milligan P. A simple approach to test for interaction between intervention and an individual-level variable in community randomized trials. *Trop Med Int Health.* 2008;13(2):247-255.

Table of Amendments

XPEL TB SAP

**Change**	**Date of change**
**Section 1.1**: We updated the aims section to reflect the aims listed in the protocol	November 21, 2019
**Section 2.3**: We added that trial reporting will follow the STaRI checklist based on feedback from one of the co-investigators.	November 21, 2019
**Section 2.3**: We specified that the SAP summarizes analyses for effectiveness outcomes only.	November 21, 2019
**Section 3.2**: We removed the exclusion criteria from this section and moved this text to "Outcomes" (Section 3.4).	November 21, 2019
**Section 3.4**: We added definitions for "eligible patent", "number died, among those with confirmed TB", and "number died, among all those eligible".	November 21, 2019
**Section 3.4**: We removed reference to GxAlert database (not used during the trial)	November 21, 2019
**Section 3.4**: We specified GeneXpert machine data can be used as a data source for the trial	November 21, 2019 (intervention)<br><br>March 4, 2020 (intervention and control)
**Section 3.4**: We specified that test results must be available within 6 months of enrollment to be considered in the trial analysis	November 21, 2019
**Section 3.4**: For the new mortality outcomes we added, we indicated in the footnotes of Table 1 that we will assess vital status between 4-9 months from enrollment.	November 21, 2019
**Section 3.5**: We included a justification for changing the primary outcome from a proportion to a count	November 21, 2019
**Section 3.5**: We corrected a typo about our geometric mean (updated to reflect what we had intended geometric mean: 268 patients/cluster).	November 21, 2019
**Section 4.4**: We indicated we would summarize the number of patients referred for TB testing (collapsed by cluster) in the 12-month period after the trial start date.	November 21, 2019
**Section 4.4**: We indicated we would only perform sensitivity analyses on trace positive results if the ratio of trace-positive results to all positive Xpert Ultra results was similar in both trial arms.	January 10, 2020
**Section 4.4**: We indicated when adjustment for individual-level characteristics will be done (if data completeness >80%).	November 21, 2019
**Section 4.4**: We clarified the language about for which variables subgroup analyses will be performed. We also indicated results from subgroup analyses will be summarized as forest plots.	November 21, 2019
**Appendix (Post-hoc Analyses)**: We added an appendix listing the post-hoc analyses that we conducted and included in the trial manuscript	August 6, 2020
**Section 3.4:** To improve clarity for reporting purposes, we updated the denominator for proportion outcomes to be "number enrolled" (i.e., the number evaluated for TB, which is our full study population).	July 12, 2021
