# Supplementary material for: Impact of a multicomponent strategy including decentralized molecular testing for tuberculosis on mortality: planned analysis of a cluster-randomized trial in Uganda
Source: eClinicalMedicine. 2024 Nov 26;78:102953. doi: 10.1016/j.eclinm.2024.102953 (PMC11629260; doi:10.1016/j.eclinm.2024.102953)
Supplement: Supplementary Figure and Tables [file mmc2.docx]

**Supplementary Appendix**

**Table of Contents**

| **Supplementary Methods** | **2** |
| --- | --- |
| **Procedures for vital status tracing** | **2** |
| **Additional detail regarding outcome definitions and analysis** | **2** |
| **Supplementary Tables and Figures** | **3** |
| **Table S1. Vital status ascertainment by health center** | **3** |
| **Table S2. Vital status outcome by method of follow-up** | **4** |
| **Table S3. Treatment initiation within 14 days of evaluation by trial arm and tuberculosis status** | **4** |
| **Table S4. Patient and cluster-level demographic and clinical characteristics for six-month mortality risk outcome** | **5** |
| **Figure S1. Individuals, randomization, and vital status follow-up for six-month mortality risk outcome** | **6** |
| **Table S5. Unadjusted and adjusted mortality ratios for risk outcome, overall and by subgroup** | **6** |
| **Figure S2. Adjusted mortality rate ratios and six-month mortality risk ratios** | **7** |
| **Table S6. Cluster-level mortality rate and six-month mortality risk outcomes** | **8** |
| **Table S7. Adjusted mortality rate difference and six-month mortality risk difference** | **9** |
| **Table S8. Sensitivity analyses for mortality rate and six-month mortality risk outcomes** | **10** |

**Supplementary Methods**

**Procedures for vital status tracing**

1. To obtain vital status for patients who initiated tuberculosis (TB) treatment, the TB treatment registers for each health center were referenced regularly by study staff during quality assurance site visits. For all other patients, phone calls or home visits were attempted. Confirmation of vital status by next of kin was accepted.
2. Lists of participants eligible for 6-month vital status follow-up were generated monthly.
3. The original protocol instructed the study team to call three times if a phone number was available, then to proceed to a home visit if phone calls were unsuccessful.
4. A paper list of those who required a home visit was provided to community health staff for each of the health centers. Some patients received both a phone call and a home visit. The paper lists had to be physically transported to the community health staff, and multiple attempts may have been made to locate the patient, during which time study staff may have continued to try reach the patient by phone.
5. The protocol for follow-up was enhanced as the study team found good yield with additional phone calls; a common reason cited by patients was that their phones were not always charged. Three additional calls were added, at which point there was a noticeable drop off in yield and no further calls were added to the protocol. The final phone outcome and date were captured in the study database.
6. Participants on treatment may also have received a phone call or home visit. If there was no information available in the TB treatment register nine months after treatment initiation, the participant was added to the phone call list and then the home visit list, if phone calls were unsuccessful. In the interim, the register data may have been updated. For the reasons described, multiple sources of vital status outcome data were available for some participants.

**Additional detail regarding outcome definitions and analysis**

**Mortality rate:** If the exact day of death was not known, study staff were instructed to enter the first of the month. For this reason, for patients with a date of death on the first of the month, the date of death was reassigned to the fifteenth of the month. As described in the manuscript text, for patients known to have died but with an unknown death date, the death date was assigned to be the midpoint between the date last known to be alive (smear or Xpert date) and the date the individual was first known to have died, based on a home visit, phone call, or the TB treatment register. For patients with multiple sources of outcome data and discrepant dates of death documented, the home visit data were prioritized if available, then phone call data were given next priority. For patients marked as having died but subsequently marked as alive by additional follow-up, the same prioritization scheme was applied with home visit then phone call then register data referenced.

**Cluster-level analysis**: Analyses excluded those with unknown HIV status and unknown vital status outcome. Patients known to be alive but with no valid date associated with the follow-up outcome were also excluded. For clusters with zero outcomes in sub-group analyses, we added +0·5 to all numerators.

**Supplementary tables and figures**

***Table S1. Vital status ascertainment by health center (N=9563)***

|  | **Known vital status for mortality rate outcome** | **Percent** | **Unknown vital status for mortality rate outcome** | **Percent** |
| --- | --- | --- | --- | --- |
| **Intervention** | | | | |
| Health Center 1 | 609 | 92·1% | 52 | 7·9% |
| Health Center 2 | 345 | 86·7% | 53 | 13·3% |
| Health Center 3 | 628 | 83·6% | 123 | 16·4% |
| Health Center 4 | 633 | 77·2% | 187 | 22·8% |
| Health Center 5 | 149 | 76·0% | 47 | 24·0% |
| Health Center 6 | 632 | 99·1% | 6 | 0·9% |
| Health Center 7 | 341 | 97·7% | 8 | 2·3% |
| Health Center 8 | 212 | 77·1% | 63 | 22·9% |
| Health Center 9 | 560 | 86·2% | 90 | 13·8% |
| Health Center 10 | 447 | 83·6% | 88 | 16·4% |
| **Routine care** | | | | |
| Health Center 11 | 683 | 90·2% | 74 | 9·8% |
| Health Center 12 | 494 | 84·3% | 92 | 15·7% |
| Health Center 13 | 386 | 89·2% | 47 | 10·8% |
| Health Center 14 | 760 | 96·7% | 26 | 3·3% |
| Health Center 15 | 163 | 70·9% | 67 | 29·1% |
| Health Center 16 | 503 | 88·1% | 68 | 11·9% |
| Health Center 17 | 161 | 94·2% | 10 | 5·8% |
| Health Center 18 | 241 | 90·3% | 26 | 9·7% |
| Health Center 19 | 238 | 95·2% | 12 | 4·8% |
| Health Center 20 | 228 | 95·4% | 11 | 4·6% |
| **Total** | **8413** | **88·0%** | **1150** | **12·0%** |

***Table S2. Vital status outcome by method of follow-up (N=8413)***

| **Follow-up method** | **Patients confirmed alive***  **N (%)** | **Patients confirmed to have died**†  **N (%)** |
| --- | --- | --- |
| **Home visit** | 4894 (60·4) | 177 (58·2) |
| **Phone call** | 2828 (34·9) | 84 (27·6) |
| **Treatment register** | 387 (4·8) | 43 (14·1) |
| **Total** | 8109 | 304 |

Footnote:

*Based on vital status definition applied for the mortality rate outcome

†Five patients were confirmed to have died but the date of death was outside the 18-month follow-up window (one confirmed by home visit and four by phone call).

***Table S3. Treatment initiation within 14 days of evaluation by trial arm and tuberculosis status (N=8413)***

| **Treatment Initiated** | **Intervention** | | **Routine care** | |
| --- | --- | --- | --- | --- |
|  | **No confirmed TB*** | **Confirmed TB*** | **No confirmed TB** | **Confirmed TB** |
| **Yes** | 125 (3·0) | 325 (89·0) | 109 (3·1) | 211 (72·8) |
| **No** | 4066 (97·0) | 40 (11·0) | 3458 (96·9) | 79 (27·2) |
| **Total**† | 4191 | 365 | 3567 | 290 |

Footnote:

*Tuberculosis (TB) status is defined as either confirmed or no confirmed TB within the 6 months post-TB diagnostic evaluation

†Includes those with a known vital status based on the definition applied for the mortality rate outcome

***Table S4. Patient and cluster-level demographic and clinical characteristics for six-month mortality risk outcome***

|  | Overall  N = 9563 | Known vital status for mortality risk outcome  N = 8116 | Unknown vital status for mortality risk outcome  N = 1447 | p-value* |
| --- | --- | --- | --- | --- |
|  | N | N (%) | N (%) |  |
| Trial arm |  |  |  | 0·51 |
| Intervention | 5273 | 4421 (83·8) | 852 (16·2) |  |
| Routine care | 4290 | 3695 (86·1) | 595 (13·9) |  |
| Sex |  |  |  | 0·27 |
| Female | 5709 | 4814 (84·3) | 895 (15·7) |  |
| Male | 3854 | 3302 (85·7) | 552 (14·3) |  |
| Age |  |  |  | 0·13 |
| 18-29 | 2470 | 2072 (83·9) | 398 (16·1) |  |
| 30-39 | 2188 | 1887 (86·2) | 301 (13·8) |  |
| 40-49 | 2038 | 1743 (85·5) | 295 (14·5) |  |
| 50+ | 2867 | 2414 (84·2) | 453 (15·8) |  |
| HIV status |  |  |  | 0·12 |
| Positive | 4190 | 3608 (86·1) | 582 (13·9) |  |
| Negative | 5373 | 4508 (83·9) | 865 (16·1) |  |

Footnote:

*Adjusted for clustering by health center

***Figure S1. Individuals, randomization, and vital status follow-up for six-month mortality risk outcome***

**
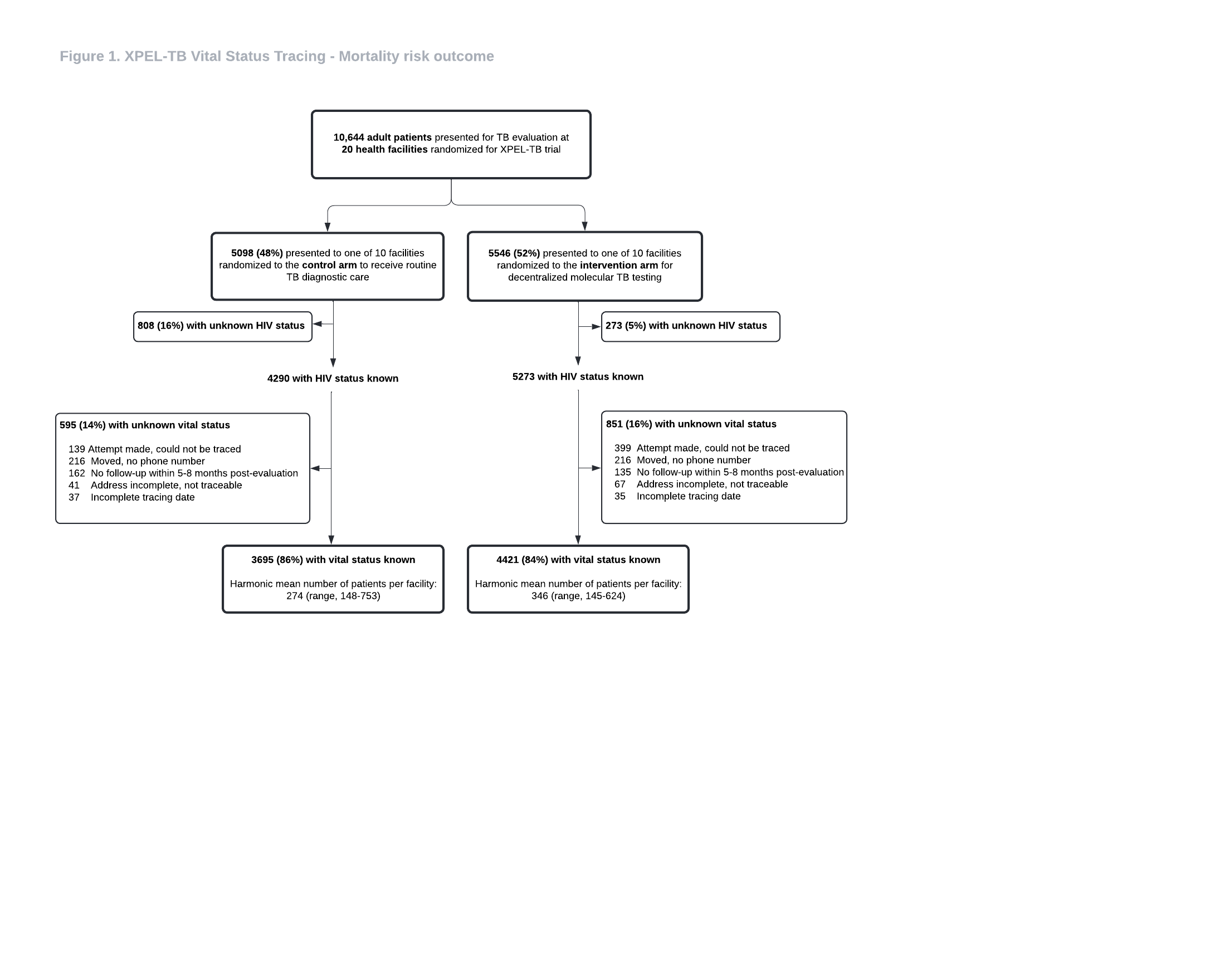
**

Footnote:

Abbreviations: TB=tuberculosis

***Table S5. Unadjusted and adjusted mortality ratios for risk outcome, overall and by subgroup (N=8116)***

|  | *Intervention* | | *Routine care* | | *Adjusted Risk Ratio*  *(95%CI) †* | | *p-value for interaction* |
| --- | --- | --- | --- | --- | --- | --- | --- |
|  | Deaths Observed/  N | Risk* (%) | Deaths Observed/  N | Risk* (%) | |  |  |
| Overall | 116 / 4421 | 2·45 | 123 / 3695 | 3·12 | | 0·77 (0·44—1·35) |  |
| HIV status |  |  |  |  | |  | 0·10 |
| Positive | 78 / 1935 | 3·81 | 59 / 1673 | 3·91 | | 0·97 (0·52—1·81) |  |
| Negative | 38 / 2486 | 1·50 | 64 / 2022 | 2·74 | | 0·56 (0·30—1·06) |  |
| Sex |  |  |  |  | |  | 0·28 |
| Female | 48 / 2576 | 1·45 | 63 / 2238 | 2·75 | | 0·68 (0·34—1·34) |  |
| Male | 68 / 1845 | 3·50 | 60 / 1457 | 3·62 | | 0·93 (0·50—1·74) |  |

Footnote:

*Unadjusted risks in the intervention and routine care arms are the geometric mean of the point estimates across the ten clusters in each group· The unadjusted risk ratio is 0·78 (0·44—1·39).

†Adjusted risk ratios compare the intervention vs. routine care arm and analysis was done at the cluster-level with adjustment for randomization strata (fixed effect, two levels), number of individuals treated for TB in the prerandomization period, and patient-level covariates (age, sex, and HIV status). Individuals with unknown HIV status were excluded (837 with unknown HIV status for the mortality risk outcome [193 in the intervention arm and 644 in the routine care arm], 6 deaths in the intervention arm and 15 deaths in the routine care arm). The p-value for the overall adjusted risk ratio = 0·34.

***Figure S2. Adjusted mortality rate ratios and six-month mortality risk ratios***

**Mortality rate over 18 months (N=8413)**

**Six-month mortality risk (N=8116)**

******

***Table S6. Cluster-level mortality rate and six-month mortality risk outcomes***

|  | **Mortality rate outcome**  **N=8413** | | | | **Six-month mortality risk outcome**  **N=8116** | | |
| --- | --- | --- | --- | --- | --- | --- | --- |
|  | **Patients** | **Person-years** | **Observed deaths*** | **Unadjusted mortality rate per 100 person-years** | **Patients** | **Observed deaths** | **Unadjusted mortality risk (%)** |
| **Intervention** | 4556 | 3654·8 | 145 | 3·83† | 4421 | 116 | 2·45† |
| Health Center 1 | 609 | 505·6 | 29 | 5·74 | 597 | 26 | 4·36 |
| Health Center 2 | 345 | 252·6 | 4 | 1·58 | 331 | 3 | 0·91 |
| Health Center 3 | 628 | 501·5 | 23 | 4·59 | 615 | 17 | 2·76 |
| Health Center 4 | 633 | 517·8 | 18 | 3·48 | 624 | 11 | 1·76 |
| Health Center 5 | 149 | 120·3 | 8 | 6·65 | 145 | 7 | 4·83 |
| Health Center 6 | 632 | 477·4 | 22 | 4·61 | 619 | 19 | 3·07 |
| Health Center 7 | 341 | 223·3 | 11 | 4·93 | 323 | 10 | 3·10 |
| Health Center 8 | 212 | 143·7 | 12 | 8·35 | 192 | 11 | 5·73 |
| Health Center 9 | 560 | 502·3 | 11 | 2·19 | 544 | 6 | 1·10 |
| Health Center 10 | 447 | 410·0 | 7 | 1·71 | 431 | 6 | 1·39 |
| **Routine care** | 3857 | 3015·3 | 154 | 4·88† | 3695 | 123 | 3·12† |
| Health Center 11 | 683 | 474·0 | 39 | 8·22 | 640 | 30 | 4·69 |
| Health Center 12 | 494 | 338·4 | 25 | 7·39 | 471 | 22 | 4·67 |
| Health Center 13 | 386 | 350·6 | 17 | 4·85 | 376 | 13 | 3·46 |
| Health Center 14 | 760 | 697·1 | 17 | 2·44 | 753 | 11 | 1·46 |
| Health Center 15 | 163 | 127·1 | 5 | 3·93 | 148 | 5 | 3·38 |
| Health Center 16 | 503 | 401·1 | 11 | 2·74 | 489 | 9 | 1·84 |
| Health Center 17 | 161 | 113·3 | 7 | 6·18 | 158 | 6 | 3·80 |
| Health Center 18 | 241 | 172·6 | 8 | 4·63 | 224 | 7 | 3·13 |
| Health Center 19 | 238 | 171·7 | 20 | 11·65 | 226 | 17 | 7·52 |
| Health Center 20 | 228 | 169·4 | 5 | 2·95 | 210 | 3 | 1·43 |

Footnote:

*26/145 (18%) of patients that died in the intervention arm and 21/154 (14%) in the routine care arm had confirmed TB with 14 days

†Geometric mean of the rate/risk across the clusters

***Table S7. Adjusted mortality rate difference and six-month mortality risk difference***

**Mortality rate difference over 18 months (N=8413)**

|  | *Intervention* | | *Routine care* | | *Adjusted Rate Difference (95%CI) †* |
| --- | --- | --- | --- | --- | --- |
|  | Deaths Observed/  Person-years | Rate* | Deaths Observed/  Person-years | Rate* |  |
| Overall | 145 / 3654·8 | 3·83 | 154/ 3015·3 | 4·88 | -1·20 (-3·69—1·29) |
| HIV status |  |  |  |  |  |
| Positive | 99 / 1570·6 | 6·72 | 76 / 1348·6 | 6·10 | 1·16 (-3·24—5·57) |
| Negative | 46 / 2084·2 | 2·16 | 78 / 1666·7 | 3·71 | -3·25 (-6·18—0·33) |
| Sex |  |  |  |  |  |
| Female | 63 / 2178·0 | 2·36 | 81 / 1850·7 | 3·70 | -1·47 (-3·99—1·05) |
| Male | 82 / 1476·8 | 5·56 | 73 / 1164·6 | 5·43 | -0·74 (-4·35—2·86) |

**Six-month mortality risk difference (N=8116)**

|  | *Intervention* | | *Routine care* | | *Adjusted Risk Difference (95%CI) †* |
| --- | --- | --- | --- | --- | --- |
|  | Deaths Observed/  N | Risk* (%) | Deaths Observed/  N | Risk* (%) |  |
| Overall | 116 / 4421 | 2·45 | 123 / 3695 | 3·12 | -0·70% (-2·41—1·01) |
| HIV status |  |  |  |  |  |
| Positive | 78 / 1935 | 3·81 | 59 / 1673 | 3·91 | 0·50% (-2·49—3·49) |
| Negative | 38 / 2486 | 1·50 | 64 / 2022 | 2·74 | -1·87% (-3·75—0·00) |
| Sex |  |  |  |  |  |
| Female | 48 / 2576 | 1·45 | 63 / 2238 | 2·75 | -0·93% (-2·66—0·80) |
| Male | 68 / 1845 | 3·50 | 60 / 1457 | 3·62 | -0·35% (-2·75—2·05) |

Footnote:

*Unadjusted rates and risks in the intervention and routine care arms are the geometric mean of the point estimates across the ten clusters in each group. Rates are reported per 100 person-years.

†Adjusted rate and risk ratios compare the intervention vs. routine care arms and analysis was done at the cluster-level with adjustment for randomization strata (fixed effect, two levels), number of patients treated for TB in the prerandomization period, and patient-level covariates (age, sex, and HIV status). Individuals with unknown HIV status were excluded (855 with unknown HIV status for the mortality rate outcome [199 in the intervention arm and 656 in the routine care arm], 7 deaths / 164·0 person-years in the intervention arm and 19 deaths / 539·2 person-years in the routine care arm; 837 with unknown HIV status for the six-month mortality risk outcome [193 in the intervention arm and 644 in the routine care arm], 6 deaths in the intervention arm and 15 deaths in the routine care arm).

***Table S8. Sensitivity analyses for mortality rate and six-month mortality risk outcomes***

**Mortality rate over 18 months**

|  | Adjusted Rate Ratio (95%CI) | Adjusted Rate Difference (95%CI) |
| --- | --- | --- |
| Manuscript definition of vital status outcomes* | 0·77 (0·47—1·28) | -1·20 (-3·69—1·29) |
| Modified definition of vital status outcomes: Prioritize phone then home visit outcomes if both are available | 0·77 (0·46—1·26) | -1·21 (-3·69—1·26) |
| Modified definition of vital status outcomes: Prioritize register then phone call outcomes if both are available | 0·73 (0·46—1·17) | -1·44 (-3·88—1·00) |
| Exclude patients with missing or invalid date of death† | 0·79 (0·47—1·32) | -1·06 (-3·43—1·30) |
| Include patients with unknown HIV status‡ | 0·80 (0·48—1·34) | -1·06 (-3·54—1·42) |
| Include patients with unknown HIV status and adjust for HIV status (Negative/Positive/Unknown)§ | 0·82 (0·48—1·37) | -1·00 (-3·53—1·53) |

**Six-month mortality risk**

|  | Adjusted Risk Ratio (95%CI) | Adjusted Risk Difference (95%CI) |
| --- | --- | --- |
| Manuscript definition of vital status outcomes* | 0·77 (0·44—1·35) | -0·70% (-2·41—1·01) |
| Modified definition of vital status outcomes: Prioritize phone then home visit outcomes if both are available | 0·77 (0·44—1·35) | -0·70% (-2·41—1·01) |
| Modified definition of vital status outcomes: Prioritize register then phone call outcomes if both are available | 0·76 (0·43—1·33) | -0·77% (-2·49—0·95) |
| Exclude patients with missing or invalid date of death† | 0·76 (0·43—1·32) | -0·69% (-2·28—0·90) |
| Include patients with unknown HIV status‡ | 0·81 (0·45—1·44) | -0·55% (-2·28—1.17) |
| Include patients with unknown HIV status and adjust for HIV status (Negative/Positive/Unknown)§ | 0·82 (0·46—1·47) | -0·52% (-2·29—1·24) |
| Expand follow-up window to 4-8 months post-tuberculosis evaluation¶ | 0·78 (0·45—1·36) | -0·64% (-0·23—0·97) |

Footnote:

* For a subset of patients with multiple sources of outcome data and discrepant dates of death documented, the home visit data was prioritized if available, then phone call data was the next priority. For patients marked as having died but subsequently marked as alive by additional follow-up, the same prioritization scheme was applied.

† In the manuscript definition, for patients known to have died but with an unknown death date, the death date was assigned to be the midpoint between the date the patient was last known to be alive (smear or Xpert date), and the date the patient was first known to have died, based on a home visit, phone call, or the TB treatment register. Instead, here we exclude those patients (N=8391 for mortality rate outcome; N=8094 for mortality risk outcome).

‡ In the manuscript definition, we exclude patients with unknown HIV status. Instead, here we have included those patients and adjusted for only two patient-level covariates (age and sex), in addition to randomization strata (fixed effect, two levels) and number of individuals treated for TB in the pre-randomization period (N=9268 for mortality rate outcome; N=8953 for mortality risk outcome). The p-value for the adjusted rate ratio = 0·38; the p-value for the adjusted risk ratio = 0·45.

§ In the manuscript definition, we exclude patients with unknown HIV status. Instead, here we have included those patients and adjusted for HIV status at the patient-level with three categories: Negative, Positive, Unknown. The other adjustments align with the analysis reported in the manuscript: patient-level covariates (age and sex), randomization strata (fixed effect, two levels), and number of individuals treated for TB in the pre-randomization period (N=9268 for mortality rate outcome; N=8953 for mortality risk outcome). The p-value for the adjusted rate ratio = 0·42; the p-value for the adjusted risk ratio = 0·54.

**¶** In the manuscript definition, we limit follow-up to 5-8 months after TB diagnostic evaluation. Instead, here we adjust the follow-up window to include those with vital status outcomes 4-8 months after TB diagnostic evaluation, excluding those last known to be alive <121 days after TB diagnostic evaluation (N=8373).
